# Supplementary material for: Ungulate presence and predation risks reduce acorn predation by mice in dehesas
Source: PLoS One. 2022 Aug 15;17(8):e0260419. doi: 10.1371/journal.pone.0260419 (PMC9377575; doi:10.1371/journal.pone.0260419)
Supplement: S1 File — (DOCX) [file pone.0260419.s002.docx]

**S1. Structure of models and priors**

Acorn selection model

We modelled acorn selection as a multinomial process. The probability of selection of the i-th acorn from the t-th tree during the k-th event depended on acorn size ($S_{i}$ availability ($A_{k})$ the presence of predator scent ($P_{t}$) or competition with ungulates ($U_{t}$) on the tree where it is located; as well as the moon phase ($M_{k}$) and month ($T_{k})$ during the k-th event. Size-driven acorn choices were modified by its interactions with environmental variables. The basal probability of selection (${}_{0_{t}}$) depended on the focal tree where the event occurred.

| $y_{i,t,k} \sim Multinomial (p_{i,t,k}, 1)$ | Eq. A1 |
| --- | --- |
| $p_{i,t,k}=\frac{e_{i,t,k}}{\sum_{i}^{N_{i,t,k}} e_{i,t,k}}$ | Eq. A2 |
| $log\left( e_{i,t,k} \right)= {}_{0_{t}}+{}_{1}S_{i}+ {}_{2}A_{k}+{}_{3}P_{t}+{}_{4}U_{t}+{}_{5}M_{k}+{}_{6}T_{k}+{}_{7}S_{i}A_{K}+{}_{8}S_{i}P_{t}+{}_{9}S_{i}U_{T}+{}_{10}S_{i}M_{K}+{}_{11}S_{i}T_{k}$ | Eq. A3 |
| ${}_{0_{t}} \sim Normal\left( {}_{0},\sigma_{0} \right)$ | Eq. A4 |

$y_{i,t,k}$ is a binary variable that represents whether the acorn *i* a thet tree *t* and foraging event *k* was selected (1), or not (0). N represents the number of acorns present during the foraging event. Local availability of acorns during the k-th event was measured as $A_{k}=\sum_{i=1}^{N} S_{i}$. Acorn size and local availability were scaled (mean = 0, sd= 1) before analyses. We used uninformative priors, the expected intercept (${}_{0}$), the effects of acorn size, environmental covariates and their double interactions (${}_{1-9}$ ) were drawn from a normal distribution with mean 0 and standard deviation 10. Standard deviation of tree-level intercept was sampled from a uniform distribution (min = 0, max = 100). We run 10^4^ interations in three chains and checked for convergence (Rhat < 1.1) and Neff (>500).

Acorn removal model

We modelled the probability of removal of a selected acorn as a Bernoulli process. The probability that a selected acorn from the t-th tree is removed during the k-th event depends on acorn size ($S_{i}$) and availability ($A_{k})$ the presence of predator scent ($P_{t}$) or competition with ungulates ($U_{t}$) on the tree where it is located; as well as the moon phase ($M_{k}$) and month ($T_{k})$ during the k-th event. Acorn size effects on removal rates were modified by its interactions with environmental variables. The basal probability of selection (${}_{0_{t}}$) depended on the focal tree where the event occurred.

| $y_{i,t,k} \sim Bern (p_{i,t,k})$ | Eq. A5 |
| --- | --- |
| $logit\left( p_{i,t,k} \right)= {}_{0_{t}}+{}_{1}S_{i}+ {}_{2}A_{k}+{}_{3}P_{t}+{}_{4}U_{t}+{}_{5}M_{k}+{}_{6}T_{k}+{}_{7}S_{i}A_{K}+{}_{8}S_{i}P_{t}+{}_{9}S_{i}U_{T}+{}_{10}S_{i}M_{K}+{}_{11}S_{i}T_{k}$ | Eq. A6 |
|  |  |
| ${}_{0_{t}} \sim Normal\left( {}_{0},\sigma_{0} \right)$ | Eq. A7 |

$y_{i,t,k}$ is a binary variable that represents whether the acorn i-th acorn from the t-th tree during the foraging event *k* was removed (1), or not (0). Local availability of acorns during the k-th event was measured as $A_{k}=\sum_{i=1}^{N} S_{i}$. Acorn size and local availability were scaled (mean = 0, sd= 1) before analyses. We used uninformative priors, the expected intercept (${}_{0}$), the effects of acorn size, environmental covariates and their double interactions (${}_{1-9}$ ) were drawn from a normal distribution with mean 0 and standard deviation 10. Standard deviation of tree-level intercept was sampled from a uniform distribution (min = 0, max = 100). We run 10^4^ interations in three chains and checked for convergence (Rhat < 1.1) and Neff (>500).

Acorn mobilization model

We modelled acorn mobilization distances (log-transformed, cm) as a gaussian hierarchical regression. The distance that a selected acorn from the t-th tree is mobilized during the k-th event depends on acorn size ($S_{i}$) and availability ($A_{k})$ the presence of predator scent ($P_{t}$) or competition with ungulates ($U_{t}$) on the tree where it is located; as well as the moon phase ($M_{k}$) and month ($T_{k})$ during the k-th event. Acorn size effects on mobilization distances were modified by its interactions with environmental variables. The basal mobilization distance (${}_{0_{t}}$) depended on the focal tree where the event occurred.

| $log(y_{i,t,k}) \sim Norm (\mu_{i,t,k}, \sigma)$ | Eq. A8 |
| --- | --- |
| $\mu_{i,t,k}= {}_{0_{t}}+{}_{1}S_{i}+ {}_{2}A_{k}+{}_{3}P_{t}+{}_{4}U_{t}+{}_{5}M_{k}+{}_{6}T_{k}+{}_{7}S_{i}A_{K}+{}_{8}S_{i}P_{t}+{}_{9}S_{i}U_{T}+{}_{10}S_{i}M_{K}+{}_{11}S_{i}T_{k}$ | Eq. A9 |
| ${}_{0_{t}} \sim Normal\left( {}_{0},\sigma_{0} \right)$ | Eq. A10 |

$y_{i,t,k}$ is the mobilization distance (in cm and log-transformed) that the i-th acorn from the t-th tree was mobilized. Local availability of acorns during the k-th event was measured as $A_{k}=\sum_{i=1}^{N} S_{i}$. Acorn size and local availability were scaled (mean = 0, sd= 1) before analyses. We used uninformative priors, the expected intercept (${}_{0}$), the effects of acorn size, environmental covariates and their double interactions (${}_{1-9}$ ) were drawn from a normal distribution with mean 0 and standard deviation 10. Standard deviations ($\sigma$ and $\sigma_{0} )$ were sampled from a uniform distribution (min = 0, max = 100). We run 10^4^ interations in three chains and checked for convergence (Rhat < 1.1) and Neff (>500).

Acorn fate model

We modelled the probability that a removed acorn is deposited in viable status as a Bernoulli process. The probability that a removed acorn from the t-th tree during the k-th event is viable after deposition depends on acorn size ($S_{i}$) and availability ($A_{k})$ the presence of predator scent ($P_{t}$) or competition with ungulates ($U_{t}$) on the tree where it is located; as well as the moon phase ($M_{k}$) and month ($T_{k})$ during the k-th event. Acorn size effects on removal rates were modified by its interactions with environmental variables. The basal probability of being deposited healthy (${}_{0_{t}}$) depended on the focal tree where the event occurred.

| $y_{i,t,k} \sim Bern (p_{i,t,k})$ | Eq. A11 |
| --- | --- |
| $logit\left( p_{i,t,k} \right)= {}_{0_{t}}+{}_{1}S_{i}+ {}_{2}A_{k}+{}_{3}P_{t}+{}_{4}U_{t}+{}_{5}M_{k}+{}_{6}T_{k}+{}_{7}S_{i}A_{K}+{}_{8}S_{i}P_{t}+{}_{9}S_{i}U_{T}+{}_{10}S_{i}M_{K}+{}_{11}S_{i}T_{k}$ | Eq. A12 |
| ${}_{0_{t}} \sim Normal\left( {}_{0},\sigma_{0} \right)$ | Eq. A13 |

$y_{i,t,k}$ is a binary variable that represents whether the acorn i-th acorn from the t-th tree during the foraging event *k* was viable (1), or predated (0). Local availability of acorns during the k-th event was measured as $A_{k}=\sum_{i=1}^{N} S_{i}$. Acorn size and local availability were scaled (mean = 0, sd= 1) before analyses. We used uninformative priors, the expected intercept (${}_{0}$), the effects of acorn size, environmental covariates and their double interactions (${}_{1-9}$ ) were drawn from a normal distribution with mean 0 and standard deviation 10. Standard deviation of tree-level intercept was sampled from a uniform distribution (min = 0, max = 100). We run 10^4^ interations in three chains and checked for convergence (Rhat < 1.1) and Neff (>500).
