# Supplementary material for: Ungulate presence and predation risks reduce acorn predation by mice in dehesas
Source: PLoS One. 2022 Aug 15;17(8):e0260419. doi: 10.1371/journal.pone.0260419 (PMC9377575; doi:10.1371/journal.pone.0260419)
Supplement: S2 File — (DOCX) [file pone.0260419.s003.docx]

**S2. Posterior predictive checks**

**
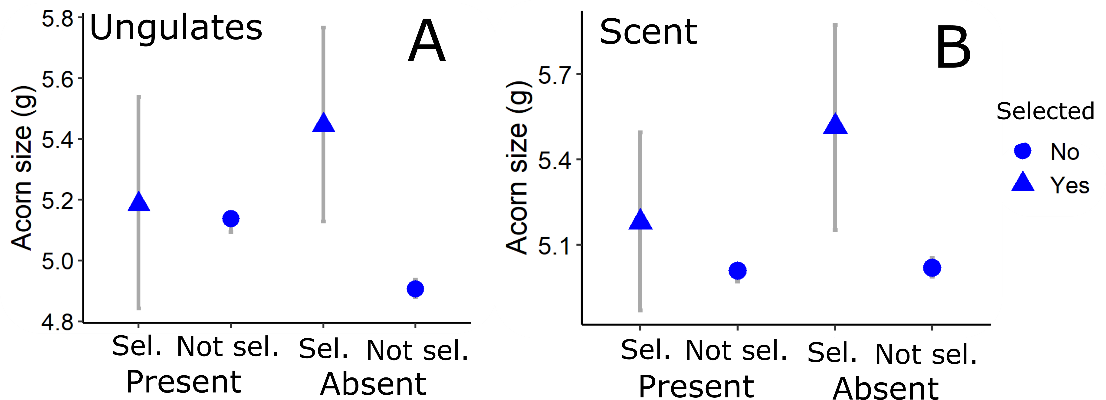
**

Fig S2_1. Posterior predictive check of the multinomial model for acorn selection. Effects of (A) ungulate and (B) predator scent presence are plotted. Blue dots represent mean values of data bars represent credible intervals of model predictions (across 5000 simulations).


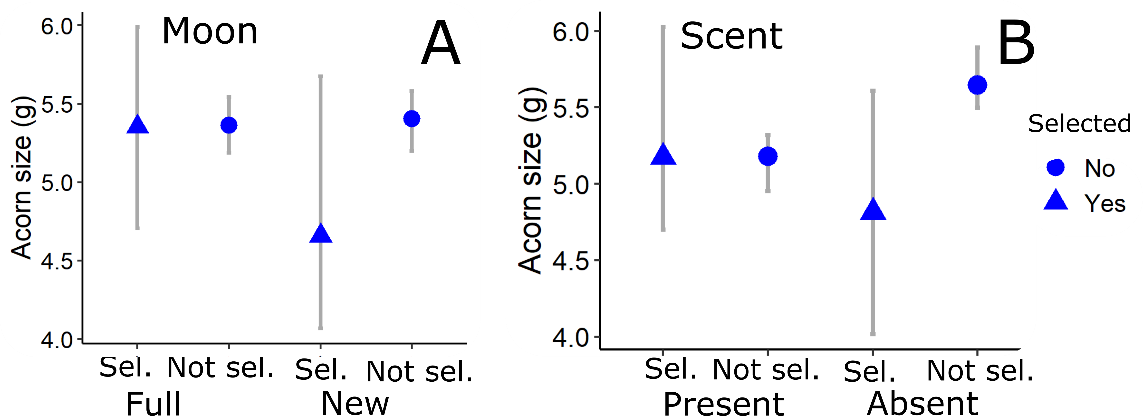


Fig S2_2. Posterior predictive check of the bernoulli model for acorn removal. Effects of (A) moon phase and (B) predator scent presence are plotted. Blue dots represent mean values of data bars represent credible intervals of model predictions (across 5000 simulations).


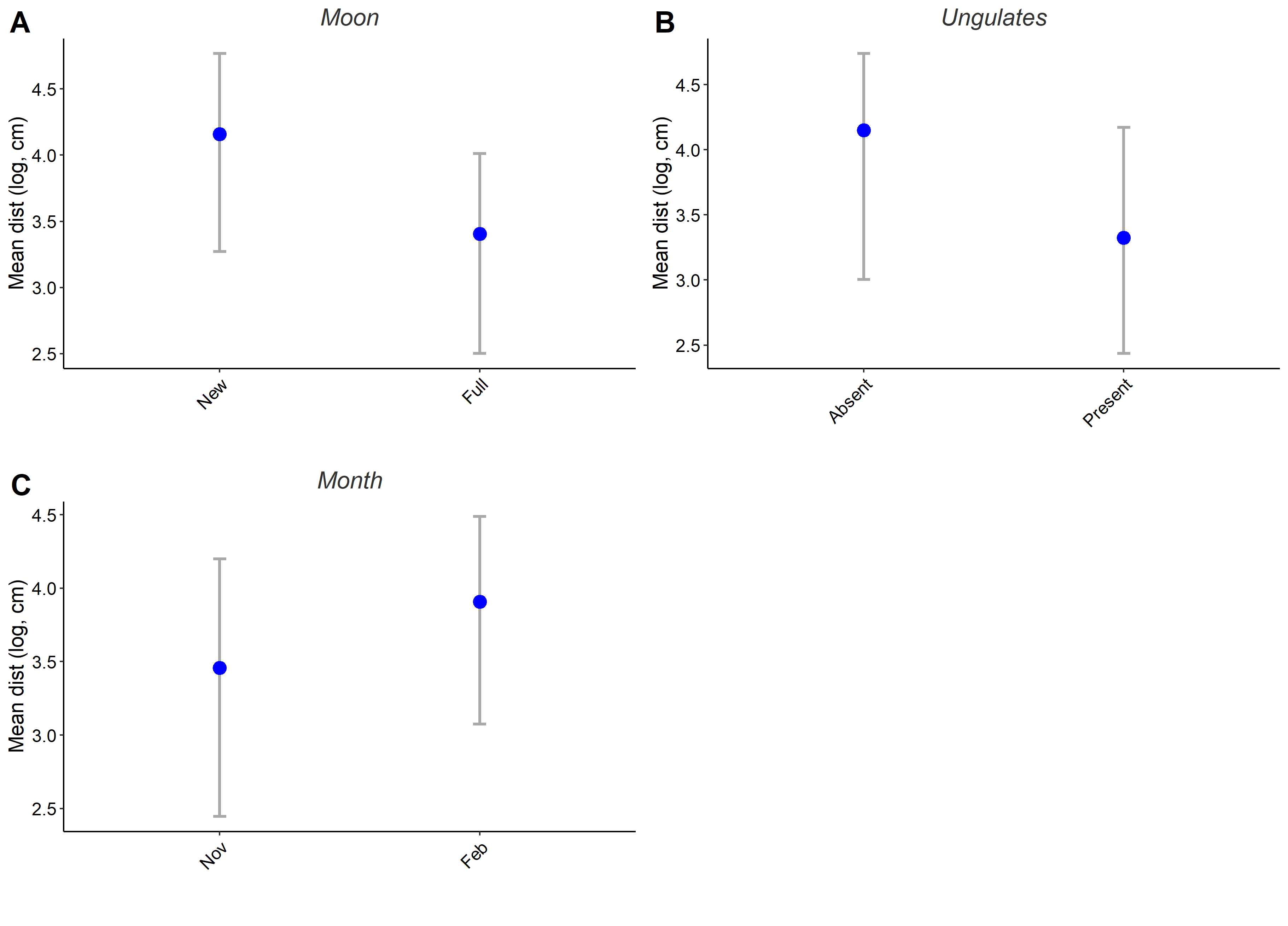


Fig S2_3. Posterior predictive check of the regression model for acorn mobilization distances. Blue dots represent mean values of data bars represent credible intervals of model predictions (across 5000 simulations).


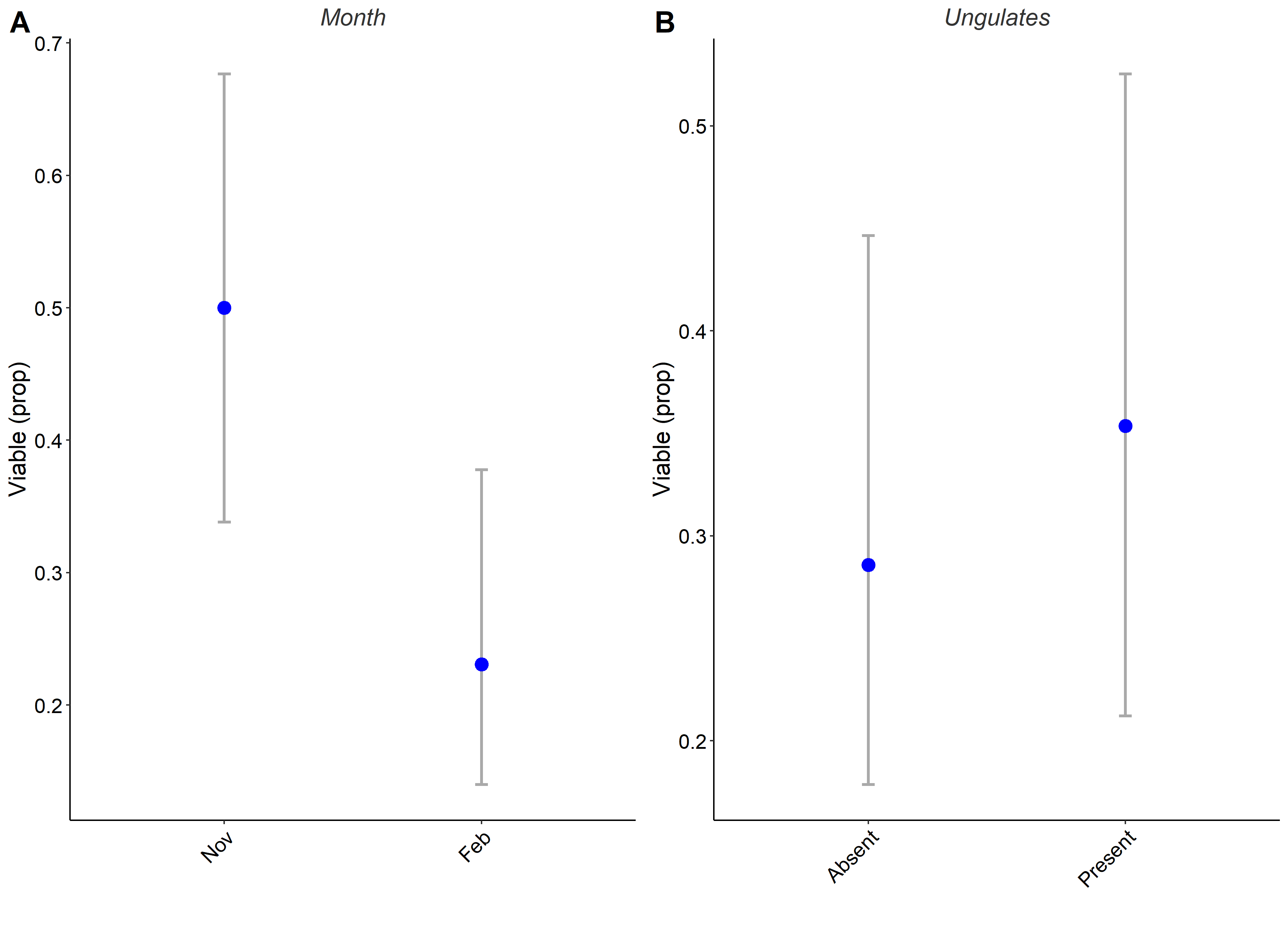


Fig S2_4. Posterior predictive check of the regression model for acorn fate. Effects of (A) month and (B) ungulate presence are plotted. Blue dots represent mean values of data bars represent credible intervals of model predictions (across 5000 simulations).
