# Supplementary material for: Ungulate presence and predation risks reduce acorn predation by mice in dehesas
Source: PLoS One. 2022 Aug 15;17(8):e0260419. doi: 10.1371/journal.pone.0260419 (PMC9377575; doi:10.1371/journal.pone.0260419)
Supplement: S3 File — (DOCX) [file pone.0260419.s004.docx]

**S3. Specifications of transition probability model for acorn dispersal**

To estimate the joint effect of seed size environmental covariates and their double interactions on the probability acorn dispersal, we developed a mechanistic transition probability model. In our model, simulated mice encounter acorns in an experimental setup similar to ours. Then, they decide (i) which acorn to select, (ii) whether to remove it or consume it *in situ*; and if mobilized, (iii) whether to predate or deposit it.

Transition probabilities across dispersal stages are modulated by acorn size, the moon phase and local environmental covariates (i.e. acorn availability, presence of ungulates and predator scent). To this end, previous to simulations we parameterized regressions of acorn selection, removal and deposition, as described above (eq. A1-A13). Nonetheless, in this case we only used the subset of data corresponding to November, when most acorn fall occurs in our study system. Therefore, in our regression (and simulations) month was not included as a covariate. In each submodel, we run 3 chains with 50000 iterations to obtain posterior distribution of their parameters ($\hat{\phi}_{s}$ “s” depicts foraging submodel: 1-selection, 2-removal and 3-deposition, hereafter). We used the same priors as described in Supplementary File 1, and checked for convergence (Rhat<1.1) and Neff (>500).

The model setup mimics our experimental design: 32 focal trees (half inside exclosures and half outside them) paired in treatments of predator scent (presence/absence). Simulations being under new moon conditions with focal trees offering 15 acorns of large, medium and small sizes (5 each). Acorn size is sampled from empirical distributions of these size categories. The number of foraging events taking place in each focal tree is drawn from a Poison distribution with mean equal to $\bar{\lambda_{m}}$. $\bar{\lambda_{m}}$ depicts the average number of events observed in the corresponding moon phase. Before running the n-th simulation, a parameter-set is sampled from posterior distributions of behavior submodels ($\hat{\phi}_{1_{i}}$, $\hat{\phi}_{2_{i}}$, $\hat{\phi}_{3_{i}}$). In all cases $\hat{\phi}$ contains the effects of acorn size as well environmental covariates (acorn availability, moon phase, predator scent and ungulate presence and their double interaction with size) ($\hat{ϴ})$, as well as tree-level intercepts ($\hat{\tau_{.}}$).

During the n-th simulation and the k-th foraging event of the th-tree, a focal mouse decides which i-th acorn to select by sampling among those available, with a probability $p_{i,t,k}={e_{i,t,k}}/{\sum_{l=1}^{L} e_{i,t,k}}$ being $log\left( e_{i,t,k} \right)=\hat{\tau_{t}}+\sum_{p=0}^{P} ϴ_{p_{1[n]}}$. L is the number of acorns available. $\tau_{t}$ depicts the intercept for the t-th focal tree and P represents the number of parameters present in the $ϴ_{p_{1[n]}}$ vector (size, environmental covariates and their double-interaction with size). Subsequently, the mouse decides whether to remove the acorn or not following $Z_{i,t,k}\sim Bern\left( \psi_{i,t,k} \right)$, being $Z_{i,t,k}=1$ if the acorn is removed and 0 otherwise. The probability of acorn removal ($\psi_{i,t,k})$ is modeled as $logit\left( \psi_{i,t,k} \right)=\tau_{t}+\sum_{p=0}^{P} ϴ_{p_{2[n]}}$. Finally, if the acorn is mobilized, the simulated mouse decides whether to deposit or predate it as ${Z'}_{i,t,k}\sim Bern\left( \gamma_{i,t,k} \right)$. ${Z'}_{i,t,k}$ depicts if the acorn is predated (0) or not (1). The probability of deposition in a viable status is modeled as $logit\left( ϒ_{i,t,k} \right)=\tau_{t}+\sum_{p=0}^{P} ϴ_{p_{3[n]}}$. See Fig. C1 for an overview of the foraging decisions processes.

Once all foraging events from all focal trees have occurred during the new moon phase, the same procedure is repeated for full moon conditions. Simulation of acorn dispersal under new and full conditions represent one model run. After each model run the program tracks the size and status of selected acorns and the environmental covariates in which the foraging event occurred. We run the model 10^3^ times.


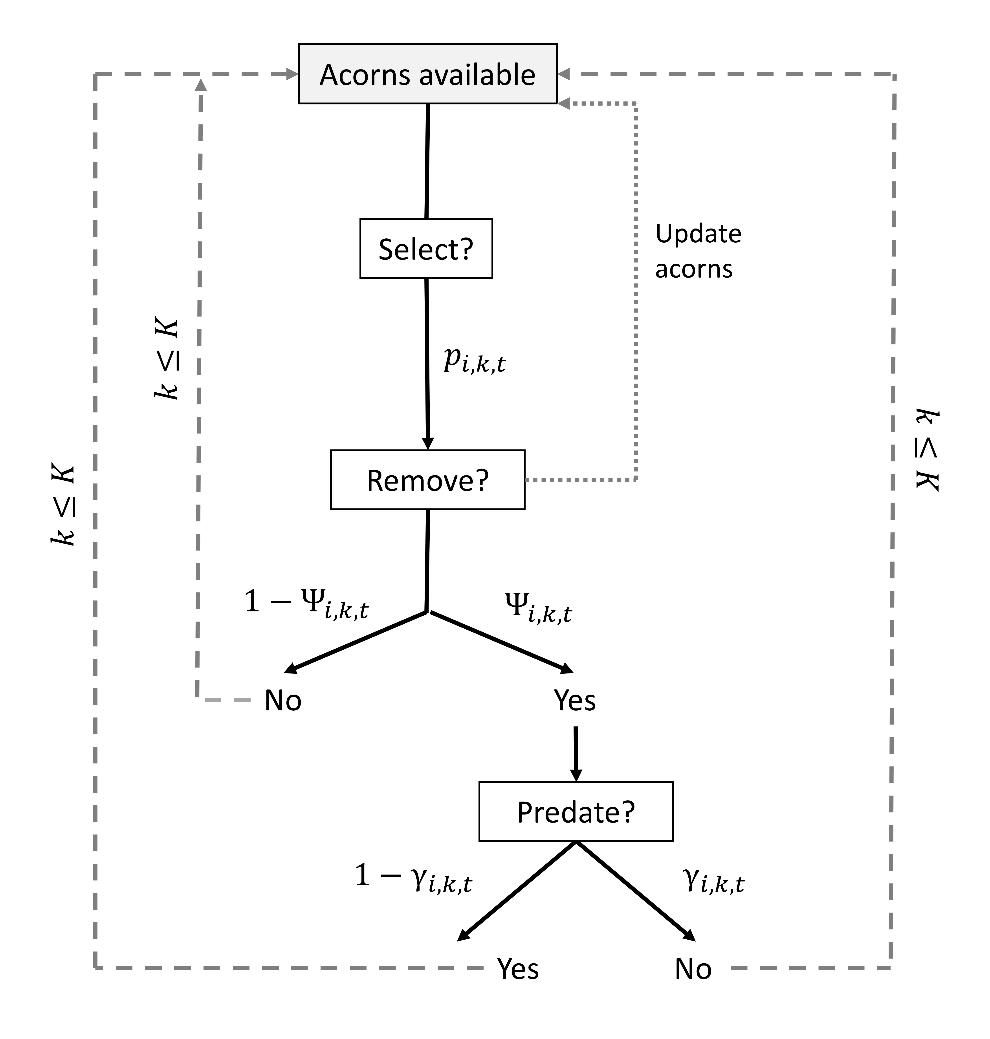


Fig. S3_1. Process overview of the transition probability model of acorn dispersal. In a focal tree a simulated mouse encounters acorn of different sizes. It selects one to handle according to the probability $p_{i,t,k}$. Subsequently, it decides whether to remove it or not based on $\psi_{i,t,k}$; and if removed whether to predate it or not following the probability $\gamma_{i,t,k}$. A foraging event can stop in each of these steps. Once it stops, another foraging event takes place. Every time an acorn is removed, the model updates the number of acorns available in the focal tree. A model run consists on simulations of all foraging events (k = K) of each focal tree under new and full moon conditions.
