# Supplementary material for: Ungulate presence and predation risks reduce acorn predation by mice in dehesas
Source: PLoS One. 2022 Aug 15;17(8):e0260419. doi: 10.1371/journal.pone.0260419 (PMC9377575; doi:10.1371/journal.pone.0260419)
Supplement: S4 File — (DOCX) [file pone.0260419.s005.docx]

**S4.** **Code for the transition probability model**

#Transition probability model which connects all stages of scatter-hoardering process

#and in which simulated mice adapt their behaviour to environmental conditions

#(i.e. moon light, presence of ungulate, predator scent and acorn availability)

rm(list=ls())

library(jagsUI)

library(ggplot2)

library(coda)

#Load posterior distributions of behavioral models

load("Selection_input.RData")#c

load("Remov_input.RData")

load("Fate_input.RData")

load("Stand.RData")#values before standarization (mean and sd)

load("Initial_conditions.RData")#acorns in trees and trees (scent treatment, ungulate presence...)

nsim = 1000

tot = length(sel_chain$sims.list$b1)

id_sim = sample(seq(1:tot), nsim)

#Create vectors of parameteres for selection

mu0_s = sel_chain$sims.list$mu0[id_sim]

s0_s= sel_chain$sims.lis$s0[id_sim]

b1_s= sel_chain$sims.lis$b1[id_sim]

b2_s= sel_chain$sims.lis$b2[id_sim]

b3_s= sel_chain$sims.lis$b3[id_sim]

b4_s= sel_chain$sims.lis$b4[id_sim]

b5_s= sel_chain$sims.lis$b5[id_sim]

b6_s= sel_chain$sims.lis$b6[id_sim]

b7_s= sel_chain$sims.lis$b7[id_sim]

b8_s= sel_chain$sims.lis$b8[id_sim]

b9_s= sel_chain$sims.lis$b9[id_sim]

#Create vectors of parameters for removal

tot = length(remov_chain$sims.list$b1)

id_sim = sample(seq(1:tot), nsim)

mu0_r = remov_chain$sims.list$mu0[id_sim]

s0_r= remov_chain$sims.lis$s0[id_sim]

b1_r= remov_chain$sims.lis$b1[id_sim]

b2_r= remov_chain$sims.lis$b2[id_sim]

b3_r= remov_chain$sims.lis$b3[id_sim]

b4_r= remov_chain$sims.lis$b4[id_sim]

b5_r= remov_chain$sims.lis$b5[id_sim]

b6_r= remov_chain$sims.lis$b6[id_sim]

b7_r= remov_chain$sims.lis$b7[id_sim]

b8_r= remov_chain$sims.lis$b8[id_sim]

b9_r= remov_chain$sims.lis$b9[id_sim]

#Create vectors of parameters for fate

tot = length(fate_chain$sims.list$b1)

id_sim = sample(seq(1:tot), nsim)

mu0_f = fate_chain$sims.list$mu0[id_sim]

s0_f= fate_chain$sims.lis$s0[id_sim]

b1_f= fate_chain$sims.lis$b1[id_sim]

b2_f= fate_chain$sims.lis$b2[id_sim]

b3_f= fate_chain$sims.lis$b3[id_sim]

b4_f= fate_chain$sims.lis$b4[id_sim]

b5_f= fate_chain$sims.lis$b5[id_sim]

b6_f= fate_chain$sims.lis$b6[id_sim]

b7_f= fate_chain$sims.lis$b7[id_sim]

b8_f= fate_chain$sims.lis$b8[id_sim]

b9_f= fate_chain$sims.lis$b9[id_sim]

# Simulate acorn selection, removal and fate ------------------------------------------------

nsim= 1000

outputs = vector("list", nsim)

outputs2 = vector("list", nsim)

for(s in 1:nsim)

{

OUT = vector("list", 2)#outputs for each moon light

OUT2 = vector("list", 2)

for(m in 1:2)## mstands for moonlight (new and full moon conditions)

{

M = m-1

X_m= X_tree[[m]]#covariates of trees

nt = nrow(A_init[[m]])#Number of trees with activity

Out = array(NA, c(nt, 2))#output, proportion of acorns carried away and healthy and their average weight

Out2 = array(NA, c(nt, 2))#Acorn size and fate (1 = survived, 0=consumed)

tmp_e = rpois(nt, ne[m] )#number of events per tree. ne holds average number under each new moon conditions

#sample tree intercept for each regression

B0_s = rnorm(nt, mean = mu0_s[s], sd = s0_s[s])#

B0_r = rnorm(nt, mean = mu0_r[s], sd= s0_r[s])#

B0_f = rnorm(nt, mean = mu0_f[s], sd = s0_f[s])#

for(t in 1:nt)#Loop for trees

{

size_sel = rep(NA, tmp_e[t])#Holds size of selected acorns

size_remov = rep(NA, tmp_e[t])#Holds size of removed acorns

id_a_ev = rep(NA, tmp_e[t])#acorn identity

avs = rep(NA, tmp_e[t])#Acorn availaiblity in each evnt

acorns = A_init[[m]][t,]

fate_events = rep(0, tmp_e[t]) #fate of acorns corresponding to each event

for(e in 1:tmp_e[t])

{

avs[e]= sum(acorns)

if(sum(acorns) > 0)#while there are available acorns...

{

# Decide which acorn to select --------------------------------------------

log_e = rep(NA, length(acorns))

for(aa in 1:length(acorns))

{

Size2 = (acorns[aa]-m_pesoS)/sd_pesoS#transform to the scaled value (in regressions x values are scaled)

av2 = (avs[e]-m_avS)/sd_avS#transform to the scaled value

log_e[aa] = B0_s[t]+b1_s[s]*Size2+

b2_s[s]*M+ b3_s[s]*X_m[t,1]+

b4_s[s]*X_m[t,2] + b5_s[s]*av2+

b6_s[s]*M*Size2+

b7_s[s]*X_m[t,1]*Size2+

b8_s[s]*X_m[t,2]*Size2+

b9_s[s]*av2*Size2

}#loop acorns

ee = exp(log_e)

probs = ee/sum(ee)

tmp = rmultinom(1, seq(1:length(acorns)), probs)#select one acorn

acor_sel = which(tmp == 1)#acorn selected

id_a_ev[e] = acor_sel#identity of acorn selected

tmp_size = acorns[acor_sel]

tmp_size2 = (tmp_size- m_pesoR)/sd_pesoR

size_sel[e] = tmp_size##size of acorn seeced

# Decide whether it is mobilized outside the cage or not ------------------

pr = plogis(B0_r[t]+b1_r[s]*tmp_size2+

b2_r[s]*M+ b3_s[s]*X_m[t,1]+

b4_r[s]*X_m[t,2] + b5_r[s]*av2+

b6_r[s]*M*tmp_size2+

b7_r[s]*X_m[t,1]*tmp_size2+

b8_r[s]*X_m[t,2]*tmp_size2+

b9_r[s]*av2*tmp_size2)

removed = rbinom(1, 1, pr)

if(removed == 1)

{

size_remov[e] = tmp_size

acorns = acorns[-acor_sel]#remove the acorn from the cage for the next event

}

}# if there are acorns

}#loop events

#Fate of removed acorns

if(length(which(!is.na(size_remov))) >0)#if they have removed an acorn

{

id = which(!is.na(size_remov))#id of events to know acorn availability

tmp_remov = size_remov[id]#size of removed acorn

tmp_fate = rep(NA, length(tmp_remov))

for(ii in 1:length(tmp_fate))

{

tmp_size2 = (tmp_remov[ii]-m_pesoF)/sd_pesoF

tmp_av = avs[id[ii]]

tmp_av2 = (tmp_av- m_avF)/sd_avF

pr = plogis(B0_f[t]+b1_f[s]*tmp_size2+

b2_f[s]*M+ b3_s[s]*X_m[t,1]+

b4_f[s]*X_m[t,2] + b5_f[s]*tmp_av2+

b6_f[s]*M*tmp_size2+

b7_f[s]*X_m[t,1]*tmp_size2+

b8_f[s]*X_m[t,2]*tmp_size2+

b9_f[s]*tmp_av2*tmp_size2)

viable = rbinom(1, 1, pr)

if(viable == 1)

{

tmp_fate[ii]= 1

fate_events[id[ii]] = 1

}else{

tmp_fate[ii]= 0

}#end loop if viable

}#end of loop acorns mobilized

id2 = which(tmp_fate == 1)

if(length(id2) > 0)#if there are acorns that have fate = 1 (movilized and survived)

{

Out[t,] = c(sum(tmp_fate)/length(A_init[[m]][t,]) , mean(tmp_remov[id2]))

size_f1 = mean(size_sel[which(fate_events == 1)])

size_f0 = mean(size_sel[which(fate_events == 0)])

Out2[t,] = c(size_f0, size_f1)

}else{

Out[t,] = c(0, NA)

Out2[t,] = c(mean(size_sel[which(fate_events == 0)]), NA)

}

}else{

Out[t,] = c(NA, NA)#NA if there was no acorn mobilized

Out2[t,] = c(mean(size_sel[which(fate_events == 0)]), NA)

}#end if there was an acorn mobilized

}#loop trees

Out = cbind(Out, X_m)

colnames(Out) = c("prop_fate1", "size_fate1", "Ungulates", "Scent")

OUT[[m]] = Out

Out2 = cbind(Out2, X_m)

colnames(Out2) = c("size_fate0", "size_fate1", "Ungulates", "Scent")

OUT2[[m]] = Out2

}#loop moon

outputs[[s]] = OUT

outputs2[[s]] = OUT2

}#loop repetitions in the simulations

save(outputs, outputs2, file = "Simulation_ouputs.RData")

#outputs = proportion of acorns that were mobilized and survived as well as their mean weight according to the moon [[m]], ungulate and scent presence

#outputs2 = size of predated acorns vs those with fate = 1, as a funciton of moon [[m]], scent and predator presence
