# Supplementary material for: Ungulate presence and predation risks reduce acorn predation by mice in dehesas
Source: PLoS One. 2022 Aug 15;17(8):e0260419. doi: 10.1371/journal.pone.0260419 (PMC9377575; doi:10.1371/journal.pone.0260419)
Supplement: S5 File — (DOCX) [file pone.0260419.s006.docx]

**S5. Differences in vegetation structure inside and outside ungulate exclosures**


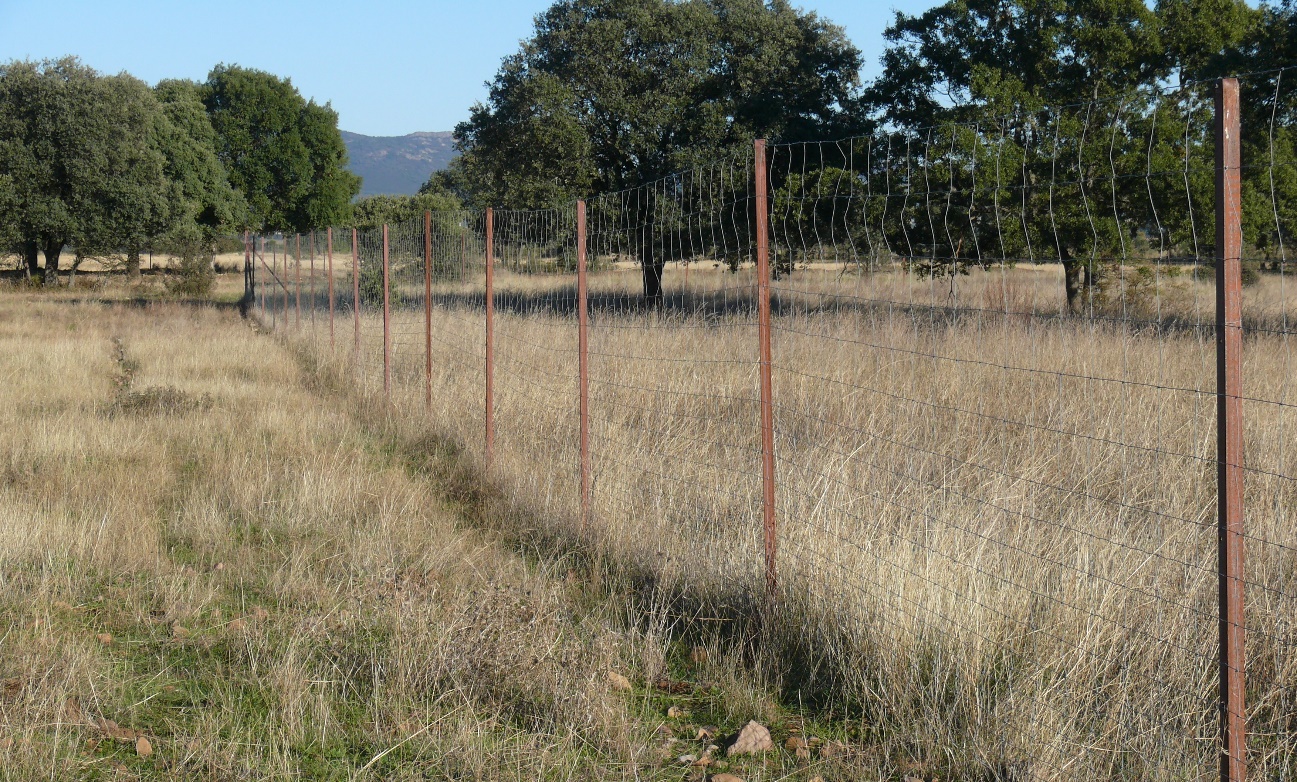


Fig. S5_1. Photograph of vegetation cover inside (right) and outside (left) ungulate exclosure.
