# Supplementary material for: Ungulate presence and predation risks reduce acorn predation by mice in dehesas
Source: PLoS One. 2022 Aug 15;17(8):e0260419. doi: 10.1371/journal.pone.0260419 (PMC9377575; doi:10.1371/journal.pone.0260419)
Supplement: Attachment — Submitted filename: PONE-D-21-34252_R1_LD review.pdf [file pone.0260419.s008.pdf]

PLOS ONE

Biological integrity of dehesa ecosystems favors acorn dispersal over predation in the mouse-oak mutualism

--Manuscript Draft--

**Manuscript Number:**	PONE-D-21-34252R1
**Article Type:**	Research Article
**Full Title:**	Biological integrity of dehesa ecosystems favors acorn dispersal over predation in the mouse-oak mutualism
**Short Title:**	Predation and competition favor dispersal in mouse-oak interaction
**Corresponding Author:**	Mario Díaz, Dr.<br>CSIC: Consejo Superior de Investigaciones Cientificas<br>Madrid, SPAIN
**Keywords:**	synzoochory; scatter-hoarding; competition; Risk; seed fate
**Abstract:**	Scatter-hoarding decisions by rodents are key for the long-term maintenance of oak populations in which avian seed dispersers are absent or inefficient. Decisions are determined by seed value, competition and predation risk. Therefore, they can be influenced by the integrity of the biological system (i.e. trees, rodents, ungulate competitors, and rodent predators). In this work, we manipulate and model the mouse-oak interaction in a Spanish dehesa, an anthropogenic savanna system in which nearby areas can show contrasting levels of mice and ungulate densities, as well as antipredatory cover. First, we conducted a large-scale cafeteria field experiment, where we modified ungulate presence and predation risk, and followed mouse scatter-hoarding decisions under contrasting levels of moonlight and acorn availability. Then, we estimated the net effects of competition and risk by means of a transition probability model that simulated mouse scatter-hoarding decisions. Our experimental and simulation results show that mice are able to adapt their foraging decisions to the environmental context and that such changes affect the probability of acorn dispersal. Under stressful conditions (predation risks and presence of ungulates), mice foraged opportunistically mobilizing large and small seeds. In addition, lack of antipredatory cover around dehesa trees limited the transportation of acorns, but also precluded mice activities outside tree canopies. As a result, post-dispersal predation rates were reduced and large acorns had a higher probability to survive. Overall, our work points out that inter-specific interactions preventing efficient foraging by scatter-hoarders can promote acorn dispersal. Therefore, the maintenance of the full set of producers, seed dispersers and consumers, as well as predators of dispersers in ecosystems, may be key for promoting seed dispersal effectiveness in conditional mutualisms.
**Order of Authors:**	Mario Díaz, Dr.
	Teresa Morán-López
	Jesús Sánchez-Dávila
	Ignasi Torre
	Alvaro Navarro-Castilla
	Isabel Barja
**Response to Reviewers:**	1. Please ensure that your manuscript meets PLOS ONE's style requirements, including those for file naming. The PLOS ONE style templates can be found at https://journals.plos.org/plosone/s/file?id=wjVg/PLOSOne_formatting_sample_main_body.pdf and https://journals.plos.org/plosone/s/file?id=ba62/PLOSOne_formatting_sample_title_authors_affiliations.pdf<br><br>Done<br><br>2. In your Methods section, please provide additional information regarding the permits you obtained for the work. Please ensure you have included the full name of the

Powered by Editorial Manager® and ProduXion Manager® from Aries Systems Corporation

	authority that approved the field site access and, if no permits were required, a brief statement explaining why.<br><br>Done. No offcila permits were necessary, as we explained in the former version, now developed in lines 169-173<br><br>3. Thank you for stating the following in the Acknowledgments Section of your manuscript:<br>D. López, M. Fernández and C. L. Alonso helped during fieldwork. D. López, B. Ramos and M. de Pablo pre-processed the video recordings, and D. Gallego, D. Valero, A. Velasco, C. J. González and E. Sánchez visualized the recordings noting seed choices. Authorities of the Cabañeros National Park provided the official permissions to carry out field experiments. J. España provided common genet scats. This study is a contribution to the projects RISKDISP (CGL2009-08430) and VULGLO (CGL2010-22180-C03-03), funded by the Spanish Ministry of Economy, and REMEDINAL3-CM (S2013/MAE-2719), funded by the Autonomous Community of Madrid. We declare no conflict of interest.<br><br>We note that you have provided funding information. However, funding information should not appear in the Acknowledgments section or other areas of your manuscript. We will only publish funding information present in the Funding Statement section of the online submission form.<br>Please remove any funding-related text from the manuscript and let us know how you would like to update your Funding Statement. Currently, your Funding Statement reads as follows:<br>Done<br>MD. Grant number S2013/MAE-2719. REMEDINAL3-CM project, funded by the Autonomous Community of Madrid. NO<br><br>4. Please include captions for your Supporting Information files at the end of your manuscript, and update any in-text citations to match accordingly. Please see our Supporting Information guidelines for more information: http://journals.plos.org/plosone/s/supporting-information.<br><br>Done
**Additional Information:**	
**Question**	**Response**
**Financial Disclosure**<br><br>Enter a financial disclosure statement that describes the sources of funding for the work included in this submission. Review the submission guidelines for detailed requirements. View published research articles from *PLOS ONE* for specific examples.<br><br>This statement is required for submission and **will appear in the published article** if the submission is accepted. Please make sure it is accurate.	MD. Grant number S2013/MAE-2719. REMEDINAL3-CM project, funded by the Autonomous Community of Madrid. NO

Powered by Editorial Manager® and ProduXion Manager® from Aries Systems Corporation

**Unfunded studies**<br>Enter: *The author(s) received no specific funding for this work.*<br><br>**Funded studies**<br>Enter a statement with the following details:<br>• Initials of the authors who received each award<br>• Grant numbers awarded to each author<br>• The full name of each funder<br>• URL of each funder website<br>• Did the sponsors or funders play any role in the study design, data collection and analysis, decision to publish, or preparation of the manuscript?<br>• **NO** - Include this sentence at the end of your statement: *The funders had no role in study design, data collection and analysis, decision to publish, or preparation of the manuscript.*<br>• **YES** - Specify the role(s) played.<br><br>* typeset	
**Competing Interests**<br><br>Use the instructions below to enter a competing interest statement for this submission. On behalf of all authors, disclose any competing interests that could be perceived to bias this work—acknowledging all financial support and any other relevant financial or non-financial competing interests.<br><br>This statement is required for submission and **will appear in the published article** if the submission is accepted. Please make sure it is accurate and that any funding sources listed in your Funding Information later in the submission form are also declared in your Financial Disclosure statement.<br><br>View published research articles from *PLOS ONE* for specific examples.	The authors have declared that no competing interests exist.

**NO authors have competing interests**<br><br>Enter: *The authors have declared that no competing interests exist.*<br><br>**Authors with competing interests**<br><br>Enter competing interest details beginning with this statement:<br><br>*I have read the journal's policy and the authors of this manuscript have the following competing interests: [insert competing interests here]*<br><br>* typeset	
**Ethics Statement**<br><br>Enter an ethics statement for this submission. This statement is required if the study involved:<br><br>• Human participants<br>• Human specimens or tissue<br>• Vertebrate animals or cephalopods<br>• Vertebrate embryos or tissues<br>• Field research<br><br>Write "N/A" if the submission does not require an ethics statement.<br><br>General guidance is provided below. Consult the submission guidelines for detailed instructions. **Make sure that all information entered here is included in the Methods section of the manuscript.**	No official permits or protocol approvals were legally necessary since we did not manipulate individual mice except for checking whether trees were occupied or not by means of live traps. We followed Guidelines of the American Society of Mammalogists for the use of wild mammals in research [49].

Powered by Editorial Manager® and ProduXion Manager® from Aries Systems Corporation

**Format for specific study types**<br><br>**Human Subject Research (involving human participants and/or tissue)**<br>• Give the name of the institutional review board or ethics committee that approved the study<br>• Include the approval number and/or a statement indicating approval of this research<br>• Indicate the form of consent obtained (written/oral) or the reason that consent was not obtained (e.g. the data were analyzed anonymously)<br><br>**Animal Research (involving vertebrate animals, embryos or tissues)**<br>• Provide the name of the Institutional Animal Care and Use Committee (IACUC) or other relevant ethics board that reviewed the study protocol, and indicate whether they approved this research or granted a formal waiver of ethical approval<br>• Include an approval number if one was obtained<br>• If the study involved *non-human primates*, add *additional details* about animal welfare and steps taken to ameliorate suffering<br>• If anesthesia, euthanasia, or any kind of animal sacrifice is part of the study, include briefly which substances and/or methods were applied<br><br>**Field Research**<br><br>Include the following details if this study involves the collection of plant, animal, or other materials from a natural setting:<br>• Field permit number<br>• Name of the institution or relevant body that granted permission	
**Data Availability**<br><br>Authors are required to make all data underlying the findings described fully available, without restriction, and from the time of publication. PLOS allows rare exceptions to address legal and ethical concerns. See the PLOS Data Policy and FAQ for detailed information.	Yes - all data are fully available without restriction

Powered by Editorial Manager® and ProduXion Manager® from Aries Systems Corporation

A Data Availability Statement describing where the data can be found is required at submission. Your answers to this question constitute the Data Availability Statement and **will be published in the article**, if accepted.<br><br>**Important:** Stating 'data available on request from the author' is not sufficient. If your data are only available upon request, select 'No' for the first question and explain your exceptional situation in the text box.<br><br>Do the authors confirm that all data underlying the findings described in their manuscript are fully available without restriction?	
**Describe where the data may be found in full sentences. If you are copying our sample text, replace any instances of XXX with the appropriate details.**<br><br>• If the data are **held or will be held in a public repository**, include URLs, accession numbers or DOIs. If this information will only be available after acceptance, indicate this by ticking the box below. For example: *All XXX files are available from the XXX database (accession number(s) XXX, XXX.).*<br>• If the data are all contained **within the manuscript and/or Supporting Information files**, enter the following: *All relevant data are within the manuscript and its Supporting Information files.*<br>• If neither of these applies but you are able to provide **details of access elsewhere**, with or without limitations, please do so. For example:<br><br>*Data cannot be shared publicly because of [XXX]. Data are available from the XXX Institutional Data Access / Ethics Committee (contact via XXX) for researchers who meet the criteria for access to confidential data.*<br><br>*The data underlying the results presented in the study are available from (include the name of the third party*	All relevant data are within the manuscript and its Supporting Information files.

Powered by Editorial Manager® and ProduXion Manager® from Aries Systems Corporation

and contact information or URL).<br>• This text is appropriate if the data are owned by a third party and authors do not have permission to share the data.<br><br>* typeset	
Additional data availability information:	

Powered by Editorial Manager® and ProduXion Manager® from Aries Systems Corporation

Manuscript

Click here to access/download;Manuscript;MoranLopez_etal_BiolIntegrity_M

Biological integrity of dehesa ecosystems favors acorn dispersal over predation in the mouse-oak mutualism

Teresa Morán-López[1], Jesús Sánchez-Dávila[2], Ignasi Torre[3], Alvaro Navarro-Castilla[4], Isabel Barja[4, 5], Mario Díaz[2]*

1 Laboratorio Ecotono, INIBIOMA-CONICET, Universidad Nacional del Comahue, Black River, Argentina, **2** Department of Biogeography and Global Change (BGC-MNCN-CSIC), National Museum of Natural Sciences, CSIC, c/Serrano 155bis, E-28006 Madrid, Spain, **3** Museu de Ciències Naturals de Granollers (MCNG), E-08402 Granollers, Barcelona, Spain, **4** Departament of Biology, Unit of Zoology, Faculty of Sciences, Universidad Autónoma de Madrid, C/Darwin 2, Campus Universitario de Cantoblanco, 28049 Madrid, Spain, **5** Centro de Investigación en Biodiversidad y Cambio Global (CIBC-UAM) Universidad Autónoma de Madrid, C. Darwin 2, E-28049 Madrid, Spain

** Correspondence: E-mail Mario.Diaz@ccma.csic.es; tel +34 91 782 21 02; fax +34 91 564 08 00*

Running headline: Predation and competition favor dispersal in mouse-oak interaction

Word count: abstract: 272; main text: 4029; 69 references, 2 tables, 3 figures, 5 Supplementary files

Abstract

Scatter-hoarding decisions by rodents are key for the long-term maintenance of oak populations in which avian seed dispersers are absent or inefficient. Decisions are determined by seed value, competition and predation risk. Therefore, they can be influenced by the integrity of the biological system (i.e. trees, rodents, ungulate competitors, and rodent predators). In this work, we manipulate and model the mouse-oak interaction in a Spanish dehesa, an anthropogenic savanna system in which nearby areas can show contrasting levels of mice and ungulate densities, as well as antipredatory cover. First, we conducted a large-scale cafeteria field experiment, where we modified ungulate presence and predation risk, and followed mouse scatter-hoarding decisions under contrasting levels of moonlight and acorn availability. Then, we estimated the net effects of competition and risk by means of a transition probability model that simulated mouse scatter-hoarding decisions. Our experimental and simulation results show that mice are able to adapt their foraging decisions to the environmental context and that such changes affect the probability of acorn dispersal. Under stressful conditions (predation risks and presence of ungulates), mice foraged opportunistically mobilizing large and small seeds. In addition, lack of antipredatory cover around dehesa trees limited the transportation of acorns, but also precluded mice activities outside tree canopies. As a result, post-dispersal predation rates were reduced and large acorns had a higher probability of survival to survive. Overall, our work points out that inter-specific interactions preventing efficient foraging by scatter-hoarders can promote acorn dispersal. Therefore, the maintenance of the full set of producers, seed dispersers and consumers, as well as predators of dispersers in ecosystems, may be key for promoting seed dispersal effectiveness in conditional mutualisms.

Keywords: synzoochory, scatter-hoarding, competition, risk, seed fate

Introduction

Scatter-hoarders are key seed dispersers in temperate and Mediterranean forests dominated by oaks [1,2]. Nut dispersal by scatter-hoarders (synzoochory) is a classical plant-animal conditional mutualism. The outcome of the interaction may be either mutualistic (dispersal) or antagonistic (predation) depending on the proportion of seeds consumed *vs*. cached and not retrieved [3]. The balance between mutualism and antagonism is contingent on intrinsic properties of interaction partners (e.g. propensity of animals to store food) as well as on the ecological setting in which the interaction occurs [2]. As a result, the net effects of synzoochory can be highly dynamic in space and time, making it difficult to predict its outcomes along environmental gradients and ecological timescales [4,5].

Acorn dispersal depends on scatter-hoarder corvids and rodents. Corvids mobilize acorns tens to hundreds of meters providing seed dispersal services to oaks [6], whereas rodents transport acorns locally and a high proportion of them are finally predated [7,8]. Nonetheless, several mouse species (*Apodemus*, *Mus*, *Peromyscus*) can become important acorn dispersers in landscapes where scatter-hoarder corvids are absent [9] or become inefficient [10]. Two main external factors modulate mouse foraging decisions: competition for seeds and predation risks [11–13]. Intraspecific competition and the presence of ungulates tend to encourage seed mobilization [3,14–16]. Especially, when predating seeds *in situ* is more time-consuming than carrying them away. In this context, scatter-hoarding facilitates stockpiling seeds before they are depleted by competitors [12,17]. Risk perception, in turn, depends on factors that affect exposure to predators (e.g. moonlight) as well as direct cues of their presence (e.g. scent) [18–23]. In general, intermediate risks can promote mobilization when mice carry away seeds to manipulate them in safer locations or when handling times of consuming seeds *in situ*

triggers

are too long [12]. However, if lack of cover in the vicinity of trees trigger predation risks, acorn mobilization distances and caching rates can be significantly reduced [13,24]. In general, suboptimal conditions for foraging mice (i.e. competition and predation risk) tend to favor scatter-hoarding over *in situ* predation. In the absence of stress, rodents usually act as efficient seed predators consuming, immediately or soon afterwards, seed crops under the canopy of mother trees [2].

Beyond the environmental conditions of plant-animal encounters, seed size can affect the initial outcomes of the interaction (selected, eaten or cached) as well as post-dispersal processes such as germination and seedling survival. Larger seeds are usually selected and preferentially cached because they provide higher food rewards [7,25–29]. In addition, seed size enhances post-dispersal seedling survival and establishment [30], which is a key component of dispersal effectiveness [31] in scatter-hoarder animals

what's the "sign" of acorn size?

[32]. Nonetheless, the strength and even sign of acorn size effects on mouse foraging decisions are not unequivocal, but context-dependent. Larger acorns are most preferred when food is scarce [33–35], but may be avoided when longer handling times [26] diminish their profitability [36,37] or result in unaffordable predation risks during

roles in

manipulation [11,12]. Therefore, to have a full picture of mouse role on acorn dispersal, we need to account for seed size effects on scatter-hoarding decisions as well as the influence of competition and risk.

In this context, dehesas represent an excellent study system to assess the main factors modulating mouse foraging decisions, and hence, acorn dispersal. They are savanna-like habitats, simpler than natural forests but diverse enough to maintain all key elements influencing the mouse-oak conditional mutualism. In spite of this, dehesas, as well as other man-made systems dominated by scattered trees, suffer from a chronic lack of tree regeneration that compromises its long-term sustainability [38,39]. Depending on the

local intensity of management, nearby areas can have contrasting levels of shrub cover, mice densities and competition with ungulates [18,40]. In addition, the community of predators is simpler than in forest areas, facilitating the experimental manipulation of direct cues of risks [23]. In this work we take advantage of a large-scale experiment of ungulate exclosure in a Mediterranean dehesa to (1) quantify acorn size effects across different stages of the dispersal process (from seed choices to initial fates); and (2) evaluate if size effects are consistent across contrasting scenarios of predation risk and competition (inter- and intraspecific). In addition, we parameterized a transition probability model that assembled all scatter-hoarding decisions by mice to quantify and tease apart the net effect of competition and risk on early stages of the acorn dispersal process. We expected that suboptimal conditions for mice (i.e. competition and risk) would constrain their ability to forage efficiently, thus benefiting acorn dispersal over predation.

Methods

Study area and species

Field work was carried out in the holm oak *Quercus ilex* dehesa woodlands of the Cabañeros National Park (Central Spain, Ciudad Real province, 39°24’ N, 38°35 W). Dehesas are savanna-like man-made habitats resulting from shrub removal and tree thinning and pruning to enhance herb growth for livestock [41]. The studied dehesas were opened from the original Mediterranean forests in the late 1950s. Currently they have no livestock but wild ungulate populations of red deer *Cervus elaphus* and wild boars *Sus scrofa*. Deer densities were around 0.14 ind./ha [42] and boars are abundant but at unknown densities [43]. Acorns fall from trees from mid-October to late November [9].

The study area covers around 780 ha, with two ungulate exclosures (site 1 and 2) made with wire fences 2 m tall and 32 cm x 16 cm mesh of 150 ha and 4.65 ha separated from each other by 1500 m. The exclosures prevent the entrance of ungulates but not of mesocarnivores (mainly common genets *Genetta genetta* and red foxes *Vulpes vulpes*; pers. obs. based on scat searches) and raptors. In the study area, average tree density was 20.4 trees ha-1, although site 1 had higher mean density than site 2 (30.0±2.6 and 30.1±3.4 inside and outside ungulate exclosure in site 1, and 7.4±0.4 and 7.4±0.3 in site 2; mean±SE). Shrub cover was <1%, as measured on aerial photographs and vegetation surveys both under canopies and outside them [23]. Mean tree canopy radius was 4.2±0.7 m (range 2.5-7.1 m). The Algerian mouse (*Mus spretus*) is the most abundant scatter-hoarding rodent in the area [44], and it is a common prey of genets and other generalist predators [45,46].

Experimental design

Tree occupancy by mice was established by means of live trapping using Sherman traps (23 × 7.5 × 9 cm; Sherman Co., Tallahassee, USA) baited with canned tuna in olive oil mixed with flour and a piece of apple. Water-repellent cotton was provided to prevent the cooling of the individual captured overnight. Traps were set during two consecutive days during the new moon of January 2012. High capture probability of *M. spretus*

) "allowed to consider" is awkward, suggest rewording

(detectability: 0.88±0.03SE; [47] allowed to consider false negatives in occupancy unlikely. Among trees known to be occupied by Algerian mice, we randomly selected ten trees inside and ten outside in each of the two exclosures (40 focal trees in total).

We paired focal trees according to their proximity and we randomly assigned a predator scent treatment to one of them. Predator scent treatment consisted of placing fresh genet feces (10 g) mixed with distilled water close to a corner of the cages where acorns were

placed [23]. Genets are generalist predators whose presence and scats are known to influence rodent behavior [20,22,48]. Fresh feces were collected from two captive common genets housed in the Cañada Real Open Center (Madrid, Spain).

Fresh acorns were collected from holm oaks growing near the study area in October 2011 and stored dry in a cooler (4 ºC) until use. Sound acorns, with no marks of insect damage [45], were weighed with a digital balance to the nearest 0.01 g. Groups of 15 acorns in three categories were randomly selected (5 each, large, >10 g; medium-sized, 5-10 g, and small, 1-5 g). Acorns were placed under the canopy of each focal tree inside a 50 cm × 50 cm x 15 cm galvanized-steel cage to prevent acorn consumption by birds or ungulates [44]. Cages were located 1.2 m on average (range 0.3-2.7 m) from focal tree trunks. A metal wire (ø 0.6 mm, 0.5 m length) with a numbered plastic tag was
acorns
attached to each acorn [49]. After removing any naturally-present acorn within the
3 row x 5 column
cages, we randomly placed acorns in the intersection of a 3 rows x 5 columns grid. To track mouse choices, acorn size for each position was noted. Acorns were left exposed to mice for three consecutive nights, then removed. Mobilized acorns were located by
for
looking at the plastic tags during the following days (24 and 72 hours). We tracked the status of acorns that were mobilized and not predated throughout the experiment. However, outside exclosures we were often unable to tease apart the disappearance of
How large was the search area? Was there a standardized search distance from each focal tree?
mobilized acorns due to predation by rodents from that of ungulates. Therefore, this long-term monitoring was not included in the analyses. To account for changes in night brightness and acorn availability [21,50], the cafeteria experiment was repeated four times during the full-moon and new-moon periods of November 2011 and February 2012. No official permits or protocol approvals were legally necessary , since we did not manipulate individual mice except for checking whether trees were occupied or not by means of live traps. We followed Guidelines of the American Society of Mammalogists

for the use of wild mammals in research [51]. We performed all manipulations with disposable latex gloves, to avoid effects of human odor on rodent behavior [52].

Mouse foraging behavior

A video-camera OmniVision CMOS 380 LTV (OmniVision, Santa Clara, USA) (3.6 mm lens) monitored mouse foraging activity within each cage. Cameras were set on 1.5 m tall tripods located 2.5 m from each cage, powered by car batteries (70 Ah, lead acid) connected to a solar panel (ono-silicon erial P_20; 20 w). Video-cameras were connected to ELRO recorders with dvr32cards (ELRO, Amsterdam, Netherlands) and took continuous recording for three consecutive days autonomously (recorded in quality at 5 frames s^{-1}). Events with rodent activity, from the entry of the individual into the cage up to the exit from it, were located and separated using Boilsoft Video Splitter software (https://www.boilsoft.com/videosplitter/) [48]. Within each event we noted which acorn was manipulated and whether it was removed outside the cage. For removed acorns we measured mobilization distance (cm) and noted its status (predated or not after transportation).

What was categorized as "predated"? Were acorn fragments observed? A tag left behind?

Data analysis

To assess acorn choice by rodents, we fitted a hierarchical multinomial model. Our response variable was acorn selection (yes/no). Our explanatory variables were: acorn size (g), moon phase (new/full), month (February, November), ungulate presence (yes/no), predator scent (yes/no), acorn availability in the cage (g) and the two-way interactions between size and environmental effects. Local acorn availability was

delete ","

measured as total acorn mass in the cage during the event. Both, acorn size and availability were scaled previous to the analyses (mean = 0, sd = 1) so that we could

compare the magnitude of covariate effects. Focal tree was introduced as a random factor in the intercept term to account for repeated sampling during the experiment. To evaluate the effects of acorn size on the probability of removal, we used a hierarchical logistic model. Our response variable was whether a selected acorn was mobilized outside the cage or not (yes/no). Our explanatory variables and random effects were the same as in the multinomial model.

Subsequently, we analyzed the effect of acorn size and environmental covariates (and their two-way interaction) on seed dispersal. Our response variables were mobilization distances (cm, log-transformed) and deposition status (viable or predated). We used a hierarchical Gaussian model in the former case, and a hierarchical logistic model in the latter. Our explanatory variables and random effects were the same as in the previous models. In all four models (selection, removal, mobilization distance and initial fate) we used uninformative priors (Supplementary File 1). All analyses were performed employing a Bayesian approach with JAGS 3.4.0 [53]. We checked for convergence for all model parameters (Rhat < 1.1) and that the effective sample size of posterior distributions was high (>800). We estimated the mean and credible interval of posterior distributions. Also, we calculated the proportion of the posterior distribution with the same sign of the mean (f) and evaluated the predictive power of our models by means of posterior predictive checks (Supplementary Files 1 and 2).

Simulating scatter-hoarding decisions

To estimate the joint effect of seed size, competition and risk on acorn dispersal we designed a probability transition model in which simulated mice adapted their foraging behavior to the environmental context (Supplementary File 3). Before model run, we parameterized mouse scatter-hoarding decisions (from selection to initial fate) following

the same scheme of regressions explained in the previous section. Nonetheless, here we only used data from November, the period of peak acorn falling in our study system. Consequently, we did not include month as a covariate. For each behavioral submodel (selection, removal and initial fate), we obtained posterior distributions of parameters by running 50000 iterations in three chains (in all cases Rhat< 1.1, and Neff> 1000).

Model setup mimics our experimental design, 20 trees outside and 20 inside exclosures paired according to a predator scent treatment (presence *vs* absence). Simulations begin under new moon conditions with focal trees offering 15 acorns of large, medium and small sizes (5 each). Acorn size is sampled from empirical distributions of these size categories. In each focal tree, the number of foraging events is drawn from a Poison distribution with mean equal to the average number of events observed in the corresponding moon phase. During each foraging event, simulated mice decide which acorn to handle and whether to remove it or not. If removed, mice decide whether to predate it or not after mobilization, and acorn availability in the cage is updated. Once all foraging events (of all trees) are simulated, acorn dispersal is modelled under full moon conditions (Supplementary File 3, Fig. S1).

For each model run we sampled parameter of behavioral submodels (selection, removal and deposition) from posterior distributions fitted to data. Thus, in our simulations, mice adapted their decisions to acorn size and availability (in the experimental cage), characteristics of the focal tree (i.e. ungulate and predator scent presence), and the moon phase in which the foraging event occurs (new or full moon). After each model run (dispersal under new and full moon conditions), the program tracked the size and status of handled acorns and the environmental covariates in which the foraging event occurred. We run the model 1000 times and plotted deposition rates of viable acorns and their size with respect to the moon phase and tree characteristics (predator scent and

ungulate presence). See Supplementary File 3 for detailed model specifications and Fig. S1 for a summary of the process overview.

Results

Before setting the cafeteria experiments in November, we removed from cages 53.3 acorns/m^2 on average (range: 0-104). No acorn was found in February. We monitored 2280 acorns under 38 focal trees. We detected mouse activity in 18 and 26 trees in the new and full moon of November, and in 26 and 24 trees in the new and full moon of February, respectively. Mice manipulated 1677 acorns. Out of them, 267 were mobilized outside cages and 211 (79%) were relocated.

acorns were

How many were cached versus predated? We the dispersed acorns all buried or were some found on the surface? Were they all found under focal tree canopies? It would be nice to have that information here.

Foraging decisions in the focal tree: selection and removal

In general, mice selected larger acorns, but the positive effect of size was modulated by environmental conditions. Size-driven selection preferentially occurred in the absence of competition with ungulates (Fig. 1A) and predator scent (Fig. 1B). In addition, mouse selectivity was enhanced under low local acorn availability (Table 1, selection). Among selected acorns, mice preferentially removed smaller ones. Such selective behavior occurred when risks were low due to reduced night brightness (new moon, Fig. 1C) or lack of predator scent (Fig. 1D), as well as when ungulates were absent (Table 2). Acorn availability at local and landscape scales did not modify size effects, although they changed mobilization rates. Rates were lower during the acorn fall peak (13% in November *vs* 24 % in February), whereas acorn availability in cages enhanced removal (Table 2, removal).

Figure 1 doesn't have "A, B, C, D" designations, so they should be added

Foraging decisions during dispersal: mobilization distances and predation

shorter distances

Mice mobilized acorns closer under new moon conditions (Fig. 2A) and when ungulates were present (Fig. 2B). During lean periods (February) mobilization distances and post-dispersal predation increased (Table 2, Month). In addition, larger acorns were preferentially consumed (Fig. 2C), though the presence of ungulates and full moon conditions attenuated this negative effect (Fig. 2D, Table 2).

Transition probability model for acorn dispersal

Under optimal conditions (new moon, no predator scent or ungulates), post-dispersal predation rates were higher (Fig. 3A) and simulated mice preferentially consumed large acorns (i.e. viable acorns -blue bars- were smaller, Fig. 3B-D). However, predation risks and ungulate presence precluded acorn consumption after mobilization and attenuated selection. As a result, the proportion and size of viable acorns increased (Fig. 3A and B-D).

Did the proportion and size of viable acorns that REMAINED increased? This sentence seems incomplete.

Discussion

Overall, our work shows that mice are able to adapt their foraging decisions to perceived predation risks and competition for seeds. Also, that such behavioral adjustments affect the fate of acorns at initial stages of the dispersal process. When relaxed, mice preferentially consumed large acorns and removed small ones. Furthermore, mobilized seeds were more likely to be predated. In contrast, under stressful conditions (increased predation risk and competition) mice foraged opportunistically and reduced their activity outside tree canopies. As a result, predation rates after mobilization decreased, and larger acorns had a higher probability to survive, at least in the short term. This bolsters the idea that interactions with third-party players

"...seeds, and that such..."

of survival

can modify the qualitative component of dispersal effectiveness of scatter-hoarding rodents [12,15,54].

As expected, larger and more valuable acorns were preferentially handled by mice, which adapted this behavior to the environmental context [12]. In line with previous work, mice foraged opportunistically in trees with predator scent, probably because they

"...at the expense of..."

devoted more time to vigilant behaviors [15,48] at expenses of acorn discrimination [21]. In contrast, acorn availability effects did not follow the expectations of increased selectivity in scenarios of food depletion [28,54,55]. Seed size effects were similar between acorn fall peaks and lean periods. Furthermore, mice foraged randomly when ungulates were present, while they selected larger seeds within exclosures. We expected that competition for acorns would promote a more selective behavior to ensure the acquisition of more valuable food items [28]. However, we found the opposite probably due to some particularities of our system. Dehesas are characterized by high acorn production and scarce shrub cover (<1%) around trees [41,56,57]. Under such circumstances, the effects of increased predation risks outside tree canopies can outweigh those of competition leading to a rapid and random harvesting of seeds [21]. In contrast, within ungulate exclosures, reduced grazing and soil compaction has promoted taller resprouts under canopies and increased cover of herbs and tussocks around trees [58]. As a result, in the absence of ungulates mice can forage under shelter, devoting less time to vigilant behaviors [48]. The fact that in the presence of ungulates

indicates

mice foraged opportunistically, point out that in dehesas predation risks modulate acorn selection. Also, changes in vegetation cover due to ungulate presence can strongly affect mouse scatter-hoarding decisions [17].

Larger acorns tend to be carried away, mobilized farther and preferentially cached in forest habitats [7,27,59,60]. However, in our study larger acorns had a higher

probability of being predated (*in situ* and after transportation) and seed size did not affect mobilization distances. Again, these results highlight that environmental conditions in dehesas are particularly harsh for rodents. In general, rodents preferentially mobilize small seeds when the costs of carrying large ones result are unaffordable [12]. In the presence of ungulates, low antipredatory cover and high trampling risks may have triggered transportation costs [13,61], deterring mice from carrying large seeds away. Seed size effects were not fixed, but depended on direct and indirect cues of risk. Preferential removal of small seeds only occurred in trees with no predator scent or under new moon conditions, reflecting that only when risks are mild reduced mice can take the time to select among the seeds available [15,21].

Regarding post-dispersal survival, we expected higher predation when acorns were deposited close to tree canopies [13,24], but this relationship blurred in our system. Outside ungulate exclosures, larger acorns had a higher probability of escaping predation in spite of being mobilized nearby source trees. In dehesas, the pervasiveness of open land cover forces mice to concentrate their activities beneath canopies [13,23,58], and decreases the likelihood that mobilized acorns are encountered and consumed [62]. Accordingly, in our simulations, suboptimal conditions (due to increased risks or ungulate presence) discouraged mice from consuming seeds after mobilization. Consequently, predation rates were reduced and larger acorns had a higher probability to survive. of survival In Mediterranean systems, seedlings from larger acorns are more resistant to summer drought [30,63], which represents the main recruitment bottleneck for oak regeneration [39,64]. Therefore, higher survival rates of large acorns suggest that intermediate levels of stress can enhance the qualitative component of seed dispersal effectiveness by mice (as suggested by [65]).

This work builds on previous research analyzing the effects of competition and risk on mouse foraging behavior in dehesas [48]. Here, by accounting for all stages of scatter-hoarding decisions (from initial manipulation to consumption after mobilization [60]), and including the entire acorn fall season [27] as well as contrasting moon light conditions [21], we obtained a more in-depth understanding of the main drivers of dispersal effectiveness. Moreover, our transition probability model allowed us to assemble all stages of the scatter-hoarding process, and hence, to estimate the net effects of competition and predation risks on initial seed fate. Nonetheless, long-term survival of mobilized acorns was not assessed, and hence, our results do not allow us to estimate actual seed dispersal effectiveness [31]. Throughout the autumn, retrieval rates of cached acorns can be high (less than 5% of offered acorns survive to the next spring [7,66]). In addition, lack of shrub cover in dehesas can limit recruitment due to seedling dry out during summer [9,67]. Therefore, it remains an open question whether cache recovery or seedling death can outweigh the positive effects of ungulate presence and risk on initial stages of acorn dispersal.

delete "Here,"

While the study provided novel insights regarding the impacts of grazing and predation risk, it didn't report important components of dispersal effectiveness.

only all stages according to [60], suggest replacing with "many"

seedlings drying

Quantifying dispersal effectiveness in animals that mobilize a large number of seeds, but predate a high proportion of them is a challenging task. High mobilization rates can result in an ecologically relevant number of seedlings in spite of intensive predation [2,7]. However, to obtain reliable estimates of post-dispersal survival, we need to track the fate of a large (and often unaffordable) number of seeds. For instance, between 1.3 and 3% of offered acorns survive to the next spring [7,66]. Thus, to estimate the effects of environmental factors and seed size on long-term cache survival (sample size > 200) between 6700 and 15000 acorns should have been tracked. A way to circumvent this problem, is directly sowing acorns and monitoring artificial caches. Such approach does not allow to evaluate foraging decisions made by cache owners, but informs about post-

delete "allow to"

dispersal survival from pilferers and ungulates, as well as the probability of emergence and early establishment [67,68]. Once this information is available, it could be easily included in our transition probability model (following [69]), allowing to evaluate if changes in mouse foraging decisions have an imprint on seedling recruitment.

Concluding remarks

Our mechanistic approach provides new insights about the joint effect of habitat structure, competition and risk on the foraging behavior of mice and its potential consequences on acorn dispersal. In the presence of ungulates and when predation risks were high, mice acted as opportunistic foragers and concentrated their activities beneath tree canopies. As a result, predation rates decreased and larger acorns had a higher probability to survive (at least in the short term). These results suggest that inefficient foraging by rodents can promote acorn dispersal. Also, they highlight the importance of competition and risk on the spatial and temporal dynamism of synzoochorous interactions [2]. Finally, though future work is needed to estimate long-term cache survival and seedling establishment, our results support the view that biological integrity (presence of the full set of producers, consumers, dispersers and predators) can facilitate seed dispersal effectiveness in synzoochorous mutualisms [2,54]. This may be particularly important in man-made habitats like dehesas, which depend on conditional mutualisms to ensure their long-term sustainability [39,57]

Supporting information

S1. Structure of models and priors

S2. Posterior predictive checks

S3. Specifications of transition probability model for acorn dispersal

S4. Code for the transition probability model

S5. Databases

Acknowledgements

D. López, M. Fernández and C. L. Alonso helped during fieldwork. D. López, B. Ramos and M. de Pablo pre-processed the video recordings, and D. Gallego, D. Valero, A. Velasco, C. J. González and E. Sánchez visualized the recordings noting seed choices. Authorities of the Cabañeros National Park provided the official permissions to carry out field experiments. J. España provided common genet scats.

Author contributions

MD conceived and executed the field experiment with the aid of IT, IB, TM-L and AN-C. JS-D compiled the data and performed preliminary analyses and drafts. TM-L executed the final data analyses and proposed the final main focus of the paper. TM-L and MD wrote the final version of the paper on former versions drafted by JS-D and contributed by all authors.

References

1. Vander Wall SB. The evolutionary ecology of nut dispersal. Bot Rev. 2001;67: 74–117.

2. Gómez JM, Schupp EW, Jordano P. Synzoochory: the ecological and evolutionary relevance of a dual interaction. Biol Rev. 2019;94: 874–902.

3. Theimer T. Rodent scatterhoaders as conditional mutualists. In: Forget PM, Lambert JE HP, editor. Seed Fate: Predation, dispersal, and seedling establishment. Wallingford: CAB International; 2005. pp. 283–295.

4. Bronstein JL. Conditional outcomes in mutualistic interactions. Trends Ecol Evol. 1994;9: 214–217.

5. Sawaya GM, Goldberg AS, Steele MA, Dalgleish HJ. Environmental variation shifts the relationship between trees and scatterhoarders along the continuum from mutualism to antagonism. Integr Zool. 2018;13: 319–330.

6. Pesendorfer MB, Sillett TS, Koenig WD, Morrison SA. Scatter-hoarding corvids as seed dispersers for oaks and pines: a review of a widely distributed mutualism and its utility to habitat restoration. Condor Ornithol Appl. 2016;118: 215–237.

7. Gómez JM, Puerta-Piñero C, Schupp EW. Effectiveness of rodents as local seed dispersers of Holm oaks. Oecologia. 2008;155: 529–537.

8. Kellner KF, Lichti NI, Swihart RK. Midstory removal reduces effectiveness of oak (Quercus) acorn dispersal by small mammals in the Central Hardwood Forest region. For Ecol Manage. 2016;375: 182–190.

9. Pulido F, García E, Obrador JJ, Moreno G. Multiple pathways for tree regeneration in anthropogenic savannas: incorporating biotic and abiotic drivers into management schemes. J Appl Ecol. 2010;47: 1272–1281.

10. Morán-López T, Alonso CL, Díaz M. Landscape effects on jay foraging behavior decrease acorn dispersal services in dehesas. Acta Oecologica. 2015;69. doi:10.1016/j.actao.2015.07.006

11. Steele MA, Contreras TA, Hadj-Chikh LZ, Agosta SJ, Smallwood PD, Tomlinson CN. Do scatter hoarders trade off increased predation risks for lower rates of cache pilferage? Behav Ecol. 2014;25: 206–215.

12. Lichti NI, Steele MA, Swihart RK. Seed fate and decision- making processes in scatter- hoarding rodents. Biol Rev. 2017;92: 474–504.

13. Morán-López T, Wiegand T, Morales JM, Valladares F, Díaz M. Predicting forest management effects on oak–rodent mutualisms. Oikos. 2016;125. doi:10.1111/oik.02884

14. Puerta- Piñero C, María Gómez J, Schupp EW. Spatial patterns of acorn dispersal by rodents: do acorn crop size and ungulate presence matter? Oikos. 2010;119: 179–187.

15. Sunyer P, Munoz A, Bonal R, Espelta JM. The ecology of seed dispersal by small rodents: a role for predator and conspecific scents. Funct Ecol. 2013;27: 1313–1321.

16. Morán- López T, Fernández M, Alonso CL, Flores- Rentería D, Valladares F, Díaz M. Effects of forest fragmentation on the oak–rodent mutualism. Oikos. 2015;124: 1482–1491.

17. Zhang H, Wang Y, Zhang Z. Domestic goat grazing disturbance enhances tree seed removal and caching by small rodents in a warm-temperate deciduous forest in China. Wildl Res. 2009;36: 610–616.

18. Díaz M, Gonzalez E, Munoz-Pulido R, Naveso MA. Effects of food abundance and habitat structure on seed-eating rodents in Spain wintering in man-made habitats. Zeitschrift für Säugetierkd. 1993;58: 302–311.

19. Orrock JL, Danielson BJ, Brinkerhoff RJ. Rodent foraging is affected by indirect, but not by direct, cues of predation risk. Behav Ecol. 2004;15: 433–437.

20. Díaz M, Torre I, Peris A, Tena L. Foraging behavior of wood mice as related to presence and activity of genets. J Mammal. 2005;86: 1178–1185.

21. Perea R, González R, San Miguel A, Gil L. Moonlight and shelter cause

differential seed selection and removal by rodents. Anim Behav. 2011;82: 717–723.

22. Navarro- Castilla Á, Barja I. Antipredatory response and food intake in wood mice (Apodemus sylvaticus) under simulated predation risk by resident and novel carnivorous predators. Ethology. 2014;120: 90–98.

23. Navarro-Castilla Á, Barja I, Díaz M. Foraging, feeding, and physiological stress responses of wild wood mice to increased illumination and common genet cues. Curr Zool. 2018;64: 409–417.

24. Morán-López T, Fernández M, Alonso CL, Flores-Rentería D, Valladares F, Díaz M. Effects of forest fragmentation on the oak-rodent mutualism. Oikos. 2015;124. doi:10.1111/oik.02061

25. Pons J, Pausas JG. Rodent acorn selection in a Mediterranean oak landscape. Ecol Res. 2007;22: 535–541.

26. Muñoz A, Bonal R. Are you strong enough to carry that seed? Seed size/body size ratios influence seed choices by rodents. Anim Behav. 2008;76: 709–715.

27. Sunyer P, Espelta JM, Bonal R, Muñoz A. Seeding phenology influences wood mouse seed choices: the overlooked role of timing in the foraging decisions by seed-dispersing rodents. Behav Ecol Sociobiol. 2014;68: 1205–1213.

28. T. M-L, Valladares F, Tiribelli F, Pérez-Sepúlveda JE, Traveset A, Diaz M. Fragmentation modifies seed trait effects on scatter-hoardersâ foraging decisions. Plant Ecol. 2018.

29. Pérez- Ramos IM, Urbieta IR, Maranón T, Zavala MA, Kobe RK. Seed removal in two coexisting oak species: ecological consequences of seed size, plant cover and seed- drop timing. Oikos. 2008;117: 1386–1396.

30. Gómez JM. Bigger is not always better: conflicting selective pressures on seed size in Quercus ilex. Evolution (N Y). 2004;58: 71–80.

31. Schupp EW, Jordano P, Gomez JM. Seed dispersal effectiveness revisited: a conceptual review. New Phytol. 2010;188: 333–353. doi:10.1111/j.1469-8137.2010.03402.x

32. Zwolak R, Crone EE. Quantifying the outcome of plant–granivore interactions. Oikos. 2012;121: 20–27.

33. Li H, Zhang Z. Effects of mast seeding and rodent abundance on seed predation and dispersal by rodents in Prunus armeniaca (Rosaceae). For Ecol Manage. 2007;242: 511–517.

34. Wang H, Cao G, Wang L, Yang Y, Zhang Z, Duan Y. Evaluation of pollinator effectiveness based on pollen deposition and seed production in a gynodieocious alpine plant, Cyananthus delavayi. Ecol Evol. 2017;7: 8156–8160.

35. Vander Wall SB. Masting in animal- dispersed pines facilitates seed dispersal. Ecology. 2002;83: 3508–3516.

36. Pulliam HR. Foraging efficiency, resource partitioning, and the coexistence of sparrow species. Ecology. 1985;66: 1829–1836.

37. Díaz M. Variability in seed size selection by granivorous passerines: effects of bird size, bird size variability, and ecological plasticity. Oecologia. 1994;99: 1–6.

38. Campos P, Huntsinger L, Oviedo JL, Starrs PF, Díaz M, Standiford RB, et al. Mediterranean oak woodland working landscapes. Landsc Ser. 2013;16.

39. Diaz M, Sánchez-Mejía T, Morán-López T. Long-term tree regeneration of fragmented agroforestry systems under varying climatic conditions. Habitat Modif Landsc Fragm Agric Ecosyst Implic Biodivers Landsc Multi-Functionality. 2022.

40. Munoz A, Bonal R, Díaz M. Ungulates, rodents, shrubs: interactions in a diverse Mediterranean ecosystem. Basic Appl Ecol. 2009;10: 151–160.

41. Pulido FJ, Díaz M, de Trucios SJH. Size structure and regeneration of Spanish

holm oak Quercus ilex forests and dehesas: effects of agroforestry use on their long-term sustainability. For Ecol Manage. 2001;146: 1–13.

42. Jiménez J. El ciervo (Cervus elaphus) en la zona oriental del Parque Nacional de Cabaneros. Madrid Org Autónomo Parques Nac. 2004.

43. Perea R, Gil L. Tree regeneration under high levels of wild ungulates: The use of chemically vs. physically-defended shrubs. For Ecol Manage. 2014;312: 47–54.

44. Díaz M, Alonso CL, Beamonte E, Fernández M, Smit C. Development of a long-term monitoring protocol of keystone organisms for the functioning of Mediterranean forests [in Spanish]. In: Ramírez L AB, editor. Proyectos de investigación en parques nacionales 2007-2010. Madrid: , Organismo Autónomo Parques Nacionales; 2011. pp. 47–75.

45. Palomo LJ, Justo ER, Vargas JM. Mus spretus (Rodentia: muridae). Mamm species. 2009; 1–10.

46. Torre I, Arrizabalaga A, Freixas L, Ribas A, Flaquer C, Díaz M. Using scats of a generalist carnivore as a tool to monitor small mammal communities in Mediterranean habitats. Basic Appl Ecol. 2013;14: 155–164.

47. Torre I, Raspall A, Arrizabalaga A, Díaz M. SEMICE: an unbiased and powerful monitoring protocol for small mammals in the Mediterranean Region. Mamm Biol. 2018;88: 161–167.

48. Gallego D, Morán-López T, Torre I, Navarro-Castilla Á, Barja I, Díaz M. Context dependence of acorn handling by the Algerian mouse (Mus spretus). Acta Oecologica. 2017;84. doi:10.1016/j.actao.2017.07.002

49. Xiao Z, Jansen PA, Zhang Z. Using seed-tagging methods for assessing post-dispersal seed fate in rodent-dispersed trees. For Ecol Manage. 2006;223: 18–23.

50. Diaz M. Rodent seed predation in cereal crop areas of central Spain: effects of

physiognomy, food availability, and predation risk. Ecography (Cop). 1992;15: 77–85.

51. Sikes RS, Gannon WL. Guidelines of the American Society of Mammalogists for the use of wild mammals in research. J Mammal. 2011;92: 235–253.

52. Wenny DG. Effects of human handling of seeds on seed removal by rodents. Am Midl Nat. 2002; 404–408.

53. Plummer M. JAGS: A program for analysis of Bayesian graphical models using Gibbs sampling. Proceedings of the 3rd international workshop on distributed statistical computing. Vienna, Austria.; 2003. pp. 1–10.

54. Schupp EW, Zwolak R, Jones LR, Snell RS, Beckman NG, Aslan C, et al. Intrinsic and extrinsic drivers of intraspecific variation in seed dispersal are diverse and pervasive. AoB Plants. 2019;11. doi:10.1093/aobpla/plz067

55. Stephens DW, Krebs JR. Foraging theory. Princeton university press; 2019.

56. Koenig WD, Díaz M, Pulido F, Alejano R, Beamonte E, Knops JMH. Acorn production patterns. Mediterranean oak woodland working landscapes. Springer; 2013. pp. 181–209.

57. Díaz M. Tree scattering and long-term persistence of dehesas: patterns and processes. Ecosistemas. 2014;23: 5–12.

58. Navarro-Castilla Á, Díaz Esteban M, Barja I. Does ungulate disturbance mediate behavioural and physiological stress responses in Algerian mice (Mus spretus)? A wild exclosure experiment. 2017.

59. Wang B, Chen J. Seed size, more than nutrient or tannin content, affects seed caching behavior of a common genus of Old World rodents. Ecology. 2009;90: 3023–3032.

60. Wang B, Ye C, Cannon CH, Chen J. Dissecting the decision making process of scatter- hoarding rodents. Oikos. 2013;122: 1027–1034.

61. Muñoz A, Bonal R. Rodents change acorn dispersal behaviour in response to ungulate presence. Oikos. 2007;116: 1631–1638.

62. Muñoz A, Bonal R. Linking seed dispersal to cache protection strategies. J Ecol. 2011;99: 1016–1025.

63. Bonito A, Varone L, Gratani L. Relationship between acorn size and seedling morphological and physiological traits of Quercus ilex L. from different climates. Photosynthetica. 2011;49: 75–86.

64. Pulido FJ, Díaz M. Regeneration of a Mediterranean oak: a whole-cycle approach. Ecoscience. 2005;12: 92–102.

65. Feldman M, Ferrandiz-Rovira M, Espelta JM, Muñoz A. Evidence of high individual variability in seed management by scatter-hoarding rodents: does ‘personality’matter? Anim Behav. 2019;150: 167–174.

66. Perea R, San Miguel A, Gil L. Acorn dispersal by rodents: the importance of re-dispersal and distance to shelter. Basic Appl Ecol. 2011;12: 432–439.

67. Smit C, Díaz M, Jansen P. Establishment limitation of holm oak (Quercus ilex subsp. ballota (Desf.) Samp.) in a Mediterranean savanna–forest ecosystem. Ann For Sci. 2009;66: 1–7.

68. Gómez JM, Garcıa D, Zamora R. Impact of vertebrate acorn-and seedling-predators on a Mediterranean Quercus pyrenaica forest. For Ecol Manage. 2003;180: 125–134.

69. González-Castro A, Calviño-Cancela M, Nogales M. Comparing seed dispersal effectiveness by frugivores at the community level. Ecology. 2015;96: 808–818. doi:https://doi.org/10.1890/14-0655.1

Table 1. Summary table of the effects of size, moonlight, month, ungulate presence, predator scent and local acorn availability (and their interactions with size) on the probability of acorn selection and removal. A total of 1677 foraging events were analyzed.

Process	Fixed effect	Mean	HPD1	f	
**Acorn selection**	**Size**	**0.19**	**[0.09, 0.29]**	**1.00**	**
	Moon (Full)	0.03	[-6.22, 6.35]	0.50	
	Month (February)	-0.03	[-6.12, 6.06]	0.50	
	Ungulate (Yes)	-0.05	[-6.27, 6.16]	0.51	
	Scent (Yes)	0.01	[-6.16, 6.28]	0.50	
	Availability	-0.02	[-6.32, 6.19]	0.50	
	Size*Moon	0.07	[-0.03, 0.17]	0.93	
	Size*Month	-0.06	[-0.16, 0.04]	0.88	
	**Size*Ungulates**	**-0.13**	**[-0.23, -0.03]**	**0.99**	**
	**Size*Scent**	**-0.08**	**[-0.18, 0.01]**	**0.96**	*
	**Size*Availabiltiy**	**-0.04**	**[-0.09, 0.01]**	**0.95**	*
**Acorn removal**	**Size**	**-0.50**	**[-0.94, -0.07]**	**0.99**	**
	Moon (Full)	0.07	[-0.27, 0.39]	0.65	
	**Month (February)**	**0.77**	**[0.43, 1.11]**	**1.00**	**
	Ungulate (Yes)	-0.22	[-0.96, 0.47]	0.73	
	Scent (Yes)	0.20	[-0.53, 0.93]	0.72	
	**Availability**	**0.29**	**[0.12, 0.46]**	**1.00**	**
	**Size*Moon**	**0.29**	**[-0.02, 0.60]**	**0.96**	*
	Size*Month	-0.09	[-0.41, 0.22]	0.71	
	**Size*Ungulates**	**0.24**	**[-0.07, 0.55]**	**0.94**	•
	**Size*Scent**	**0.30**	**[0.00, 0.59]**	**0.98**	*
	Size*Availabiltiy	0.10	[-0.07, 0.26]	0.88	

Mean of posterior distribution, highest posterior density interval (HPD) and percentage of the posterior distribution with the same sign as the mean (f) are shown. Effects with f ≥ 0.95 are in bold. • depicts f ∈ [0.90, 0.95)

Table 2. Summary table of the effects of size, moonlight, month, ungulate presence, predator scent and local acorn availability (and their interactions with size) on acorn mobilization distances and the probability that it is deposited in a viable status (vs predated). A total of 211 acorns (mobilized outside cages and retrieved) were analyzed.

cached intact

Process	Fixed effect	Mean	HPD	F	
Mobilization distance	Size	0.16	[-0.51, 0.83]	0.68	
	**Moon (Full)**	**-0.67**	**[-1.27, -0.06]**	**0.98**	*
	**Month (February)**	**0.54**	**[-0.14, 1.2]**	**0.94**	·
	**Ungulate (Yes)**	**-0.75**	**[-1.59, 0.14]**	**0.95**	*
	Scent (Yes)	0.09	[-0.73, 0.98]	0.57	
	Availability	-0.01	[-0.31, 0.29]	0.52	
	Size*Moon	-0.07	[-0.66, 0.49]	0.60	
	Size*Month	-0.33	[-0.94, 0.28]	0.86	
	Size*Ungulates	0.18	[-0.44, 0.81]	0.71	
	Size*Scent	0.22	[-0.36, 0.79]	0.78	
	Size*Availabiltiy	-0.16	[-0.49, 0.17]	0.83	
Viability after deposition	**Size**	**-1.20**	**[-2.15, -0.33]**	**1**	*
	Moon (Full)	0.42	[-0.38. 1.22]	0.85	
	**Month (February)**	**-1.58**	**[-2.46, -0.75]**	**1**	*
	**Ungulate (Yes)**	**0.67**	**[-0.32, 1.69]**	**0.91**	·
	Scent (Yes)	-0.16	[-1.14, 0.80]	0.63	
	**Availability**	**0.52**	**[0.10, 0.97]**	**0.99**	*
	**Size*Moon**	**0.66**	**[-0.12, 1.46]**	**0.95**	*
	Size*Month	0.49	[-0.33, 1.34]	0.88	
	**Size*Ungulates**	**0.59**	**[-0.22, 1.40]**	**0.92**	·
	Size*Scent	0.21	[-0.52, 0.94]	0.72	
	Size*Availabiltiy	-0.24	[-0.73, 0.25]	0.84	

Mean of posterior distribution, highest posterior density interval (HPD) and percentage of the posterior distribution with the same sign as the mean (f) are shown. Effects with f ≥ 0.95 are in bold. • depicts f $\in [0.90, 0.95)$

Figure legends

Fig. 1. Mouse foraging decisions during acorn selection and removal (upper and lower panels, respectively). Size of acorns (selected or not) in the presence or absence of (A) ungulates and (B) predator scent. Size of acorns (removed or not) (C) under new or full moon conditions and (D) in the presence or absence of predator scent. Point colors depict whether the acorn was selected and or removed (yes, blue) or not (no, orange). In all cases acorn size is expressed in grams. Points represent mean values, bars standard errors (N=1677 foraging events).

Fig. 2. Mouse foraging decisions during transportation and after deposition (upper and lower panels, respectively). Mobilization distances of removed acorns under (A) new and full moon conditions and (B) in the absence and presence of ungulates. (C) Seed size effects on the probability of predation after deposition (black line represents mean effects and shaded area 0.95 credible intervals). (D) Proportion of acorns escaping predation after deposition in the absence and presence of ungulates. A total of 211 acorns were analyzed.

Fig. 3. Results from simulations of the probability transition model for acorn dispersal. (A) Proportion of acorns escaping predation in the presence or absence of environmental stressors (i.e. full moon, ungulates present or predator scent, black bar) in comparison to more optimal conditions (i.e. new moon, ungulates absent, no predator scent, grey bar). Size of predated (yellow) and viable (blue) acorns under (B) new *vs* full moon conditions and in the presence or absence of (C) ungulates and (D) predator scent. Bars represent mean values (±s.e.) across 1000 simulations.

The graph shows Predator scent as (C) and Ungulates as (D)

Fig. 1

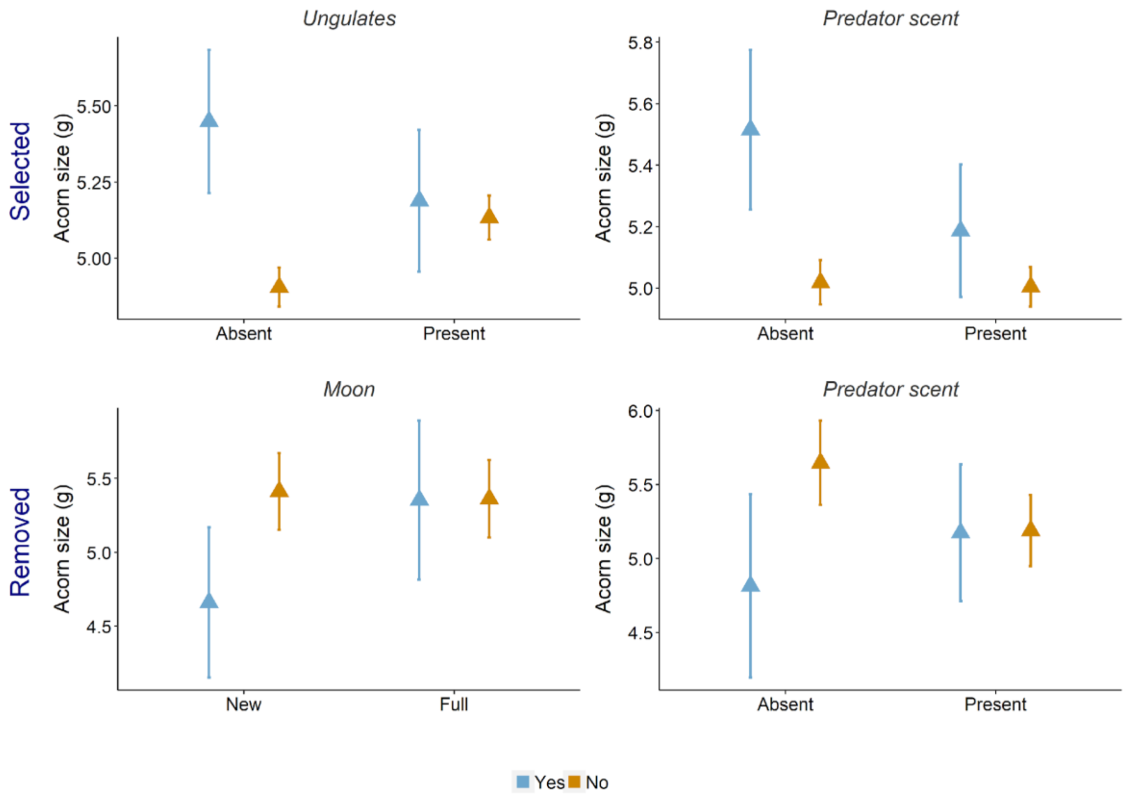
Ungulates
Predator scent
Moon
Predator scent
Selected
Removed
Acorn size (g)
5.50
5.25
5.00
5.8
5.6
5.4
5.2
5.0
6.0
5.5
4.5
Absent
Present
New
Full
Yes
No

Fig. 2

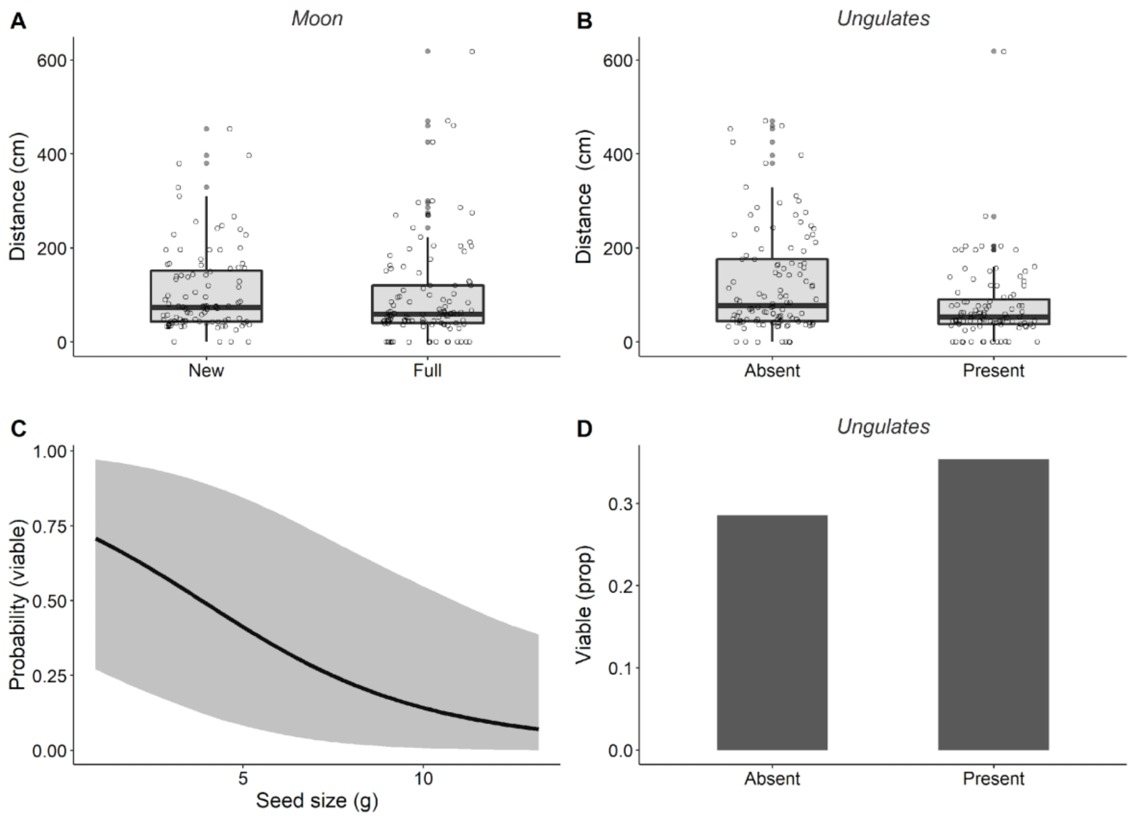
A
Moon
Distance (cm)
600
400
200
0
New
Full
B
Ungulates
Distance (cm)
600
400
200
0
Absent
Present
C
Probability (viable)
1.00
0.75
0.50
0.25
0.00
5
10
Seed size (g)
D
Ungulates
Viable (prop)
0.3
0.2
0.1
0.0
Absent
Present

Fig. 3

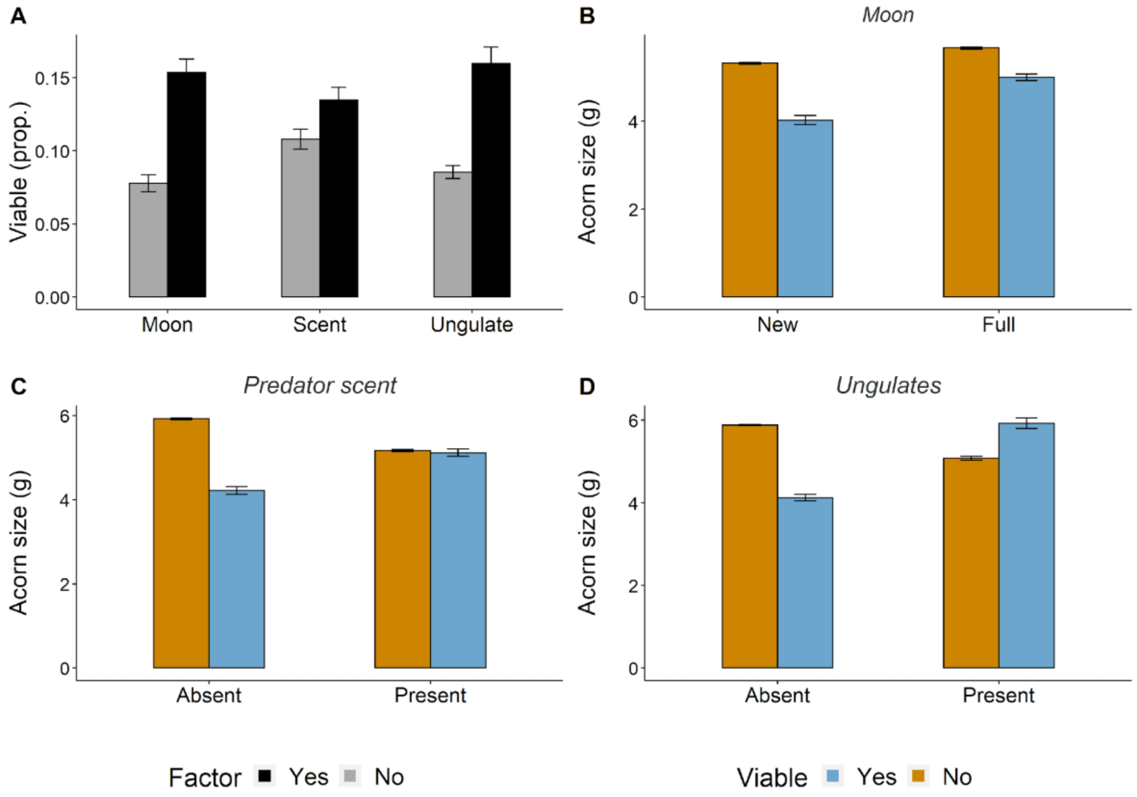

"Yes" is presented on the left in the legend, so it should be presented on the left in the graphs above.

Figure

Click here to access/download;Figure;Fig1.png

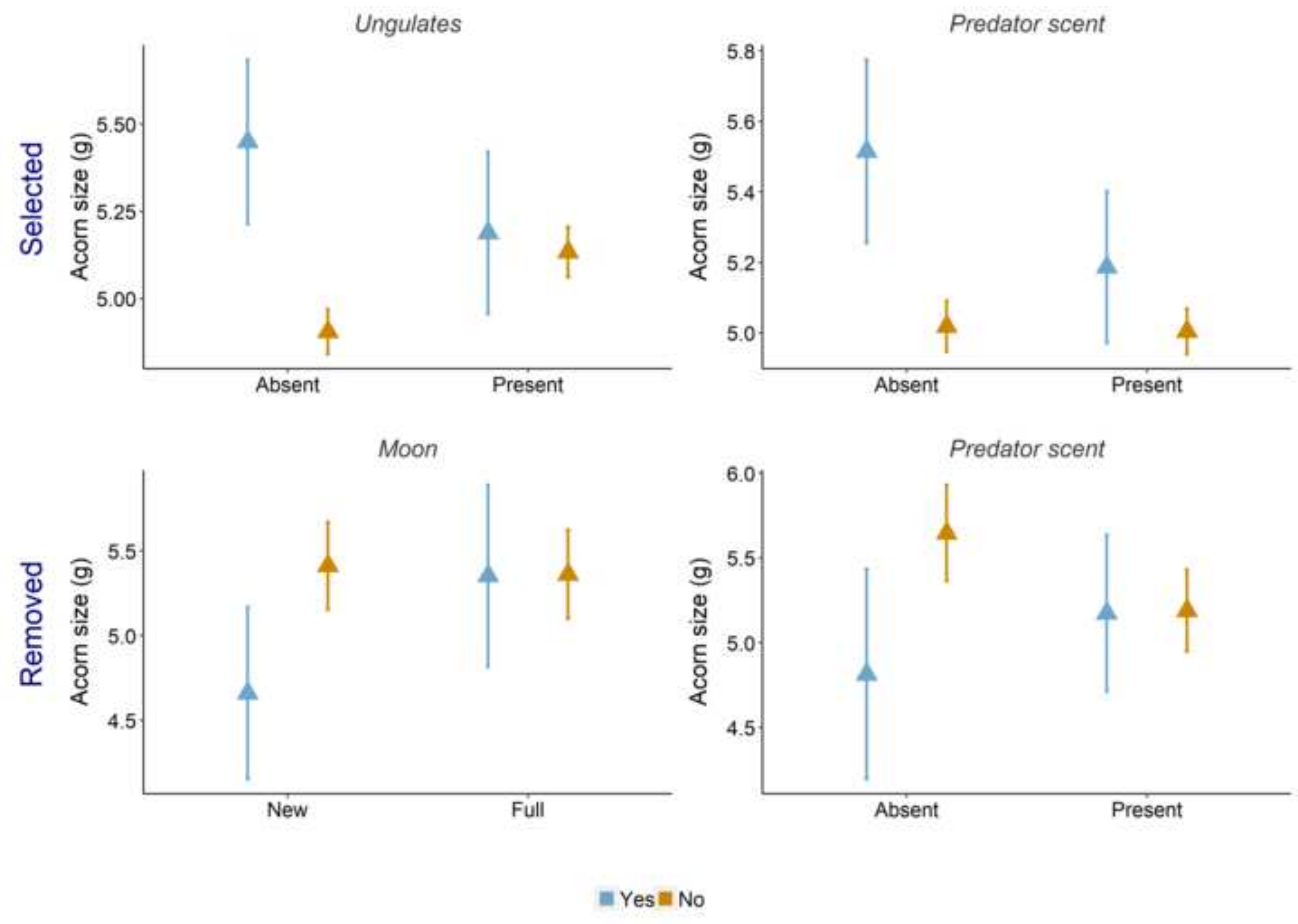

Figure

Click here to access/download;Figure;Fig2.png

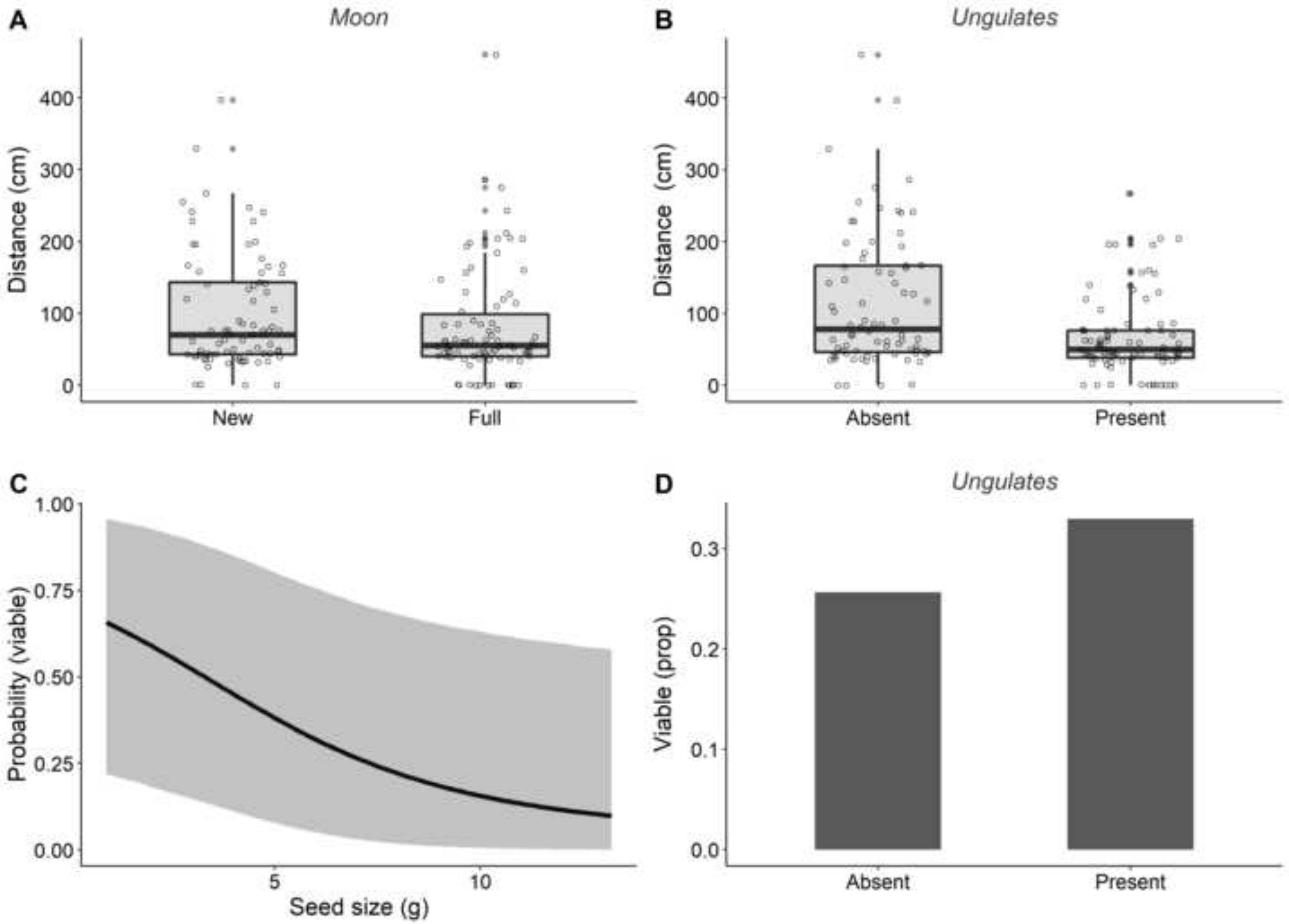

Figure

Click here to access/download;Figure;Fig3.png

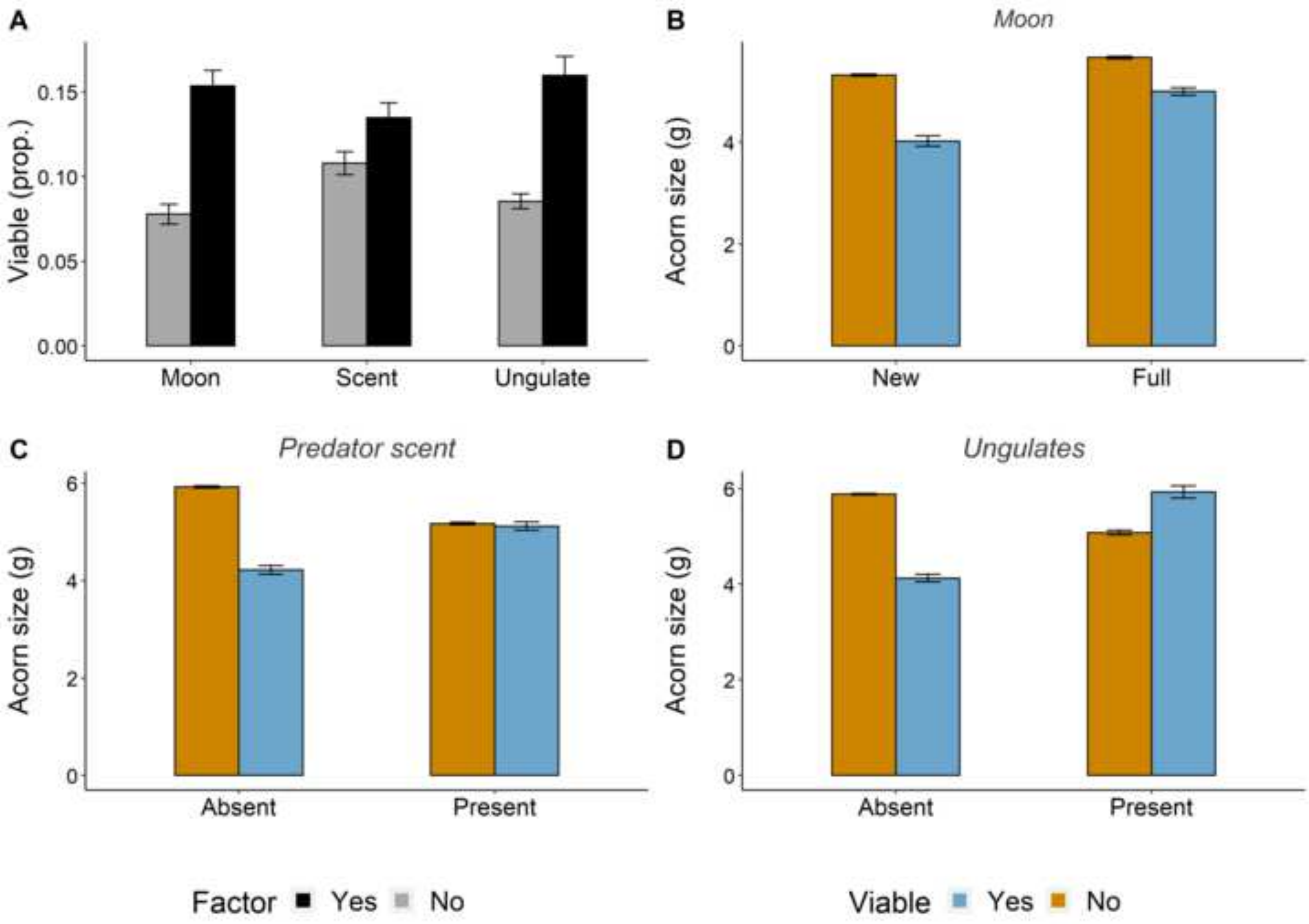

Click here to access/download
Supporting Information
MoranLopez_etal_BiolIntegrity_SupplInfo.doc

Click here to access/download
Supporting Information
MoranLopez_etal_BiolIntegrity_SupplMat5_Databases.xlsx

Biological integrity of dehesa ecosystems favors acorn dispersal over predation in the mouse-oak mutualism

Teresa Morán-López[1], Jesús Sánchez-Dávila[2], Ignasi Torre[3], Alvaro Navarro-Castilla[4], Isabel Barja[4, 5], Mario Díaz[2]*

1 Laboratorio Ecotono, INIBIOMA-CONICET, Universidad Nacional del Comahue, Black River, Argentina, **2** Department of Biogeography and Global Change (BGC-MNCN-CSIC), National Museum of Natural Sciences, CSIC, c/Serrano 155bis, E-28006 Madrid, Spain, **3** Museu de Ciències Naturals de Granollers (MCNG), E-08402 Granollers, Barcelona, Spain, **4** Departament of Biology, Unit of Zoology, Faculty of Sciences, Universidad Autónoma de Madrid, C/Darwin 2, Campus Universitario de Cantoblanco, 28049 Madrid, Spain, **5** Centro de Investigación en Biodiversidad y Cambio Global (CIBC-UAM) Universidad Autónoma de Madrid, C. Darwin 2, E-28049 Madrid, Spain

** Correspondence: E-mail Mario.Diaz@ccma.csic.es; tel +34 91 782 21 02; fax +34 91 564 08 00*

Running headline: Predation and competition favor dispersal in mouse-oak interaction

Word count: abstract: 272; main text: 4029; 69 references, 2 tables, 3 figures, 5 Supplementary files

Abstract

Scatter-hoarding decisions by rodents are key for the long-term maintenance of oak populations in which avian seed dispersers are absent or inefficient. Decisions are determined by seed value, competition and predation risk. Therefore, they can be influenced by the integrity of the biological system (i.e. trees, rodents, ungulate competitors, and rodent predators). In this work, we manipulate and model the mouse-oak interaction in a Spanish dehesa, an anthropogenic savanna system in which nearby areas can show contrasting levels of mice and ungulate densities, as well as antipredatory cover. First, we conducted a large-scale cafeteria field experiment, where we modified ungulate presence and predation risk, and followed mouse scatter-hoarding decisions under contrasting levels of moonlight and acorn availability. Then, we estimated the net effects of competition and risk by means of a transition probability model that simulated mouse scatter-hoarding decisions. Our experimental and simulation results show that mice are able to adapt their foraging decisions to the environmental context and that such changes affect the probability of acorn dispersal. Under stressful conditions (predation risks and presence of ungulates), mice foraged opportunistically mobilizing large and small seeds. In addition, lack of antipredatory cover around dehesa trees limited the transportation of acorns, but also precluded mice activities outside tree canopies. As a result, post-dispersal predation rates were reduced and large acorns had a higher probability to survive. Overall, our work points out that inter-specific interactions preventing efficient foraging by scatter-hoarders can promote acorn dispersal. Therefore, the maintenance of the full set of producers, seed dispersers and consumers, as well as predators of dispersers in ecosystems, may be key for promoting seed dispersal effectiveness in conditional mutualisms.

Keywords: synzoochory, scatter-hoarding, competition, risk, seed fate

Introduction

Scatter-hoarders are key seed dispersers in temperate and Mediterranean forests dominated by oaks [1,2]. Nut dispersal by scatter-hoarders (synzoochory) is a classical plant-animal conditional mutualism. The outcome of the interaction may be either mutualistic (dispersal) or antagonistic (predation) depending on the proportion of seeds consumed *vs.* cached and not retrieved [3]. The balance between mutualism and antagonism is contingent on intrinsic properties of interaction partners (e.g. propensity of animals to store food) as well as on the ecological setting in which the interaction occurs [2]. As a result, the net effects of synzoochory can be highly dynamic in space and time, making it difficult to predict its outcomes along environmental gradients and ecological timescales [4,5].

Acorn dispersal depends on scatter-hoarder corvids and rodents. Corvids mobilize acorns tens to hundreds of meters providing seed dispersal services to oaks [6], whereas rodents transport acorns locally and a high proportion of them are finally predated [7,8]. Nonetheless, several mouse species (*Apodemus*, *Mus*, *Peromyscus*) can become important acorn dispersers in landscapes where scatter-hoarder corvids are absent [9] or become inefficient [10]. Two main external factors modulate mouse foraging decisions: competition for seeds and predation risks [11–13]. Intraspecific competition and the presence of ungulates tend to encourage seed mobilization [3,14–16]. Especially, when predating seeds *in situ* is more time-consuming than carrying them away. In this context, scatter-hoarding facilitates stockpiling seeds before they are depleted by competitors [12,17]. Risk perception, in turn, depends on factors that affect exposure to predators (e.g. moonlight) as well as direct cues of their presence (e.g. scent) [18–23]. In general, intermediate risks can promote mobilization when mice carry away seeds to manipulate them in safer locations or when handling times of consuming seeds *in situ*

are too long [12]. However, if lack of cover in the vicinity of trees trigger predation risks, acorn mobilization distances and caching rates can be significantly reduced [13,24]. In general, suboptimal conditions for foraging mice (i.e. competition and predation risk) tend to favor scatter-hoarding over *in situ* predation. In the absence of stress, rodents usually act as efficient seed predators consuming, immediately or soon afterwards, seed crops under the canopy of mother trees [2].

Beyond the environmental conditions of plant-animal encounters, seed size can affect the initial outcomes of the interaction (selected, eaten or cached) as well as post-dispersal processes such as germination and seedling survival. Larger seeds are usually selected and preferentially cached because they provide higher food rewards [7,25–29]. In addition, seed size enhances post-dispersal seedling survival and establishment [30], which is a key component of dispersal effectiveness [31] in scatter-hoarder animals [32]. Nonetheless, the strength and even sign of acorn size effects on mouse foraging decisions are not unequivocal, but context-dependent. Larger acorns are most preferred when food is scarce [33–35], but may be avoided when longer handling times [26] diminish their profitability [36,37] or result in unaffordable predation risks during manipulation [11,12]. Therefore, to have a full picture of mouse role on acorn dispersal we need to account for seed size effects on scatter-hoarding decisions as well as the influence of competition and risk.

In this context, dehesas represent an excellent study system to assess the main factors modulating mouse foraging decisions, and hence, acorn dispersal. They are savanna-like habitats, simpler than natural forests but diverse enough to maintain all key elements influencing the mouse-oak conditional mutualism. In spite of this, dehesas, as well as other man-made systems dominated by scattered trees, suffer from a chronic lack of tree regeneration that compromises its long-term sustainability [38,39]. Depending on the

local intensity of management, nearby areas can have contrasting levels of shrub cover, mice densities and competition with ungulates [18,40]. In addition, the community of predators is simpler than in forest areas, facilitating the experimental manipulation of direct cues of risks [23]. In this work we take advantage of a large-scale experiment of ungulate exclosure in a Mediterranean dehesa to (1) quantify acorn size effects across different stages of the dispersal process (from seed choices to initial fates); and (2) evaluate if size effects are consistent across contrasting scenarios of predation risk and competition (inter- and intraspecific). In addition, we parameterized a transition probability model that assembled all scatter-hoarding decisions by mice to quantify and tease apart the net effect of competition and risk on early stages of the acorn dispersal process. We expected that suboptimal conditions for mice (i.e. competition and risk) would constrain their ability to forage efficiently, thus benefiting acorn dispersal over predation.

Methods

Study area and species

Field work was carried out in the holm oak *Quercus ilex* dehesa woodlands of the Cabañeros National Park (Central Spain, Ciudad Real province, 39°24' N, 38°35 W). Dehesas are savanna-like man-made habitats resulting from shrub removal and tree thinning and pruning to enhance herb growth for livestock [41]. The studied dehesas were opened from the original Mediterranean forests in the late 1950s. Currently they have no livestock but wild ungulate populations of red deer *Cervus elaphus* and wild boars *Sus scrofa*. Deer densities were around 0.14 ind./ha [42] and boars are abundant but at unknown densities [43]. Acorns fall from trees from mid-October to late November [9].

The study area covers around 780 ha, with two ungulate exclosures (site 1 and 2) made with wire fences 2 m tall and 32 cm x 16 cm mesh of 150 ha and 4.65 ha separated from each other by 1500 m. The exclosures prevent the entrance of ungulates but not of mesocarnivores (mainly common genets *Genetta genetta* and red foxes *Vulpes vulpes*; pers. obs. based on scat searches) and raptors. In the study area, average tree density was 20.4 trees ha-1, although site 1 had higher mean density than site 2 (30.0±2.6 and 30.1±3.4 inside and outside ungulate exclosure in site 1, and 7.4±0.4 and 7.4±0.3 in site 2; mean±SE). Shrub cover was <1%, as measured on aerial photographs and vegetation surveys both under canopies and outside them [23]. Mean tree canopy radius was 4.2±0.7 m (range 2.5-7.1 m). The Algerian mouse (*Mus spretus*) is the most abundant scatter-hoarding rodent in the area [44], and it is a common prey of genets and other generalist predators [45,46].

Experimental design

Tree occupancy by mice was established by means of live trapping using Sherman traps (23 × 7.5 × 9 cm; Sherman Co., Tallahassee, USA) baited with canned tuna in olive oil mixed with flour and a piece of apple. Water-repellent cotton was provided to prevent the cooling of the individual captured overnight. Traps were set during two consecutive days during the new moon of January 2012. High capture probability of *M. spretus* (detectability: 0.88±0.03SE; [47] allowed to consider false negatives in occupancy unlikely. Among trees known to be occupied by Algerian mice, we randomly selected ten trees inside and ten outside in each of the two exclosures (40 focal trees in total).

We paired focal trees according to their proximity and we randomly assigned a predator scent treatment to one of them. Predator scent treatment consisted of placing fresh genet feces (10 g) mixed with distilled water close to a corner of the cages where acorns were

placed [23]. Genets are generalist predators whose presence and scats are known to influence rodent behavior [20,22,48]. Fresh feces were collected from two captive common genets housed in the Cañada Real Open Center (Madrid, Spain).

Fresh acorns were collected from holm oaks growing near the study area in October 2011 and stored dry in a cooler (4 ºC) until use. Sound acorns, with no marks of insect damage [45], were weighed with a digital balance to the nearest 0.01 g. Groups of 15 acorns in three categories were randomly selected (5 each, large, >10 g; medium-sized, 5-10 g, and small, 1-5 g). Acorns were placed under the canopy of each focal tree inside a 50 cm × 50 cm x 15 cm galvanized-steel cage to prevent acorn consumption by birds or ungulates [44]. Cages were located 1.2 m on average (range 0.3-2.7 m) from focal tree trunks. A metal wire (ø 0.6 mm, 0.5 m length) with a numbered plastic tag was attached to each acorn [49]. After removing any naturally-present acorn within the cages, we randomly placed acorns in the intersection of a 3 rows x 5 columns grid. To track mouse choices, acorn size for each position was noted. Acorns were left exposed to mice for three consecutive nights, then removed. Mobilized acorns were located by looking at the plastic tags during the following days (24 and 72 hours). We tracked the status of acorns that were mobilized and not predated throughout the experiment. However, outside exclosures we were often unable to tease apart the disappearance of mobilized acorns due to predation by rodents from that of ungulates. Therefore, this long-term monitoring was not included in the analyses. To account for changes in night brightness and acorn availability [21,50], the cafeteria experiment was repeated four times during the full-moon and new-moon periods of November 2011 and February 2012. No official permits or protocol approvals were legally necessary since we did not manipulate individual mice except for checking whether trees were occupied or not by means of live traps. We followed Guidelines of the American Society of Mammalogists

for the use of wild mammals in research [51]. We performed all manipulations with disposable latex gloves, to avoid effects of human odor on rodent behavior [52].

Mouse foraging behavior

A video-camera OmniVision CMOS 380 LTV (OmniVision, Santa Clara, USA) (3.6 mm lens) monitored mouse foraging activity within each cage. Cameras were set on 1.5 m tall tripods located 2.5 m from each cage, powered by car batteries (70 Ah, lead acid) connected to a solar panel (ono-silicon erial P_20; 20 w). Video-cameras were connected to ELRO recorders with dvr32cards (ELRO, Amsterdam, Netherlands) and took continuous recording for three consecutive days autonomously (recorded in quality at 5 frames s^{-1}). Events with rodent activity, from the entry of the individual into the cage up to the exit from it, were located and separated using Boilsoft Video Splitter software (https://www.boilsoft.com/videosplitter/) [48]. Within each event we noted which acorn was manipulated and whether it was removed outside the cage. For removed acorns we measured mobilization distance (cm) and noted its status (predated or not after transportation).

Data analysis

To assess acorn choice by rodents, we fitted a hierarchical multinomial model. Our response variable was acorn selection (yes/no). Our explanatory variables were: acorn size (g), moon phase (new/full), month (February, November), ungulate presence (yes/no), predator scent (yes/no), acorn availability in the cage (g) and the two-way interactions between size and environmental effects. Local acorn availability was measured as total acorn mass in the cage during the event. Both, acorn size and availability were scaled previous to the analyses (mean = 0, sd = 1) so that we could

compare the magnitude of covariate effects. Focal tree was introduced as a random factor in the intercept term to account for repeated sampling during the experiment. To evaluate the effects of acorn size on the probability of removal, we used a hierarchical logistic model. Our response variable was whether a selected acorn was mobilized outside the cage or not (yes/no). Our explanatory variables and random effects were the same as in the multinomial model.

Subsequently, we analyzed the effect of acorn size and environmental covariates (and their two-way interaction) on seed dispersal. Our response variables were mobilization distances (cm, log-transformed) and deposition status (viable or predated). We used a hierarchical Gaussian model in the former case, and a hierarchical logistic model in the latter. Our explanatory variables and random effects were the same as in the previous models. In all four models (selection, removal, mobilization distance and initial fate) we used uninformative priors (Supplementary File 1). All analyses were performed employing a Bayesian approach with JAGS 3.4.0 [53]. We checked for convergence for all model parameters (Rhat < 1.1) and that the effective sample size of posterior distributions was high (>800). We estimated the mean and credible interval of posterior distributions. Also, we calculated the proportion of the posterior distribution with the same sign of the mean (f) and evaluated the predictive power of our models by means of posterior predictive checks (Supplementary Files 1 and 2).

Simulating scatter-hoarding decisions

To estimate the joint effect of seed size, competition and risk on acorn dispersal we designed a probability transition model in which simulated mice adapted their foraging behavior to the environmental context (Supplementary File 3). Before model run, we parameterized mouse scatter-hoarding decisions (from selection to initial fate) following

the same scheme of regressions explained in the previous section. Nonetheless, here we only used data from November, the period of peak acorn falling in our study system. Consequently, we did not include month as a covariate. For each behavioral submodel (selection, removal and initial fate), we obtained posterior distributions of parameters by running 50000 iterations in three chains (in all cases Rhat< 1.1, and Neff> 1000).

Model setup mimics our experimental design, 20 trees outside and 20 inside exclosures paired according to a predator scent treatment (presence *vs* absence). Simulations begin under new moon conditions with focal trees offering 15 acorns of large, medium and small sizes (5 each). Acorn size is sampled from empirical distributions of these size categories. In each focal tree, the number of foraging events is drawn from a Poison distribution with mean equal to the average number of events observed in the corresponding moon phase. During each foraging event, simulated mice decide which acorn to handle and whether to remove it or not. If removed, mice decide whether to predate it or not after mobilization, and acorn availability in the cage is updated. Once all foraging events (of all trees) are simulated, acorn dispersal is modelled under full moon conditions (Supplementary File 3, Fig. S1).

For each model run we sampled parameter of behavioral submodels (selection, removal and deposition) from posterior distributions fitted to data. Thus, in our simulations, mice adapted their decisions to acorn size and availability (in the experimental cage), characteristics of the focal tree (i.e. ungulate and predator scent presence), and the moon phase in which the foraging event occurs (new or full moon). After each model run (dispersal under new and full moon conditions), the program tracked the size and status of handled acorns and the environmental covariates in which the foraging event occurred. We run the model 1000 times and plotted deposition rates of viable acorns and their size with respect to the moon phase and tree characteristics (predator scent and

ungulate presence). See Supplementary File 3 for detailed model specifications and Fig. S1 for a summary of the process overview.

Results

Before setting the cafeteria experiments in November, we removed from cages 53.3 acorns/m^2 on average (range: 0-104). No acorn was found in February. We monitored 2280 acorns under 38 focal trees. We detected mouse activity in 18 and 26 trees in the new and full moon of November, and in 26 and 24 trees in the new and full moon of February, respectively. Mice manipulated 1677 acorns. Out of them, 267 were mobilized outside cages and 211 (79%) were relocated.

Foraging decisions in the focal tree: selection and removal

In general, mice selected larger acorns, but the positive effect of size was modulated by environmental conditions. Size-driven selection preferentially occurred in the absence of competition with ungulates (Fig. 1A) and predator scent (Fig. 1B). In addition, mouse selectivity was enhanced under low local acorn availability (Table 1, selection). Among selected acorns, mice preferentially removed smaller ones. Such selective behavior occurred when risks were low due to reduced night brightness (new moon, Fig. 1C) or lack of predator scent (Fig. 1D), as well as when ungulates were absent (Table 2). Acorn availability at local and landscape scales did not modify size effects, although they changed mobilization rates. Rates were lower during the acorn fall peak (13% in November *vs* 24 % in February), whereas acorn availability in cages enhanced removal (Table 2, removal).

Foraging decisions during dispersal: mobilization distances and predation

Mice mobilized acorns closer under new moon conditions (Fig. 2A) and when ungulates were present (Fig. 2B). During lean periods (February) mobilization distances and post-dispersal predation increased (Table 2, Month). In addition, larger acorns were preferentially consumed (Fig. 2C), though the presence of ungulates and full moon conditions attenuated this negative effect (Fig. 2D, Table 2).

Transition probability model for acorn dispersal

Under optimal conditions (new moon, no predator scent or ungulates), post-dispersal predation rates were higher (Fig. 3A) and simulated mice preferentially consumed large acorns (i.e. viable acorns -blue bars- were smaller, Fig. 3B-D). However, predation risks and ungulate presence precluded acorn consumption after mobilization and attenuated selection. As a result, the proportion and size of viable acorns increased (Fig. 3A and B-D).

Discussion

Overall, our work shows that mice are able to adapt their foraging decisions to perceived predation risks and competition for seeds. Also, that such behavioral adjustments affect the fate of acorns at initial stages of the dispersal process. When relaxed, mice preferentially consumed large acorns and removed small ones. Furthermore, mobilized seeds were more likely to be predated. In contrast, under stressful conditions (increased predation risk and competition) mice foraged opportunistically and reduced their activity outside tree canopies. As a result, predation rates after mobilization decreased, and larger acorns had a higher probability to survive, at least in the short term. This bolsters the idea that interactions with third-party players

can modify the qualitative component of dispersal effectiveness of scatter-hoarding rodents [12,15,54].

As expected, larger and more valuable acorns were preferentially handled by mice, which adapted this behavior to the environmental context [12]. In line with previous work, mice foraged opportunistically in trees with predator scent, probably because they devoted more time to vigilant behaviors [15,48] at expenses of acorn discrimination [21]. In contrast, acorn availability effects did not follow the expectations of increased selectivity in scenarios of food depletion [28,54,55]. Seed size effects were similar between acorn fall peaks and lean periods. Furthermore, mice foraged randomly when ungulates were present, while they selected larger seeds within exclosures. We expected that competition for acorns would promote a more selective behavior to ensure the acquisition of more valuable food items [28]. However, we found the opposite probably due to some particularities of our system. Dehesas are characterized by high acorn production and scarce shrub cover (<1%) around trees [41,56,57]. Under such circumstances, the effects of increased predation risks outside tree canopies can outweigh those of competition leading to a rapid and random harvesting of seeds [21]. In contrast, within ungulate exclosures, reduced grazing and soil compaction has promoted taller resprouts under canopies and increased cover of herbs and tussocks around trees [58]. As a result, in the absence of ungulates mice can forage under shelter, devoting less time to vigilant behaviors [48]. The fact that in the presence of ungulates mice foraged opportunistically, point out that in dehesas predation risks modulate acorn selection. Also, changes in vegetation cover due to ungulate presence can strongly affect mouse scatter-hoarding decisions [17].

Larger acorns tend to be carried away, mobilized farther and preferentially cached in forest habitats [7,27,59,60]. However, in our study larger acorns had a higher

probability of being predated (*in situ* and after transportation) and seed size did not affect mobilization distances. Again, these results highlight that environmental conditions in dehesas are particularly harsh for rodents. In general, rodents preferentially mobilize small seeds when the costs of carrying large ones result unaffordable [12]. In the presence of ungulates, low antipredatory cover and high trampling risks may have triggered transportation costs [13,61], deterring mice from carrying large seeds away. Seed size effects were not fixed, but depended on direct and indirect cues of risk. Preferential removal of small seeds only occurred in trees with no predator scent or under new moon conditions, reflecting that only when risks are mild mice can take the time to select among the seeds available [15,21].

Regarding post-dispersal survival, we expected higher predation when acorns were deposited close to tree canopies [13,24], but this relationship blurred in our system. Outside ungulate exclosures, larger acorns had a higher probability of escaping predation in spite of being mobilized nearby source trees. In dehesas, the pervasiveness of open land cover forces mice to concentrate their activities beneath canopies [13,23,58], and decreases the likelihood that mobilized acorns are encountered and consumed [62]. Accordingly, in our simulations, suboptimal conditions (due to increased risks or ungulate presence) discouraged mice from consuming seeds after mobilization. Consequently, predation rates were reduced and larger acorns had a higher probability to survive. In Mediterranean systems, seedlings from larger acorns are more resistant to summer drought [30,63], which represents the main recruitment bottleneck for oak regeneration [39,64]. Therefore, higher survival rates of large acorns suggest that intermediate levels of stress can enhance the qualitative component of seed dispersal effectiveness by mice (as suggested by [65]).

This work builds on previous research analyzing the effects of competition and risk on mouse foraging behavior in dehesas [48]. Here, by accounting for all stages of scatter-hoarding decisions (from initial manipulation to consumption after mobilization [60]), and including the entire acorn fall season [27] as well as contrasting moon light conditions [21], we obtained a more in-depth understanding of the main drivers of dispersal effectiveness. Moreover, our transition probability model allowed us to assemble all stages of the scatter-hoarding process, and hence, to estimate the net effects of competition and predation risks on initial seed fate. Nonetheless, long-term survival of mobilized acorns was not assessed, and hence, our results do not allow us to estimate actual seed dispersal effectiveness [31]. Throughout the autumn, retrieval rates of cached acorns can be high (less than 5% of offered acorns survive to the next spring [7,66]). In addition, lack of shrub cover in dehesas can limit recruitment due to seedling dry out during summer [9,67]. Therefore, it remains an open question whether cache recovery or seedling death can outweigh the positive effects of ungulate presence and risk on initial stages of acorn dispersal.

Quantifying dispersal effectiveness in animals that mobilize a large number of seeds, but predate a high proportion of them is a challenging task. High mobilization rates can result in an ecologically relevant number of seedlings in spite of intensive predation [2,7]. However, to obtain reliable estimates of post-dispersal survival, we need to track the fate of a large (and often unaffordable) number of seeds. For instance, between 1.3 and 3% of offered acorns survive to the next spring [7,66]. Thus, to estimate the effects of environmental factors and seed size on long-term cache survival (sample size > 200) between 6700 and 15000 acorns should have been tracked. A way to circumvent this problem, is directly sowing acorns and monitoring artificial caches. Such approach does not allow to evaluate foraging decisions made by cache owners, but informs about post-

dispersal survival from pilferers and ungulates, as well as the probability of emergence and early establishment [67,68]. Once this information is available, it could be easily included in our transition probability model (following [69]), allowing to evaluate if changes in mouse foraging decisions have an imprint on seedling recruitment.

Concluding remarks

Our mechanistic approach provides new insights about the joint effect of habitat structure, competition and risk on the foraging behavior of mice and its potential consequences on acorn dispersal. In the presence of ungulates and when predation risks were high, mice acted as opportunistic foragers and concentrated their activities beneath tree canopies. As a result, predation rates decreased and larger acorns had a higher probability to survive (at least in the short term). These results suggest that inefficient foraging by rodents can promote acorn dispersal. Also, they highlight the importance of competition and risk on the spatial and temporal dynamism of synzoochorous interactions [2]. Finally, though future work is needed to estimate long-term cache survival and seedling establishment, our results support the view that biological integrity (presence of the full set of producers, consumers, dispersers and predators) can facilitate seed dispersal effectiveness in synzoochorous mutualisms [2,54]. This may be particularly important in man-made habitats like dehesas, which depend on conditional mutualisms to ensure their long-term sustainability [39,57]

Supporting information

S1. Structure of models and priors

S2. Posterior predictive checks

S3. Specifications of transition probability model for acorn dispersal

S4. Code for the transition probability model

S5. Databases

Acknowledgements

D. López, M. Fernández and C. L. Alonso helped during fieldwork. D. López, B. Ramos and M. de Pablo pre-processed the video recordings, and D. Gallego, D. Valero, A. Velasco, C. J. González and E. Sánchez visualized the recordings noting seed choices. Authorities of the Cabañeros National Park provided the official permissions to carry out field experiments. J. España provided common genet scats.

Author contributions

MD conceived and executed the field experiment with the aid of IT, IB, TM-L and AN-C. JS-D compiled the data and performed preliminary analyses and drafts. TM-L executed the final data analyses and proposed the final main focus of the paper. TM-L and MD wrote the final version of the paper on former versions drafted by JS-D and contributed by all authors.

References

1. Vander Wall SB. The evolutionary ecology of nut dispersal. Bot Rev. 2001;67: 74–117.

2. Gómez JM, Schupp EW, Jordano P. Synzoochory: the ecological and evolutionary relevance of a dual interaction. Biol Rev. 2019;94: 874–902.

3. Theimer T. Rodent scatterhoaders as conditional mutualists. In: Forget PM, Lambert JE HP, editor. Seed Fate: Predation, dispersal, and seedling establishment. Wallingford: CAB International; 2005. pp. 283–295.

4. Bronstein JL. Conditional outcomes in mutualistic interactions. Trends Ecol Evol. 1994;9: 214–217.

5. Sawaya GM, Goldberg AS, Steele MA, Dalgleish HJ. Environmental variation shifts the relationship between trees and scatterhoarders along the continuum from mutualism to antagonism. Integr Zool. 2018;13: 319–330.

6. Pesendorfer MB, Sillett TS, Koenig WD, Morrison SA. Scatter-hoarding corvids as seed dispersers for oaks and pines: a review of a widely distributed mutualism and its utility to habitat restoration. Condor Ornithol Appl. 2016;118: 215–237.

7. Gómez JM, Puerta-Piñero C, Schupp EW. Effectiveness of rodents as local seed dispersers of Holm oaks. Oecologia. 2008;155: 529–537.

8. Kellner KF, Lichti NI, Swihart RK. Midstory removal reduces effectiveness of oak (Quercus) acorn dispersal by small mammals in the Central Hardwood Forest region. For Ecol Manage. 2016;375: 182–190.

9. Pulido F, García E, Obrador JJ, Moreno G. Multiple pathways for tree regeneration in anthropogenic savannas: incorporating biotic and abiotic drivers into management schemes. J Appl Ecol. 2010;47: 1272–1281.

10. Morán-López T, Alonso CL, Díaz M. Landscape effects on jay foraging behavior decrease acorn dispersal services in dehesas. Acta Oecologica. 2015;69. doi:10.1016/j.actao.2015.07.006

11. Steele MA, Contreras TA, Hadj-Chikh LZ, Agosta SJ, Smallwood PD, Tomlinson CN. Do scatter hoarders trade off increased predation risks for lower rates of cache pilferage? Behav Ecol. 2014;25: 206–215.

12. Lichti NI, Steele MA, Swihart RK. Seed fate and decision- making processes in scatter- hoarding rodents. Biol Rev. 2017;92: 474–504.

13. Morán-López T, Wiegand T, Morales JM, Valladares F, Díaz M. Predicting forest management effects on oak–rodent mutualisms. Oikos. 2016;125. doi:10.1111/oik.02884

14. Puerta- Piñero C, María Gómez J, Schupp EW. Spatial patterns of acorn dispersal by rodents: do acorn crop size and ungulate presence matter? Oikos. 2010;119: 179–187.

15. Sunyer P, Munoz A, Bonal R, Espelta JM. The ecology of seed dispersal by small rodents: a role for predator and conspecific scents. Funct Ecol. 2013;27: 1313–1321.

16. Morán- López T, Fernández M, Alonso CL, Flores- Rentería D, Valladares F, Díaz M. Effects of forest fragmentation on the oak–rodent mutualism. Oikos. 2015;124: 1482–1491.

17. Zhang H, Wang Y, Zhang Z. Domestic goat grazing disturbance enhances tree seed removal and caching by small rodents in a warm-temperate deciduous forest in China. Wildl Res. 2009;36: 610–616.

18. Díaz M, Gonzalez E, Munoz-Pulido R, Naveso MA. Effects of food abundance and habitat structure on seed-eating rodents in Spain wintering in man-made habitats. Zeitschrift für Säugetierkd. 1993;58: 302–311.

19. Orrock JL, Danielson BJ, Brinkerhoff RJ. Rodent foraging is affected by indirect, but not by direct, cues of predation risk. Behav Ecol. 2004;15: 433–437.

20. Díaz M, Torre I, Peris A, Tena L. Foraging behavior of wood mice as related to presence and activity of genets. J Mammal. 2005;86: 1178–1185.

21. Perea R, González R, San Miguel A, Gil L. Moonlight and shelter cause

differential seed selection and removal by rodents. Anim Behav. 2011;82: 717–723.

22. Navarro- Castilla Á, Barja I. Antipredatory response and food intake in wood mice (Apodemus sylvaticus) under simulated predation risk by resident and novel carnivorous predators. Ethology. 2014;120: 90–98.

23. Navarro-Castilla Á, Barja I, Díaz M. Foraging, feeding, and physiological stress responses of wild wood mice to increased illumination and common genet cues. Curr Zool. 2018;64: 409–417.

24. Morán-López T, Fernández M, Alonso CL, Flores-Rentería D, Valladares F, Díaz M. Effects of forest fragmentation on the oak-rodent mutualism. Oikos. 2015;124. doi:10.1111/oik.02061

25. Pons J, Pausas JG. Rodent acorn selection in a Mediterranean oak landscape. Ecol Res. 2007;22: 535–541.

26. Muñoz A, Bonal R. Are you strong enough to carry that seed? Seed size/body size ratios influence seed choices by rodents. Anim Behav. 2008;76: 709–715.

27. Sunyer P, Espelta JM, Bonal R, Muñoz A. Seeding phenology influences wood mouse seed choices: the overlooked role of timing in the foraging decisions by seed-dispersing rodents. Behav Ecol Sociobiol. 2014;68: 1205–1213.

28. T. M-L, Valladares F, Tiribelli F, Pérez-Sepúlveda JE, Traveset A, Diaz M. Fragmentation modifies seed trait effects on scatter-hoardersâ foraging decisions. Plant Ecol. 2018.

29. Pérez- Ramos IM, Urbieta IR, Maranón T, Zavala MA, Kobe RK. Seed removal in two coexisting oak species: ecological consequences of seed size, plant cover and seed- drop timing. Oikos. 2008;117: 1386–1396.

30. Gómez JM. Bigger is not always better: conflicting selective pressures on seed size in Quercus ilex. Evolution (N Y). 2004;58: 71–80.

31. Schupp EW, Jordano P, Gomez JM. Seed dispersal effectiveness revisited: a conceptual review. New Phytol. 2010;188: 333–353. doi:10.1111/j.1469-8137.2010.03402.x

32. Zwolak R, Crone EE. Quantifying the outcome of plant–granivore interactions. Oikos. 2012;121: 20–27.

33. Li H, Zhang Z. Effects of mast seeding and rodent abundance on seed predation and dispersal by rodents in Prunus armeniaca (Rosaceae). For Ecol Manage. 2007;242: 511–517.

34. Wang H, Cao G, Wang L, Yang Y, Zhang Z, Duan Y. Evaluation of pollinator effectiveness based on pollen deposition and seed production in a gynodieocious alpine plant, Cyananthus delavayi. Ecol Evol. 2017;7: 8156–8160.

35. Vander Wall SB. Masting in animal- dispersed pines facilitates seed dispersal. Ecology. 2002;83: 3508–3516.

36. Pulliam HR. Foraging efficiency, resource partitioning, and the coexistence of sparrow species. Ecology. 1985;66: 1829–1836.

37. Díaz M. Variability in seed size selection by granivorous passerines: effects of bird size, bird size variability, and ecological plasticity. Oecologia. 1994;99: 1–6.

38. Campos P, Huntsinger L, Oviedo JL, Starrs PF, Díaz M, Standiford RB, et al. Mediterranean oak woodland working landscapes. Landsc Ser. 2013;16.

39. Diaz M, Sánchez-Mejía T, Morán-López T. Long-term tree regeneration of fragmented agroforestry systems under varying climatic conditions. Habitat Modif Landsc Fragm Agric Ecosyst Implic Biodivers Landsc Multi-Functionality. 2022.

40. Munoz A, Bonal R, Díaz M. Ungulates, rodents, shrubs: interactions in a diverse Mediterranean ecosystem. Basic Appl Ecol. 2009;10: 151–160.

41. Pulido FJ, Díaz M, de Trucios SJH. Size structure and regeneration of Spanish

holm oak Quercus ilex forests and dehesas: effects of agroforestry use on their long-term sustainability. For Ecol Manage. 2001;146: 1–13.

42. Jiménez J. El ciervo (Cervus elaphus) en la zona oriental del Parque Nacional de Cabaneros. Madrid Org Autónomo Parques Nac. 2004.

43. Perea R, Gil L. Tree regeneration under high levels of wild ungulates: The use of chemically vs. physically-defended shrubs. For Ecol Manage. 2014;312: 47–54.

44. Díaz M, Alonso CL, Beamonte E, Fernández M, Smit C. Development of a long-term monitoring protocol of keystone organisms for the functioning of Mediterranean forests [in Spanish]. In: Ramírez L AB, editor. Proyectos de investigación en parques nacionales 2007-2010. Madrid: , Organismo Autónomo Parques Nacionales; 2011. pp. 47–75.

45. Palomo LJ, Justo ER, Vargas JM. Mus spretus (Rodentia: muridae). Mamm species. 2009; 1–10.

46. Torre I, Arrizabalaga A, Freixas L, Ribas A, Flaquer C, Díaz M. Using scats of a generalist carnivore as a tool to monitor small mammal communities in Mediterranean habitats. Basic Appl Ecol. 2013;14: 155–164.

47. Torre I, Raspall A, Arrizabalaga A, Díaz M. SEMICE: an unbiased and powerful monitoring protocol for small mammals in the Mediterranean Region. Mamm Biol. 2018;88: 161–167.

48. Gallego D, Morán-López T, Torre I, Navarro-Castilla Á, Barja I, Díaz M. Context dependence of acorn handling by the Algerian mouse (Mus spretus). Acta Oecologica. 2017;84. doi:10.1016/j.actao.2017.07.002

49. Xiao Z, Jansen PA, Zhang Z. Using seed-tagging methods for assessing post-dispersal seed fate in rodent-dispersed trees. For Ecol Manage. 2006;223: 18–23.

50. Diaz M. Rodent seed predation in cereal crop areas of central Spain: effects of

physiognomy, food availability, and predation risk. Ecography (Cop). 1992;15: 77–85.

51. Sikes RS, Gannon WL. Guidelines of the American Society of Mammalogists for the use of wild mammals in research. J Mammal. 2011;92: 235–253.

52. Wenny DG. Effects of human handling of seeds on seed removal by rodents. Am Midl Nat. 2002; 404–408.

53. Plummer M. JAGS: A program for analysis of Bayesian graphical models using Gibbs sampling. Proceedings of the 3rd international workshop on distributed statistical computing. Vienna, Austria.; 2003. pp. 1–10.

54. Schupp EW, Zwolak R, Jones LR, Snell RS, Beckman NG, Aslan C, et al. Intrinsic and extrinsic drivers of intraspecific variation in seed dispersal are diverse and pervasive. AoB Plants. 2019;11. doi:10.1093/aobpla/plz067

55. Stephens DW, Krebs JR. Foraging theory. Princeton university press; 2019.

56. Koenig WD, Díaz M, Pulido F, Alejano R, Beamonte E, Knops JMH. Acorn production patterns. Mediterranean oak woodland working landscapes. Springer; 2013. pp. 181–209.

57. Díaz M. Tree scattering and long-term persistence of dehesas: patterns and processes. Ecosistemas. 2014;23: 5–12.

58. Navarro-Castilla Á, Díaz Esteban M, Barja I. Does ungulate disturbance mediate behavioural and physiological stress responses in Algerian mice (Mus spretus)? A wild exclosure experiment. 2017.

59. Wang B, Chen J. Seed size, more than nutrient or tannin content, affects seed caching behavior of a common genus of Old World rodents. Ecology. 2009;90: 3023–3032.

60. Wang B, Ye C, Cannon CH, Chen J. Dissecting the decision making process of scatter- hoarding rodents. Oikos. 2013;122: 1027–1034.

61. Muñoz A, Bonal R. Rodents change acorn dispersal behaviour in response to ungulate presence. Oikos. 2007;116: 1631–1638.

62. Muñoz A, Bonal R. Linking seed dispersal to cache protection strategies. J Ecol. 2011;99: 1016–1025.

63. Bonito A, Varone L, Gratani L. Relationship between acorn size and seedling morphological and physiological traits of Quercus ilex L. from different climates. Photosynthetica. 2011;49: 75–86.

64. Pulido FJ, Díaz M. Regeneration of a Mediterranean oak: a whole-cycle approach. Ecoscience. 2005;12: 92–102.

65. Feldman M, Ferrandiz-Rovira M, Espelta JM, Muñoz A. Evidence of high individual variability in seed management by scatter-hoarding rodents: does 'personality'matter? Anim Behav. 2019;150: 167–174.

66. Perea R, San Miguel A, Gil L. Acorn dispersal by rodents: the importance of re-dispersal and distance to shelter. Basic Appl Ecol. 2011;12: 432–439.

67. Smit C, Díaz M, Jansen P. Establishment limitation of holm oak (Quercus ilex subsp. ballota (Desf.) Samp.) in a Mediterranean savanna–forest ecosystem. Ann For Sci. 2009;66: 1–7.

68. Gómez JM, Garcıa D, Zamora R. Impact of vertebrate acorn-and seedling-predators on a Mediterranean Quercus pyrenaica forest. For Ecol Manage. 2003;180: 125–134.

69. González-Castro A, Calviño-Cancela M, Nogales M. Comparing seed dispersal effectiveness by frugivores at the community level. Ecology. 2015;96: 808–818. doi:https://doi.org/10.1890/14-0655.1

Table 1. Summary table of the effects of size, moonlight, month, ungulate presence, predator scent and local acorn availability (and their interactions with size) on the probability of acorn selection and removal. A total of 1677 foraging events were analyzed.

Process	Fixed effect	Mean	HPD1	f	
	**Size**	**0.19**	**[0.09, 0.29]**	**1.00**	**
	Moon (Full)	0.03	[-6.22, 6.35]	0.50	
	Month (February)	-0.03	[-6.12, 6.06]	0.50	
	Ungulate (Yes)	-0.05	[-6.27, 6.16]	0.51	
	Scent (Yes)	0.01	[-6.16, 6.28]	0.50	
**Acorn selection**	Availability	-0.02	[-6.32, 6.19]	0.50	
	Size*Moon	0.07	[-0.03, 0.17]	0.93	
	Size*Month	-0.06	[-0.16, 0.04]	0.88	
	**Size*Ungulates**	**-0.13**	**[-0.23, -0.03]**	**0.99**	**
	**Size*Scent**	**-0.08**	**[-0.18, 0.01]**	**0.96**	*
	**Size*Availabiltiy**	**-0.04**	**[-0.09, 0.01]**	**0.95**	*
	**Size**	**-0.50**	**[-0.94, -0.07]**	**0.99**	**
	Moon (Full)	0.07	[-0.27, 0.39]	0.65	
	**Month (February)**	**0.77**	**[0.43, 1.11]**	**1.00**	**
	Ungulate (Yes)	-0.22	[-0.96, 0.47]	0.73	
	Scent (Yes)	0.20	[-0.53, 0.93]	0.72	
**Acorn removal**	**Availability**	**0.29**	**[0.12, 0.46]**	**1.00**	**
	**Size*Moon**	**0.29**	**[-0.02, 0.60]**	**0.96**	*
	Size*Month	-0.09	[-0.41, 0.22]	0.71	
	**Size*Ungulates**	**0.24**	**[-0.07, 0.55]**	**0.94**	•
	**Size*Scent**	**0.30**	**[0.00, 0.59]**	**0.98**	*
	Size*Availabiltiy	0.10	[-0.07, 0.26]	0.88	

Mean of posterior distribution, highest posterior density interval (HPD) and percentage of the posterior distribution with the same sign as the mean (f) are shown. Effects with f ≥ 0.95 are in bold. • depicts f $\in [0.90, 0.95)$

Table 2. Summary table of the effects of size, moonlight, month, ungulate presence, predator scent and local acorn availability (and their interactions with size) on acorn mobilization distances and the probability that it is deposited in a viable status (vs predated). A total of 211 acorns (mobilized outside cages and retrieved) were analyzed.

Process	Fixed effect	Mean	HPD	F	
**Mobilization distance**	Size	0.16	[-0.51, 0.83]	0.68	
	**Moon (Full)**	**-0.67**	**[-1.27, -0.06]**	**0.98**	*
	**Month (February)**	**0.54**	**[-0.14, 1.2]**	**0.94**	·
	**Ungulate (Yes)**	**-0.75**	**[-1.59, 0.14]**	**0.95**	*
	Scent (Yes)	0.09	[-0.73, 0.98]	0.57	
	Availability	-0.01	[-0.31, 0.29]	0.52	
	Size*Moon	-0.07	[-0.66, 0.49]	0.60	
	Size*Month	-0.33	[-0.94, 0.28]	0.86	
	Size*Ungulates	0.18	[-0.44, 0.81]	0.71	
	Size*Scent	0.22	[-0.36, 0.79]	0.78	
	Size*Availabiltiy	-0.16	[-0.49, 0.17]	0.83	
**Viability after deposition**	**Size**	**-1.20**	**[-2.15, -0.33]**	**1**	*
	Moon (Full)	0.42	[-0.38. 1.22]	0.85	
	**Month (February)**	**-1.58**	**[-2.46, -0.75]**	**1**	*
	**Ungulate (Yes)**	**0.67**	**[-0.32, 1.69]**	**0.91**	·
	Scent (Yes)	-0.16	[-1.14, 0.80]	0.63	
	**Availability**	**0.52**	**[0.10, 0.97]**	**0.99**	*
	**Size*Moon**	**0.66**	**[-0.12, 1.46]**	**0.95**	*
	Size*Month	0.49	[-0.33, 1.34]	0.88	
	**Size*Ungulates**	**0.59**	**[-0.22, 1.40]**	**0.92**	·
	Size*Scent	0.21	[-0.52, 0.94]	0.72	
	Size*Availabiltiy	-0.24	[-0.73, 0.25]	0.84	

Mean of posterior distribution, highest posterior density interval (HPD) and percentage of the posterior distribution with the same sign as the mean (f) are shown. Effects with f ≥ 0.95 are in bold. • depicts f $\in [0.90, 0.95)$

Figure legends

Fig. 1. Mouse foraging decisions during acorn selection and removal (upper and lower panels, respectively). Size of acorns (selected or not) in the presence or absence of (A) ungulates and (B) predator scent. Size of acorns (removed or not) (C) under new or full moon conditions and (D) in the presence or absence of predator scent. Point colors depict whether the acorn was selected or removed (yes, blue) or not (no, orange). In all cases acorn size is expressed in grams. Points represent mean values, bars standard errors (N=1677 foraging events).

Fig. 2. Mouse foraging decisions during transportation and after deposition (upper and lower panels, respectively). Mobilization distances of removed acorns under (A) new and full moon conditions and (B) in the absence and presence of ungulates. (C) Seed size effects on the probability of predation after deposition (black line represents mean effects and shaded area 0.95 credible intervals). (D) Proportion of acorns escaping predation after deposition in the absence and presence of ungulates. A total of 211 acorns were analyzed.

Fig. 3. Results from simulations of the probability transition model for acorn dispersal. (A) Proportion of acorns escaping predation in the presence or absence of environmental stressors (i.e. full moon, ungulates present or predator scent, black bar) in comparison to more optimal conditions (i.e. new moon, ungulates absent, no predator scent, grey bar). Size of predated (yellow) and viable (blue) acorns under (B) new *vs* full moon conditions and in the presence or absence of (C) ungulates and (D) predator scent. Bars represent mean values (±s.e.) across 1000 simulations.

Fig. 1

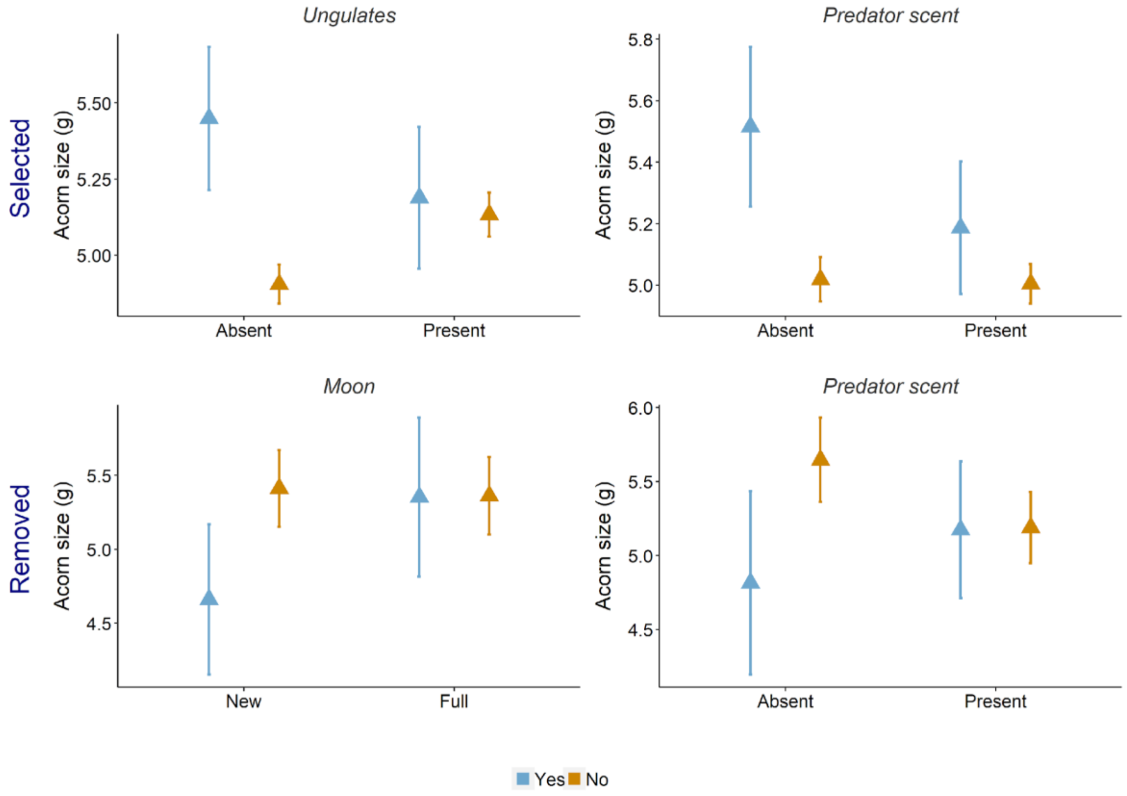

Ungulates
Predator scent
Selected
Acorn size (g)
5.50
5.25
5.00
Absent
Present
Acorn size (g)
5.8
5.6
5.4
5.2
5.0
Absent
Present
Moon
Predator scent
Removed
Acorn size (g)
5.5
5.0
4.5
New
Full
Acorn size (g)
6.0
5.5
5.0
4.5
Absent
Present
Yes
No

Fig. 2

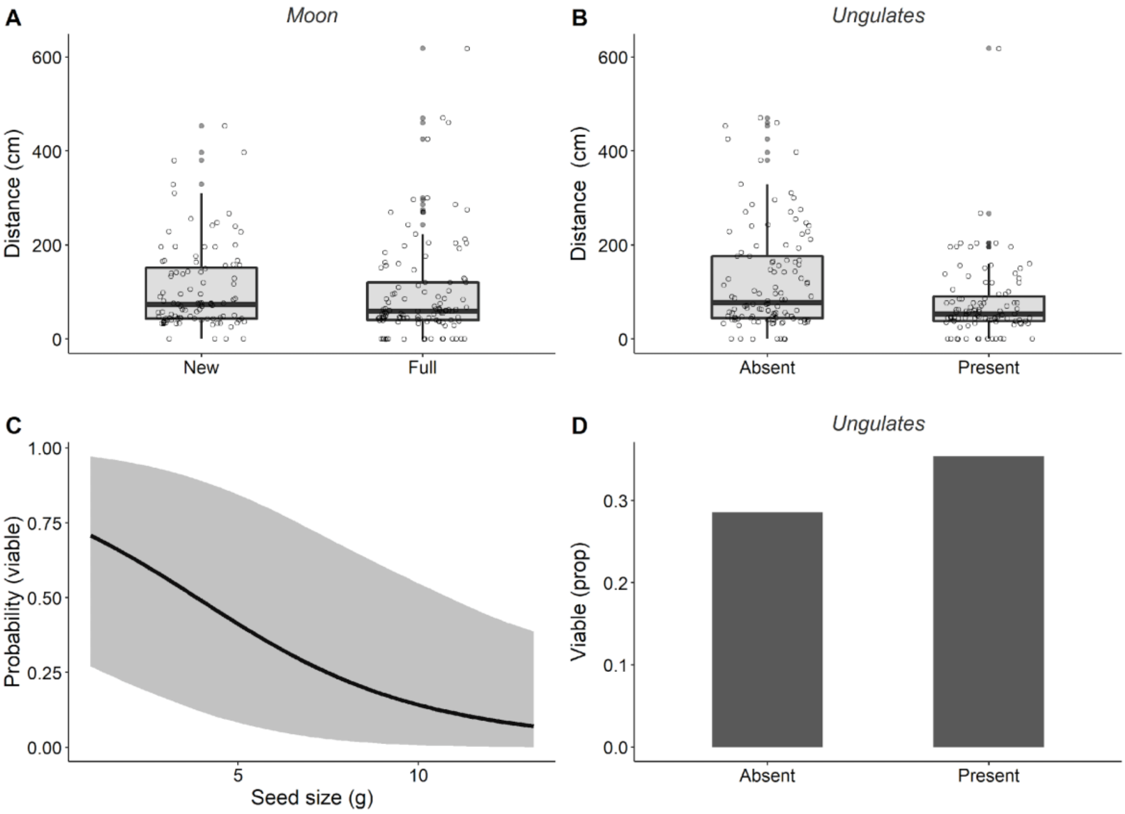
A
Moon
Distance (cm)
600
400
200
0
New
Full
B
Ungulates
Distance (cm)
600
400
200
0
Absent
Present
C
Probability (viable)
1.00
0.75
0.50
0.25
0.00
5
10
Seed size (g)
D
Ungulates
Viable (prop)
0.3
0.2
0.1
0.0
Absent
Present

Fig. 3

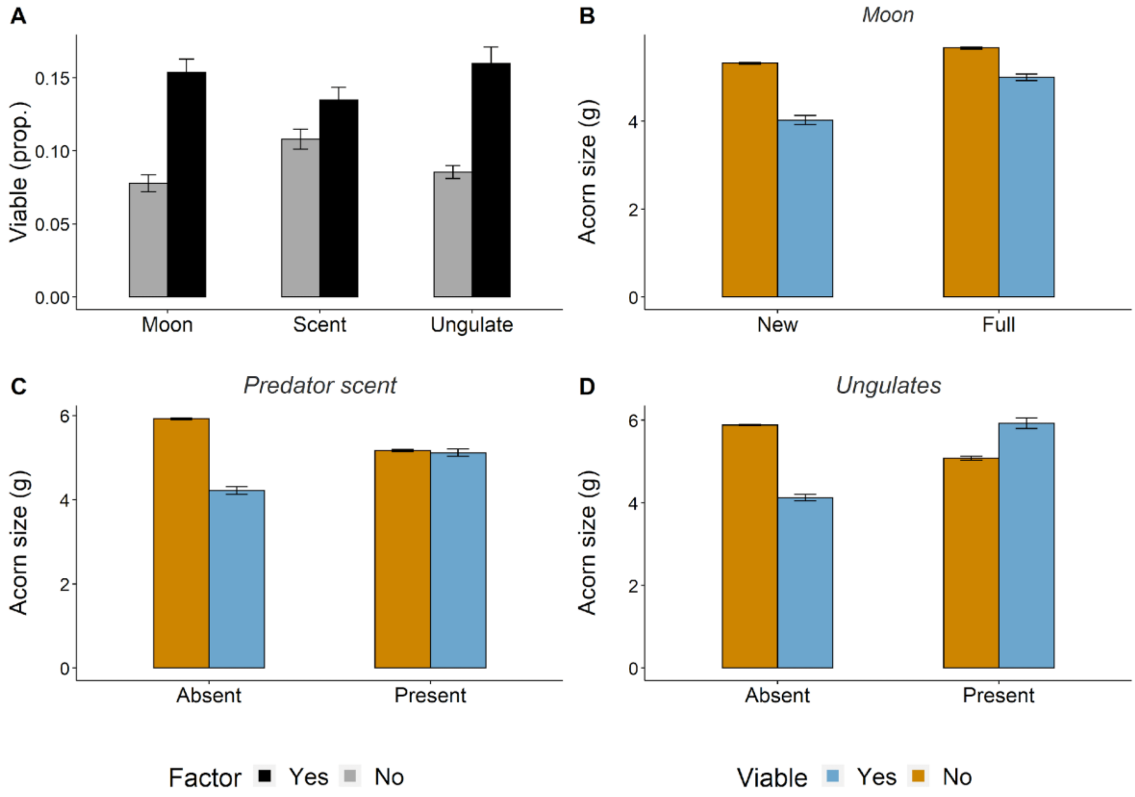
A
Viable (prop.)
0.15
0.10
0.05
0.00
Moon
Scent
Ungulate
B
Moon
Acorn size (g)
4
2
0
New
Full
C
Predator scent
Acorn size (g)
6
4
2
0
Absent
Present
D
Ungulates
Acorn size (g)
6
4
2
0
Absent
Present
Factor Yes No
Viable Yes No

Reviewer #1:

This study uses an experimental approach to investigate how the exclusion of ungulates and the presence of predator cues affects the removal, transportation, and short-term fate of Quercus ilex acorns handled by Algerian mice. The results show that the mice preferred large acorns for immediate consumption, but that this selectivity was changed by the presence of ungulates or predator cues, as well as the availability of more acorns. Acorns that were removed for caching were generally smaller, but this preference was changed in experiments with predator scents or ungulates present. Caching distances were generally very short and further reduced by the presence of full moon and outside of ungulate exclosures. Finally, the immediate post-dispersal predation of acorns was determined by acorn size, timing, and the presence of ungulates.

Based on their findings, the authors discuss how "environmental stress", exerted by potential competition with ungulates and predation pressure, simulated by scents, moves the mouse-oak interaction from the predation end of the seed fate spectrum towards mutualism. This interpretation is hard to follow, based on the presented data, as the differences in "dispersal" distance (maybe a mean difference of 10cm) are biologically irrelevant and the time span over which post-dispersal predation is monitored does not suffice to make inferences about its effects on seed fate that leads to plant establishment. Therefore, I'd suggest to focus the discussion more on the decisions the mice face and how these are influenced in the experiment, rather than suggesting that this affects the whole ecosystem. Likely, the most important drivers are the presence of shrub cover, water availability/drought, and herbivory by large ungulates. Nonetheless, the experiment is interesting in teaching us about decisions that rodents make when handling seeds under different conditions. Therefore, I suggest the authors shift the discussion towards the behavioral ecology of the rodents, and away from effects of competition and predation on dispersal effectiveness.

Thanks for the support of our work and remarking potential limitations of our approach. Your suggestions and concerns have been very valuable to tone down some parts of the text, making our introduction and discussion more robust. Regarding mobilization distances, in the absence of ungulates, mobilization distances were on average 0.50 m higher. In the case of seed fate, analysing the effects of acorn size and environmental factors on long-term post-dispersal survival would have needed between 7000 and 15000 tagged acorns (according to rates of long-term survival of 1-3%, see below). To connect all foraging decisions by mice (from selection to caching) we needed to monitor their behaviour, and hence, analysing such large number of videos would be very resource demanding and we did not have such large budgets. Most importantly, in the presence of ungulates (outside exclosures), it is very difficult to disentangle acorn retrieval by rodents from consumption by ungulates in the long term (from autumn to spring). Given these logistical constraints, we could not include this stage in our analyses. Though these limitations are common in studies evaluating the role of scatter-hoarding rodents, we agree that we should be more cautious when extrapolating our results. Therefore, as suggested, we have tried to strengthen the focus of the MS on the

effects of our experimental manipulations on the scatter-hoarding behaviour of mice and its likely biological causes; though, softening and reducing the sentences more directly concerned to the 'plant side' of the interaction. Nevertheless, we cannot eliminate completely discussion on the consequences of our results for the conditional mutualism between mice and oaks. In this work we analyse how and why competition and risk can affect initial seed fates, which have been found to be important for recruitment in our study system (Pulido and Díaz 2005, Pulido et al. 2010, Muñoz and Bonal 2011, Díaz et al. 2021). For this reason, we keep some sentences regarding consequences of the observed responses for the conditional mutualism, although moving them to the end of the discussion and acknowledging what further work is needed to fully understand, and parameterize, factors influencing oak recruitment.

L48: "seed dispersers" – otherwise the readers may think of natal or breeding dispersal of the hoarders themselves. Generally, please make sure to refer to "seed dispersal", rather than simply "dispersal" (eg L65) for clarity

Changed accordingly. We have also revised the text and specified that we are referring to seed dispersal (e.g. L24, 42, 49)

L48: To avoid the awkward term "acorn-bearing trees", you could rephrase to "Scatter-hoarders are key seed dispersers in temperate and Mediterranean forests dominated by oaks [1-5]"

Thank you, changed accordingly (L50).

L56: "...space and time, making it difficult to predict..." (add comma and "it")

Added (L57).

L58: here and throughout: unless referring specifically to multiple individuals, please consider using the singular "mouse" rather than "mice" (e.g. "oak-mouse" in abstract etc).

Thank you, I always have problems with this part of the English grammar. Revised and changed accordingly throughout the text (e.g. L27, 62, 89).

L61: Please explain to the reader why competition encourages seed mobilization (just saw it on L66; consider moving that part forward a bit)

Indeed, it was not clear enough. Scatter-hoarding can ensure a more rapid acquisition of food for the same amount of time. Predating seeds *in situ* tends to be more time-consuming that mobilizing them for later consumption (at least over short distances). Therefore, when competition is high, a more efficient strategy is mobilizing and storing seeds before they are consumed by other individuals. This is now specified in L67-69

L58-72: While I understand the need to keep it short, it seems that the dynamics that turn a potential seed predation event into a seed dispersal event are oversimplified. Transportation distance alone does not make the interaction more antagonistic or mutualistic. While I know that not all aspects of seed fate can easily be quantified, it

should at least be noted here that not only seed transport, but also consumption after caching can result in a seed predation event.

We agree that to really measure dispersal effectiveness we need to evaluate at least long-term acorn survival. Also, that we cannot ensure that cached acorns are not predated throughout the following weeks. In fact, survival rates tend to drop one order of magnitude between autumn and the following spring (e.g. from 36 to 3.4% of cache survival Perea 2011). The main reason why most studies evaluating acorn dispersal by rodents do not model long-term survival is that these rates are very low (1-3%, Gómez 2008, Perea et al. 2011). This limits sample sizes with affordable sampling designs. For instance, to have enough sample size for our models (N>200), we would have needed to track between 7000 and 20000 acorns. A way to circumvent this problem is performing seed sowing experiments, where we can control the initial sample size. This approach provides very valuable information about microhabitat effects on post-dispersal predation rates, emergence and initial survival, but they are not real caches. Thus, post-dispersal recovery by cache owners cannot be estimated. In sum, problems raised in this work are typical of studies aimed at evaluating the role scatter-hoarding rodents, where high removal rates but low dispersal efficiency makes it difficult to estimate their net effect. This is why other proxies like mobilization distances or initial fates are used.

Having said so, we agree with your concerns, and hence, we have toned down some parts of our work. We have rewritten this sentence (L75) and included these issues in the discussion (L350-371).

L123: please provide latin species name for the Algerian mouse

Changed accordingly L131.

L127: do the mice live in or below the trees? (ie are they arboreal?) Consider rephrasing to "Mouse occupancy below target trees was established..."

Algerian mice live underground below trees. They can climb occasionally but they do not live in trees. That's the reason for our statement, that we thus maintain.

L136: "consisted of..."

Changed accordingly L145.

L151: For how many days did you search? This is really important for your definition of viability. Consider the fact that cached acorns may be retrieved and consumed weeks later.

Acorns were searched and relocated at 24 and 72 hours. Mobilized and not predated acorns were tracked throughout the experiment (which lasted until spring). However, outside exclosures, in the long term, when a mobilized acorn disappeared it was very difficult to tease apart predation by ungulates from that of cache owners of pilferers. This is the main reason that prevented us from analysing final fates. Now explained in L162-166.

L179: "acorn availability (g)" is this the natural crop of the tree or the overall mass of acorns provided to the mice during the experiment?

It is the overall mass of acorns provided to the mice during the experiment. In methods we say that any acorn present in the area where cages were placed was removed (in November, as there were no acorns left in February). Hence, we think that it is not necessary to modify the sentence to remark this here.

L182: scaling: please describe how and why

Thank you, acorn size and availability were standardized (mean= 0, sd= 1) so that the magnitude of covariate effects could be compared (L196). Also, it facilitates parameter estimation making MCMC sampling inefficient.

L183: "..intercept term to account for repeated sampling."

Added accordingly L198.

Figures: Consider using "predator scent" to label the plots. Also, please add sample sizes to captions.

Good point, changed accordingly.

Figures 1 & 3: Does the color add any information? Otherwise consider making all figures black and white and differentiating yes/no with symbols or grayscale colors (eg Fig 3A)

In figure 1 and 3 blue and orange depicts whether the acorn continues the process of seed dispersal (that is selected, removed and deposited without being predated). Grey and black scale depicts whether the environmental stressor was present or not (in Fig. 3A). We have chosen these colours because they are colour-blind friendly.

L252: The last sentence reads as if it contradicts itself (more mobilization when no food, but more when lots of food). Consider rephrasing

It has been rewritten, hopefully it is clearer now L267-269.

L274: "environmental stress" is a very broad and loaded term, which could mean temperatures, etc. Please be concise

Done, thanks. We have changed 'environmental stress' by 'competition and predation risk' or equivalent to avoid a too vague term.

L280: "....probability to survive the first days after caching." Since the whole point of caching is subsequent consumption, which may occur long after caching, I would be careful with this interpretation. However, by providing the time period over which you monitored seed fate, you can make this statement more accurate.

We have specified that the we are evaluating seed fate in the first days (L162). Our impression is that final fates did not change much after dispersal outside canopies (as

suggested by experiments carried out in the study area some years before the experiment reported here; Smit et al. 2008, 2009, Muñoz and Bonal 2011), but we have no hard data to support this impression. Therefore, we have toned downed this paragraph.

L282: Please expand on this notion of intermediate stress. The reference you provide in the introduction, Lichti et al. Biol Rev, only discusses the effect of intermediate seed availability on seed dispersal by rodents, but not overall stress. In the context of your study, you only compared the presence and absence of putative stressors, thus not providing quantitative support for the role of "intermediate stress". Also, the argument that the recovery of cached seeds is affected slightly misses the point. Isn't it about the moving of seeds in the first place? Why would the mouse take the risk to cache under high predation pressure, but then avoid that risk during recovery? In general, I think the "viability" argumentation is very limited by the time period over which the transported acorns were monitored (a few days, I assume).

In this work we are referring to two main stressors: predation risks and competition for seeds. In the former case (section 9 of Lichi et al's work), animals may preferentially mobilize seeds to reduce handling times, which are much lower when seeds are transported over short distances than consumed *in situ* (section 4). Also, they may transport seeds towards safer areas for later consumption. In fact, recent work has shown that under predation risks, individuals suffering higher stress levels (measured by their behavioral responses) are also those mobilizing seeds further (Feldman et al. 2019, https://doi.org/10.1016/j.anbehav.2019.02.009). Regarding competition, foraging decisions are modulated by handling times and missed opportunity costs (section 5). These issues are introduced in the following paragraph. Nonetheless, we agree that it is difficult to define "intermediate stress", and hence, we avoid to use this general term throughout the text and refer to suboptimal conditions for mice, or we specify that we are referring to competition and risk.

Regarding viability, we monitored acorns during three days and continued monitoring caches during the study period. However, it was difficult to obtain reliable data from this long-term monitoring. Acorn consumption by ungulates outside exclosures made it difficult to tease apart decisions made by rodents from those related to consumption by other animals. Even though our results are limited in this short time lapse, seed viability short after deposition is a necessary condition for seedling establishment. Therefore, differences here found can have an imprint on oak recruitment provided that they are outweighed by other post-dispersal processes that differ mainly between habitats outside and inside exclosures (e.g. long-term cache survival, emergence …). Having said so, we agree that it is a limitation of our work that we now discuss (L350-371) and toned down some parts of the text.

*L300: "Risk rather than competition modulates the effect of ungulate presence on acorn selection". It seems to me that table 1 shows the opposite. The effect size of ungulate presence (size * ungulate) is nearly twice that of size*scent. Why wasn't the interaction scent * ungulate included? It seems the experiment would be optimal to test the interaction between the two putative drivers of acorn selection.*

In this sentence we are referring to the ecological interpretation of the negative effect size*ungulates. We expected that in the presence of competitors mice would be more selective, preferentially foraging on the most valuable items before they are consumed by others. In other words, we were expecting a positive size*ungulate effect. Nonetheless, we found the opposite. When ungulates were present, mice were not selective at all. Our interpretation is that outside ungulate exclosures, lack of antipredatory cover in the surroundings of dehesa trees increase risk. We have rewritten the last sentence to make our point clearer (L313-316).

The decision of not including scent*ungulate interaction was something we discussed a lot previous to data analyses. We did not include interactions between environmental variables due to sample size limitations; especially in stages of acorn mobilization and initial fates. Including interactions between environmental variables would result in a high number of parameters to be estimated. Therefore, we decided to prioritize interactions between size and environmental variables in our data analyses. Size*environment interactions inform about foraging strategies prioritizing food acquisition *vs* safety, allowing us to evaluate the underlying reasons of changes in seed dispersal patterns.

L307: rephrase, hard to follow

Rewritten, L321-323.

Reviewer #2:

The study has a very interesting aim which is to explore whether biological integrity of the system can have positive effects on scatter hoarding and thus regeneration of the keystone oak. Results are generally nicely written-up, but figures need revisions. I have some comments regarding presentation, with the hope that these revisions can make the reading of the study easier.

We are grateful for the support to our work. Concerns raised during this revision have helped us to improve the clarity of our figures, to provide information more transparently and to better explain figure legends. We hope all issues have been solved.

Well explained experimental design – 40 trees, 10 assigned to a combination of herbivore exclusion crossed with genet scent addition.

Thank you.

Data analysis: sample size for the analysis needs to be provided, as it appears like model can be overfitted: 6 fixed effects + many interactions.

This is important. In total, we offered 2280acorns. However, the total number of foraging events analysed for selection and removal decisions were 1677. Out of the 1677 acorns handled, 267 were mobilized outside the cages and 79% were retrieved. This information is now provided in Table 1 and 2, figure legends and in the results section L255-256.

This is an appropriate sample size for our analysis, as shown in our Rhat estimations (<1.1) and posterior predictive checks (Supplementary material Fig. S2_1 to Fig. S2_4).

I am not familiar with Bayesian framework, so pardon the question. How the effects can be considered meaningful (or significant, but it is not about semantics) if their 95% CI overlap zero?

Not a problem. The “f” parameter (last column of tables) measures the proportion of the posterior distribution that has the same sign of the posterior mean. This would be comparable to the P value with an estimation with maximum likelihood. When a f value is >= 0.95 it means that there is a probability that the posterior distribution has the same sign of the mean. It is equivalent somehow to $\alpha = 0.05$, in maximum-likelihood estimations. This is the reason why we indicate with * factors with an f value >0.95 and with a point those with f [0.90, 0.95]. Highest posterior density intervals (HPD) are built with quantiles 0.025 and 0.975, therefore, it will overlap 0 whenever f<0.975. Even when this happens, we can be quite sure that there is an effect if f is large (e.g. >=0.95).

Figures needs major revisions. First, all figure captions should explain what is being showed, not provide the interpretation.

As suggested, we have changed figure captions so that they provide a description of what is represented rather than an interpretation.

The current version of Fig 1 is difficult to interpret, and the figure caption does not help as instead explaining what is shown, it presents the interpretation. Acorn size was categorial here with three levels, yet only two are shown. Then, if the acorn size was category, what is the point of showing it at y-axis which should be the place of response variable

We believe that there is a misunderstanding with figure 1, probably because the legend was not clear enough and labels of x-axis could be confused with colour code. Fig 1 represents the size of acorns that were selected (or not) and removed (or not), under different environmental (i.e. presence/absence of ungulates, presence/absence predator scent and new/full moon). Colour code does not represent levels of acorn size, but mouse foraging decisions. Representing size in the y-axis, environmental conditions in the x-axis and foraging decisions (yes/no) in the colour code, allowed us to represent the interaction between acorn size and these environmental factors on mouse foraging choices. Figure 1 shows that acorn size only differed between selected and not selected acorns under "low stress conditions" (no ungulates, no predator scent). The same applies for removal. The difference between selection and removal is that though mice preferentially selected large acorns they tended to remove smaller ones.

We have rearranged the figure to avoid confusions between "yes/no" in the color code (foraging decisions) and in the x-axis (environmental conditions). Now for environmental conditions we refer to present/absent in the case of ungulates and predator scent; and new/full moon conditions. In addition, we have rewritten the figure legend and we hope it is clearer now.

– I guess we are here mostly interested in probabilities? I suggest that all panels at Figure 1 show Probability at y-axis, acorns size at x-axis, and how the probability of each process changes with size depending on the treatment (ungulates, scent, moon etc).

To represent probabilities, we need sample posterior distributions and for each acorn calculate the probability of selection and removal. Then, repeat the procedure N number of iterations and obtain a mean value per acorn. This would be a little bit circular with respect to the model because instead of showing raw values (as in the current figure 1), model predictions would be represented.

We have made a figure of probabilities; in case you feel it is absolutely necessarily to change it. However, we would prefer not to for two reasons. The first one, is that simulations are made with model estimates, and hence, the reader cannot evaluate how well model estimates reflect raw data. The second one, is that in the case of acorn selection, boxplots are very noisy. Acorn selection is modelled following a multinomial regression, and hence, probabilities not only depend on acorn size and environmental covariates; but also, on the number of acorns available. For instance, with no selection, when 15 acorns are available each one will have a probability of 0.067; whereas when 4 acorns are available it will be of 0.25. These simulations cannot tease apart such effects.

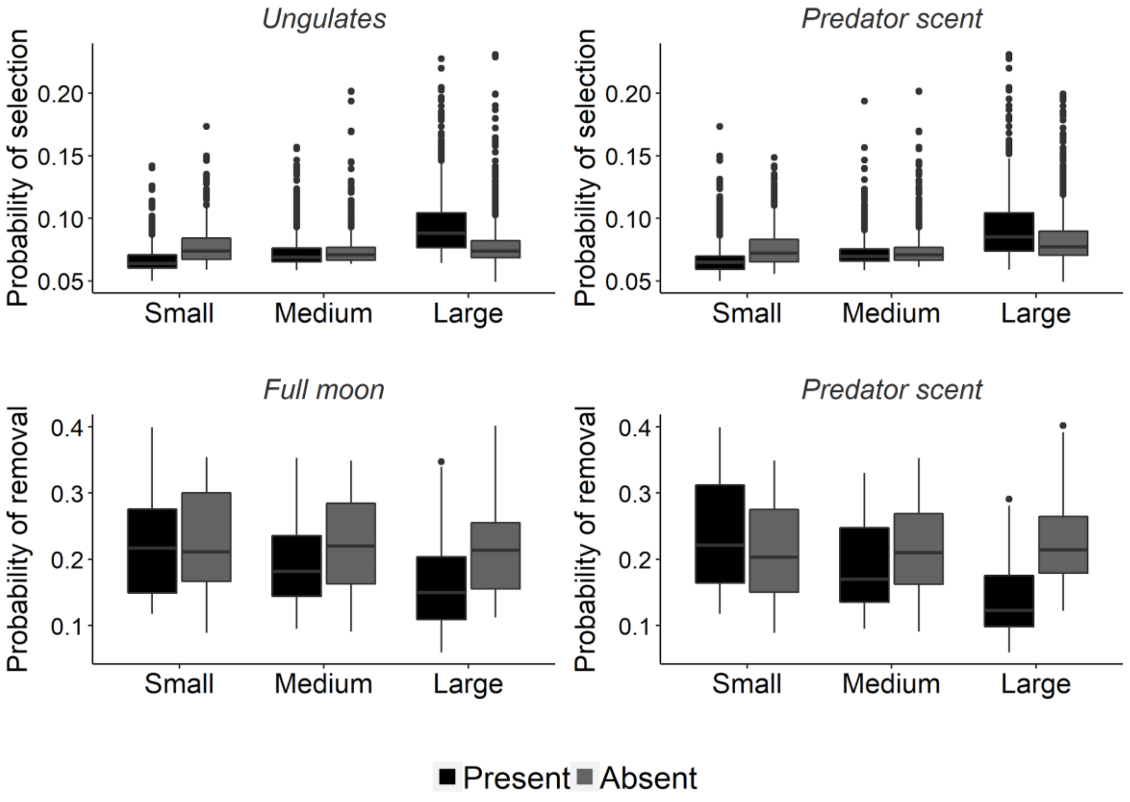

Fig. R1. Probability of acorn selection (upper panels) and removal (lower panels) for small (<3 g), medium ([3,6] g) and large (>6 g) acorns. Colour code represents whether the stressor (ungulates, predator scent and full moon, was present or not). For each acorn and foraging event, we estimated the probability of selection/removal by sampling 5000 iterations of posterior distributions. Then, we calculated mean values across iterations, with which we represented boxplots.

Boxplots at Fig 2 should include data points in the background.

Good point, added accordingly. Also, as suggested, we have rewritten the figure legend to make it clearer.

L50: The net outcome of the interaction does not depend on whether seeds are consumed or cached, as seeds are usually both consumed and cached in each interaction. The key is the balance between predation and dispersal, and the balance of the benefit (improved recruitment) vs cost (predation and thus reduced recruitment).

Right! For simplicity, the sentence now reads ‘The outcome of the interaction may be either mutualistic (dispersal) or antagonistic (predation) depending on the proportion of seeds consumed vs. cached and not retrieved [6]. (L51). We did not expand the sentence to include other components of benefits (i.e. the longer-term survival prospects of seedlings depending on microsite conditions) because these issues, currently well developed for mutualisms such as dispersal of fleshy-fruited plants by frugivores, are much less developed for synzoochorous interactions (review by Gómez et al. 2019). In

fact, these authors remark in their review on nut dispersal effectiveness that data available refers to relatively short-term nut survival, not overall plant recruitment.

L58: This sentence suggests that corvids are inefficient dispersers in oaks savannas, yet we have papers showing the contrary both in dehesas (Baroja et al: https://besjournals.onlinelibrary.wiley.com/doi/abs/10.1111/1365-2745.13642) as well as in oak savannas in other regions (e.g. Pesendorfer et al, papers from California).

Indeed, corvids can be important dispersers in open areas (e.g. magpies). We have reformulated the beginning of this paragraph accordingly (L59-61).

L70: "In the absence of stress… " this sentence is oversimplification and sounds like rodents never store seeds in the absence of stress, which is not true. Simply put, if there is lots of food (no stress) we do expect that rodents will start to store.

Thanks for the remark. We have modified the sentence that now reads 'In the absence of stress, rodents usually act as efficient seed predators as they consume, immediately or soon afterwards, most seed crops under the canopy of mother trees' L77-79.

L112: what does it mean that they were opened?

As quoted in the preceding sentence, 'Dehesas are savanna-like man-made habitats resulting from shrub removal and tree thinning and pruning to enhance herb growth for livestock [41].' L115-117. This implicitly means that they should be 'opened' from closed forests. Now the sentence reads 'The studied dehesas were opened from the original Mediterranean forests in the late 1950s'

L121: averages of both areas are needed to support a statement that they have similar tree abundance

We intended to mean that tree densities were the same inside and outside each exclosure, not that tree density was uniform across the study area. Now we provide mean and SE values for tree density (No./ha) around the 50 focal trees in each site x exclosure combination where we trapped mice. Overall mean was 20.4 trees/ha, but site 1 has 30 trees/ha both inside and outside the exclosure and site 2, 7 trees/ha. The sentence now reads 'Average tree density was 20.4 trees ha-1, although site 1 had higher mean density than site 2 (30.0±2.6 and 30.1±3.4 inside and outside ungulate exclosure in site 1; and 7.4±0.4 and 7.4±0.3 in site 2; mean±SE). Shrub cover was <1%, as measured on aerial photographs and vegetation surveys both under canopies and outside them [24].' L126-131.

Reviewer #3: This is a comprehensive study as to the joint effect of habitat structure, competition and predation risk on dispersal effectiveness in an oak-mice system. They found that intermediate stress (presence of predator or grazer) could increase dispersal effectiveness and then facilitated interaction towards the mutualistic side, providing new evidences on conditional mutualism. I think this is a good contribution to the study of the field. I have only a few revision suggestions, mainly by including a few previous similar studies:

We are grateful for the support to our work.

Line: 147-148. The wire-linked plastic seed tagging method was proposed by Xiao et al. (2006). You need to include the original reference: Xiao et al, 2006, Forest Ecology and Management, 223:18–23 ；

Changed accordingly, L158.

Line 296-299. You found ungulate presence would benefit dispersal of oak acorns. A previous study had similar results. You should include the reference in the discussion: Zhang et al. (2009), Wildlife Research, 36: 610–616;

Thank you, we had missed this important reference. It now included L315.
