# Supplementary material for: Ungulate presence and predation risks reduce acorn predation by mice in dehesas
Source: PLoS One. 2022 Aug 15;17(8):e0260419. doi: 10.1371/journal.pone.0260419 (PMC9377575; doi:10.1371/journal.pone.0260419)
Supplement: Attachment — Submitted filename: MoranLopez_etal_BiolIntegrity_Response_to_Reviewers.docx [file pone.0260419.s007.docx]

*Reviewer #1:*

*This study uses an experimental approach to investigate how the exclusion of ungulates and the presence of predator cues affects the removal, transportation, and short-term fate of Quercus ilex acorns handled by Algerian mice. The results show that the mice preferred large acorns for immediate consumption, but that this selectivity was changed by the presence of ungulates or predator cues, as well as the availability of more acorns. Acorns that were removed for caching were generally smaller, but this preference was changed in experiments with predator scents or ungulates present. Caching distances were generally very short and further reduced by the presence of full moon and outside of ungulate exclosures. Finally, the immediate post-dispersal predation of acorns was determined by acorn size, timing, and the presence of ungulates.*

*Based on their findings, the authors discuss how “environmental stress”, exerted by potential competition with ungulates and predation pressure, simulated by scents, moves the mouse-oak interaction from the predation end of the seed fate spectrum towards mutualism. This interpretation is hard to follow, based on the presented data, as the differences in “dispersal” distance (maybe a mean difference of 10cm) are biologically irrelevant and the time span over which post-dispersal predation is monitored does not suffice to make inferences about its effects on seed fate that leads to plant establishment. Therefore, I’d suggest to focus the discussion more on the decisions the mice face and how these are influenced in the experiment, rather than suggesting that this affects the whole ecosystem. Likely, the most important drivers are the presence of shrub cover, water availability/drought, and herbivory by large ungulates. Nonetheless, the experiment is interesting in teaching us about decisions that rodents make when handling seeds under different conditions. Therefore, I suggest the authors shift the discussion towards the behavioral ecology of the rodents, and away from effects of competition and predation on dispersal effectiveness.*

Thanks for the support of our work and remarking potential limitations of our approach. Your suggestions and concerns have been very valuable to tone down some parts of the text, making our introduction and discussion more robust. Regarding mobilization distances, in the absence of ungulates, mobilization distances were on average 0.50 m higher. In the case of seed fate, analysing the effects of acorn size and environmental factors on long-term post-dispersal survival would have needed between 7000 and 15000 tagged acorns (according to rates of long-term survival of 1-3%, see below). To connect all foraging decisions by mice (from selection to caching) we needed to monitor their behaviour, and hence, analysing such large number of videos would be very resource demanding and we did not have such large budgets. Most importantly, in the presence of ungulates (outside exclosures), it is very difficult to disentangle acorn retrieval by rodents from consumption by ungulates in the long term (from autumn to spring). Given these logistical constraints, we could not include this stage in our analyses. Though these limitations are common in studies evaluating the role of scatter-hoarding rodents, we agree that we should be more cautious when extrapolating our results. Therefore, as suggested, we have tried to strengthen the focus of the MS on the effects of our experimental manipulations on the scatter-hoarding behaviour of mice and its likely biological causes; though, softening and reducing the sentences more directly concerned to the ‘plant side’ of the interaction. Nevertheless, we cannot eliminate completely discussion on the consequences of our results for the conditional mutualism between mice and oaks. In this work we analyse how and why competition and risk can affect initial seed fates, which have been found to be important for recruitment in our study system (Pulido and Díaz 2005, Pulido et al. 2010, Muñoz and Bonal 2011, Díaz et al. 2021). For this reason, we keep some sentences regarding consequences of the observed responses for the conditional mutualism, although moving them to the end of the discussion and acknowledging what further work is needed to fully understand, and parameterize, factors influencing oak recruitment.

*L48: “seed dispersers” – otherwise the readers may think of natal or breeding dispersal of the hoarders themselves. Generally, please make sure to refer to “seed dispersal”, rather than simply “dispersal” (eg L65) for clarity*

Changed accordingly. We have also revised the text and specified that we are referring to seed dispersal (e.g. L24, 42, 49)

*L48: To avoid the awkward term “acorn-bearing trees”, you could rephrase to “**Scatter-hoarders are key seed dispersers in temperate and Mediterranean forests dominated by oaks [1-5]”*

Thank you, changed accordingly (L50).

*L56: “...space and time, making it difficult to predict...” (add comma and “it”)*

Added (L57).

*L58: here and throughout: unless referring specifically to multiple individuals, please consider using the singular “mouse” rather than “mice” (e.g. “oak-mouse” in abstract etc).*

Thank you, I always have problems with this part of the English grammar. Revised and changed accordingly throughout the text (e.g. L27, 62, 89).

*L61: Please explain to the reader why competition encourages seed mobilization (just saw it on L66; consider moving that part forward a bit)*

Indeed, it was not clear enough. Scatter-hoarding can ensure a more rapid acquisition of food for the same amount of time. Predating seeds *in situ* tends to be more time-consuming that mobilizing them for later consumption (at least over short distances). Therefore, when competition is high, a more efficient strategy is mobilizing and storing seeds before they are consumed by other individuals. This is now specified in L67-69

*L58-72: While I understand the need to keep it short, it seems that the dynamics that turn a potential seed predation event into a seed dispersal event are oversimplified. Transportation distance alone does not make the interaction more antagonistic or mutualistic. While I know that not all aspects of seed fate can easily be quantified, it should at least be noted here that not only seed transport, but also consumption after caching can result in a seed predation event.*

We agree that to really measure dispersal effectiveness we need to evaluate at least long-term acorn survival. Also, that we cannot ensure that cached acorns are not predated throughout the following weeks. In fact, survival rates tend to drop one order of magnitude between autumn and the following spring (e.g. from 36 to 3.4% of cache survival Perea 2011). The main reason why most studies evaluating acorn dispersal by rodents do not model long-term survival is that these rates are very low (1-3%, Gómez 2008, Perea et al. 2011). This limits sample sizes with affordable sampling designs. For instance, to have enough sample size for our models (N>200), we would have needed to track between 7000 and 20000 acorns. A way to circumvent this problem is performing seed sowing experiments, where we can control the initial sample size. This approach provides very valuable information about microhabitat effects on post-dispersal predation rates, emergence and initial survival, but they are not real caches. Thus, post-dispersal recovery by cache owners cannot be estimated. In sum, problems raised in this work are typical of studies aimed at evaluating the role scatter-hoarding rodents, where high removal rates but low dispersal efficiency makes it difficult to estimate their net effect. This is why other proxies like mobilization distances or initial fates are used.

Having said so, we agree with your concerns, and hence, we have toned down some parts of our work. We have rewritten this sentence (L75) and included these issues in the discussion (L350-371).

*L123: please provide latin species name for the Algerian mouse*

Changed accordingly L131.

*L127: do the mice live in or below the trees? (ie are they arboreal?) Consider rephrasing to “Mouse occupancy below target trees was established...”*

Algerian mice live underground below trees. They can climb occasionally but they do not live in trees. That's the reason for our statement, that we thus maintain.

*L136: “consisted of...”*

Changed accordingly L145.

*L151: For how many days did you search? This is really important for your definition of viability. Consider the fact that cached acorns may be retrieved and consumed weeks later.*

Acorns were searched and relocated at 24 and 72 hours. Mobilized and not predated acorns were tracked throughout the experiment (which lasted until spring). However, outside exclosures, in the long term, when a mobilized acorn disappeared it was very difficult to tease apart predation by ungulates from that of cache owners of pilferers. This is the main reason that prevented us from analysing final fates. Now explained in L162-166.

*L179: “acorn availability (g)” is this the natural crop of the tree or the overall mass of acorns provided to the mice during the experiment?*

It is the overall mass of acorns provided to the mice during the experiment. In methods we say that any acorn present in the area where cages were placed was removed (in November, as there were no acorns left in February). Hence, we think that it is not necessary to modify the sentence to remark this here.

*L182: scaling: please describe how and why*

Thank you, acorn size and availability were standardized (mean= 0, sd= 1) so that the magnitude of covariate effects could be compared (L196). Also, it facilitates parameter estimation making MCMC sampling inefficient.

*L183: “..intercept term to account for repeated sampling.”*

Added accordingly L198.

*Figures: Consider using “predator scent” to label the plots. Also, please add sample sizes to captions.*

Good point, changed accordingly.

*Figures 1 & 3: Does the color add any information? Otherwise consider making all figures black and white and differentiating yes/no with symbols or grayscale colors (eg Fig 3A)*

In figure 1 and 3 blue and orange depicts whether the acorn continues the process of seed dispersal (that is selected, removed and deposited without being predated). Grey and black scale depicts whether the environmental stressor was present or not (in Fig. 3A). We have chosen these colours because they are colour-blind friendly.

*L252: The last sentence reads as if it contradicts itself (more mobilization when no food, but more when lots of food). Consider rephrasing*

It has been rewritten, hopefully it is clearer now L267-269.

*L274: “environmental stress” is a very broad and loaded term, which could mean temperatures, etc. Please be concise*

Done, thanks. We have changed ‘environmental stress’ by ‘competition and predation risk’ or equivalent to avoid a too vague term.

*L280: “....probability to survive the first days after caching.” Since the whole point of caching is subsequent consumption, which may occur long after caching, I would be careful with this interpretation. However, by providing the time period over which you monitored seed fate, you can make this statement more accurate.*

We have specified that the we are evaluating seed fate in the first days (L162). Our impression is that final fates did not change much after dispersal outside canopies (as suggested by experiments carried out in the study area some years before the experiment reported here; Smit et al. 2008, 2009, Muñoz and Bonal 2011), but we have no hard data to support this impression. Therefore, we have toned downed this paragraph.

*L282: Please expand on this notion of intermediate stress. The reference you provide in the introduction, Lichti et al. Biol Rev, only discusses the effect of intermediate seed availability on seed dispersal by rodents, but not overall stress. In the context of your study, you only compared the presence and absence of putative stressors, thus not providing quantitative support for the role of “intermediate stress”. Also, the argument that the recovery of cached seeds is affected slightly misses the point. Isn’t it about the moving of seeds in the first place? Why would the mouse take the risk to cache under high predation pressure, but then avoid that risk during recovery? In general, I think the “viability” argumentation is very limited by the time period over which the transported acorns were monitored (a few days, I assume).*

In this work we are referring to two main stressors: predation risks and competition for seeds. In the former case (section 9 of Lichi et al’s work), animals may preferentially mobilize seeds to reduce handling times, which are much lower when seeds are transported over short distances than consumed *in situ* (section 4). Also, they may transport seeds towards safer areas for later consumption. In fact, recent work has shown that under predation risks, individuals suffering higher stress levels (measured by their behavioral responses) are also those mobilizing seeds further (Feldman et al. 2019, <https://doi.org/10.1016/j.anbehav.2019.02.009>). Regarding competition, foraging decisions are modulated by handling times and missed opportunity costs (section 5). These issues are introduced in the following paragraph. Nonetheless, we agree that it is difficult to define “intermediate stress”, and hence, we avoid to use this general term throughout the text and refer to suboptimal conditions for mice, or we specify that we are referring to competition and risk.

Regarding viability, we monitored acorns during three days and continued monitoring caches during the study period. However, it was difficult to obtain reliable data from this long-term monitoring. Acorn consumption by ungulates outside exclosures made it difficult to tease apart decisions made by rodents from those related to consumption by other animals. Even though our results are limited in this short time lapse, seed viability short after deposition is a necessary condition for seedling establishment. Therefore, differences here found can have an imprint on oak recruitment provided that they are outweighed by other post-dispersal processes that differ mainly between habitats outside and inside exclosures (e.g. long-term cache survival, emergence …). Having said so, we agree that it is a limitation of our work that we now discuss (L350-371) and toned down some parts of the text.

*L300: “Risk rather than competition modulates the effect of ungulate presence on acorn selection”. It seems to me that table 1 shows the opposite. The effect size of ungulate presence (size * ungulate) is nearly twice that of size*scent. Why wasn’t the interaction scent * ungulate included? It seems the experiment would be optimal to test the interaction between the two putative drivers of acorn selection.*

In this sentence we are referring to the ecological interpretation of the negative effect size*ungulates. We expected that in the presence of competitors mice would be more selective, preferentially foraging on the most valuable items before they are consumed by others. In other words, we were expecting a positive size*ungulate effect. Nonetheless, we found the opposite. When ungulates were present, mice were not selective at all. Our interpretation is that outside ungulate exclosures, lack of antipredatory cover in the surroundings of dehesa trees increase risk. We have rewritten the last sentence to make our point clearer (L313-316).

The decision of not including scent*ungulate interaction was something we discussed a lot previous to data analyses. We did not include interactions between environmental variables due to sample size limitations; especially in stages of acorn mobilization and initial fates. Including interactions between environmental variables would result in a high number of parameters to be estimated. Therefore, we decided to prioritize interactions between size and environmental variables in our data analyses. Size*environment interactions inform about foraging strategies prioritizing food acquisition *vs* safety, allowing us to evaluate the underlying reasons of changes in seed dispersal patterns.

*L307: rephrase, hard to follow*

Rewritten, L321-323.

*Reviewer #2:*

*The study has a very interesting aim which is to explore whether biological integrity of the system can have positive effects on scatter hoarding and thus regeneration of the keystone oak. Results are generally nicely written-up, but figures need revisions. I have some comments regarding presentation, with the hope that these revisions can make the reading of the study easier.*

We are grateful for the support to our work. Concerns raised during this revision have helped us to improve the clarity of our figures, to provide information more transparently and to better explain figure legends. We hope all issues have been solved.

*Well explained experimental design – 40 trees, 10 assigned to a combination of herbivore exclusion crossed with genet scent addition.*

Thank you.

*Data analysis: sample size for the analysis needs to be provided, as it appears like model can be overfitted: 6 fixed effects + many interactions.*

This is important. In total, we offered 2280acorns. However, the total number of foraging events analysed for selection and removal decisions were 1677. Out of the 1677 acorns handled, 267 were mobilized outside the cages and 79% were retrieved. This information is now provided in Table 1 and 2, figure legends and in the results section L255-256.

This is an appropriate sample size for our analysis, as shown in our Rhat estimations (<1.1) and posterior predictive checks (Supplementary material Fig. S2_1 to Fig. S2_4).

*I am not familiar with Bayesian framework, so pardon the question. How the effects can be considered meaningful (or significant, but it is not about semantics) if their 95% CI overlap zero?*

Not a problem. The “f” parameter (last column of tables) measures the proportion of the posterior distribution that has the same sign of the posterior mean. This would be comparable to the P value with an estimation with maximum likelihood. When a f value is >= 0.95 it means that there is a probability that the posterior distribution has the same sign of the mean. It is equivalent somehow to α = 0.05, in maximum-likelihood estimations. This is the reason why we indicate with * factors with an f value >0.95 and with a point those with f [0.90, 0.95]. Highest posterior density intervals (HPD) are built with quantiles 0.025 and 0.975, therefore, it will overlap 0 whenever f<0.975. Even when this happens, we can be quite sure that there is an effect if f is large (e.g. >=0.95).

*Figures needs major revisions. First, all figure captions should explain what is being showed, not provide the interpretation.*

As suggested, we have changed figure captions so that they provide a description of what is represented rather than an interpretation.

*The current version of Fig 1 is difficult to interpret, and the figure caption does not help as instead explaining what is shown, it presents the interpretation. Acorn size was categorial here with three levels, yet only two are shown. Then, if the acorn size was category, what is the point of showing it at y-axis which should be the place of response variable*

We believe that there is a misunderstanding with figure 1, probably because the legend was not clear enough and labels of x-axis could be confused with colour code. Fig 1 represents the size of acorns that were selected (or not) and removed (or not), under different environmental (i.e. presence/absence of ungulates, presence/absence predator scent and new/full moon). Colour code does not represent levels of acorn size, but mouse foraging decisions. Representing size in the y-axis, environmental conditions in the x-axis and foraging decisions (yes/no) in the colour code, allowed us to represent the interaction between acorn size and these environmental factors on mouse foraging choices. Figure 1 shows that acorn size only differed between selected and not selected acorns under “low stress conditions” (no ungulates, no predator scent). The same applies for removal. The difference between selection and removal is that though mice preferentially selected large acorns they tended to remove smaller ones.

We have rearranged the figure to avoid confusions between “yes/no” in the color code (foraging decisions) and in the x-axis (environmental conditions). Now for environmental conditions we refer to present/absent in the case of ungulates and predator scent; and new/full moon conditions. In addition, we have rewritten the figure legend and we hope it is clearer now.

*– I guess we are here mostily interested in probabilities? I suggest that all panels at Figure 1 show Probability at y-axis, acorns size at x-axis, and how the probability of each process changes with size depending on the treatment (ungulates, scent, moon etc).*

To represent probabilities, we need sample posterior distributions and for each acorn calculate the probability of selection and removal. Then, repeat the procedure N number of iterations and obtain a mean value per acorn. This would be a little bit circular with respect to the model because instead of showing raw values (as in the current figure 1), model predictions would be represented.

We have made a figure of probabilities; in case you feel it is absolutely necessarily to change it. However, we would prefer not to for two reasons. The first one, is that simulations are made with model estimates, and hence, the reader cannot evaluate how well model estimates reflect raw data. The second one, is that in the case of acorn selection, boxplots are very noisy. Acorn selection is modelled following a multinomial regression, and hence, probabilities not only depend on acorn size and environmental covariates; but also, on the number of acorns available. For instance, with no selection, when 15 acorns are available each one will have a probability of 0.067; whereas when 4 acorns are available it will be of 0.25. These simulations cannot tease apart such effects.

Fig. R1. Probability of acorn selection (upper panels) and removal (lower panels) for small (<3 g), medium ([3,6] g) and large (>6 g) acorns. Colour code represents whether the stressor (ungulates, predator scent and full moon, was present or not). For each acorn and foraging event, we estimated the probability of selection/removal by sampling 5000 iterations of posterior distributions. Then, we calculated mean values across iterations, with which we represented boxplots.

*Boxplots at Fig 2 should include data points in the background.*

Good point, added accordingly. Also, as suggested, we have rewritten the figure legend to make it clearer.

*L50: The net outcome of the interaction does not depend on whether seeds are consumed or cached, as seeds are usually both consumed and cached in each interaction. The key is the balance between predation and dispersal, and the balance of the benefit (improved recruitment) vs cost (predation and thus reduced recruitment).*

Right! For simplicity, the sentence now reads ‘The outcome of the interaction may be either mutualistic (dispersal) or antagonistic (predation) depending on the proportion of seeds consumed vs. cached and not retrieved [6]. (L51). We did not expand the sentence to include other components of benefits (i.e. the longer-term survival prospects of seedlings depending on microsite conditions) because these issues, currently well developed for mutualisms such as dispersal of fleshy-fruited plants by frugivores, are much less developed for synzoochorous interactions (review by Gómez et al. 2019). In fact, these authors remark in their review on nut dispersal effectiveness that data available refers to relatively short-term nut survival, not overall plant recruitment.

*L58: This sentence suggests that corvids are inefficient dispersers in oaks savannas, yet we have papers showing the contrary both in dehesas (Baroja et al: https://besjournals.onlinelibrary.wiley.com/doi/abs/10.1111/1365-2745.13642) as well as in oak savannas in other regions (e.g. Pesendorfer et al, papers from California).*

Indeed, corvids can be important dispersers in open areas (e.g. magpies). We have reformulated the beginning of this paragraph accordingly (L59-61).

*L70: “In the absence of stress… “ this sentence is oversimplification and sounds like rodents never store seeds in the absence of stress, which is not true. Simply put, if there is lots of food (no stress) we do expect that rodents will start to store.*

Thanks for the remark. We have modified the sentence that now reads ‘In the absence of stress, rodents usually act as efficient seed predators as they consume, immediately or soon afterwards, most seed crops under the canopy of mother trees’ L77-79.

*L112: what does it mean that they were opened?*

As quoted in the preceding sentence, ‘Dehesas are savanna-like man-made habitats resulting from shrub removal and tree thinning and pruning to enhance herb growth for livestock [41].’ L115-117. This implicitly means that they should be ‘opened’ from closed forests. Now the sentence reads ‘The studied dehesas were opened from the original Mediterranean forests in the late 1950s’

*L121: averages of both areas are needed to support a statement that they have similar tree abundance*

We intended to mean that tree densities were the same inside and outside each exclosure, not that tree density was uniform across the study area. Now we provide mean and SE values for tree density (No./ha) around the 50 focal trees in each site x exclosure combination where we trapped mice. Overall mean was 20.4 trees/ha, but site 1 has 30 trees/ha both inside and outside the exclosure and site 2, 7 trees/ha. The sentence now reads ‘Average tree density was 20.4 trees ha-1, although site 1 had higher mean density than site 2 (30.0±2.6 and 30.1±3.4 inside and outside ungulate exclosure in site 1; and 7.4±0.4 and 7.4±0.3 in site 2; mean±SE). Shrub cover was <1%, as measured on aerial photographs and vegetation surveys both under canopies and outside them [24].’ L126-131.

*Reviewer #3: This is a comprehensive study as to the joint effect of habitat structure, competition and predation risk on dispersal effectiveness in an oak-mice system. They found that intermediate stress (presence of predator or grazer) could increase dispersal effectiveness and then facilitated interaction towards the mutualistic side, providing new evidences on conditional mutualism. I think this is a good contribution to the study of the field. I have only a few revision suggestions, mainly by including a few previous similar studies:*

We are grateful for the support to our work.

*Line: 147-148. The wire-linked plastic seed tagging method was proposed by Xiao et al. (2006). You need to include the original reference: Xiao et al, 2006, Forest Ecology and Management, 223:18–23；*

Changed accordingly, L158.

*Line 296-299. You found ungulate presence would benefit dispersal of oak acorns. A previous study had similar results. You should include the reference in the discussion: Zhang et al. (2009), Wildlife Research, 36: 610–616;*

Thank you, we had missed this important reference. It now included L315.
